# Steric augmentation of three-coordinate Cu(i) β-diketiminate isocyanide chromophores to achieve microsecond excited-state lifetime

**DOI:** 10.1039/d5sc09786j

**Published:** 2026-02-24

**Authors:** Ashish Kumar, Dooyoung Kim, Sean P. Dunphy, Erin N. Lewis, Joshua S. Figueroa, Thomas S. Teets

**Affiliations:** a Department of Chemistry, University of Houston Houston Texas 77204 USA tteets@uh.edu; b Department of Chemistry and Biochemistry, University of California, San Diego La Jolla California 92093 USA

## Abstract

Copper(i) complexes are promising alternatives to noble metal-based photosensitizers. A recently developed class of three-coordinate copper(i) charge-transfer chromophores, pairing β-diketiminate ligands with aryl isocyanides, exhibits excited-state lifetimes that are responsive to the steric profile of the isocyanide, reaching a value of nearly 300 nanoseconds when a substituted *m*-terphenyl isocyanide was used. In this work, we show that extreme steric augmentation of the β-diketiminate and *m*-terphenyl isocyanide ligands can lead to further improvements in excited-state lifetime, with one example surpassing 1 µs. This study includes a series of eight compounds of the general formula Cu(RNacNac)(CN-Ar^X2^), where RNacNac is the substituted β-diketiminate and CN-Ar^X2^ is the substituted *m*-terphenyl isocyanide. All eight compounds are crystallographically characterized, and their excited-state properties are thoroughly evaluated by a combination of steady-state and time-resolved photoluminescence studies. This combined structural/photophysical approach unveils a correlation between common steric parameters, derived from buried volume (%*V*_bur_) and solid angle analysis, and the excited-state lifetime. Our investigation reveals that the steric profile of the β-diketiminate has a much larger impact on the steric profile and lifetime, whereas the *m*-terphenyl isocyanide substituents have an unsystematic and subtle influence on these two correlated parameters. Nevertheless, judicious pairing of the two ligands is critical for achieving long excited-state lifetimes.

## Abstract 2

Augmenting the steric profile of both the β-diketiminate ligand and the *m*-terphenyl isocyanide increases the excited-state lifetime in heteroleptic copper(i) complexes, reaching the microsecond range in one case.

## Graphical abstract

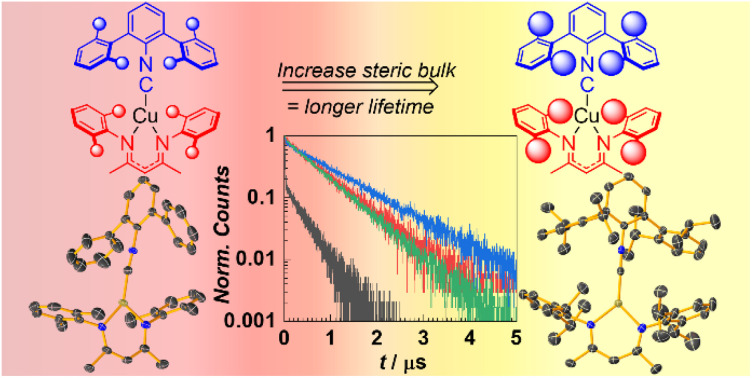

## Introduction

Molecular photosensitizers have been employed in various fields such as energy conversion and synthetic organic chemistry.^1–3^ The well-established photosensitizers based on precious transition metals, notably iridium(iii),^4,5^ ruthenium(ii),^6,7^ and platinum(ii)^8,9^ are particularly effective in photocatalysis applications. However, their expense and low abundance remain concerns, spurring interest in more economical replacements, which include first-row transition metal-based coordination compounds^10–12^ and metal-free organic chromophores.^13–15^ Among many first-row transition metals that have been investigated, complexes-based on copper(i) have risen to prominence due to their d^10^ valence electronic configuration, which allows the desired charge-transfer state (usually metal-to-ligand charge transfer, MLCT) to be the lowest-energy state, avoiding rapid decay *via* deleterious, low-lying metal-centered (MC) states. While copper(i) avoids the problem of deactivating MC states, it is nonetheless a challenge to achieve long charge-transfer excited-state lifetimes, due to a pseudo-Jahn Teller distortion that occurs upon excitation.^16^ The most common classes of copper(i) photosensitizers are four-coordinate structures with two 1,10-phenanthroline derivatives (N^N) or one N^N ligand paired with a diphosphine.^17–20^ In the homoleptic [Cu(N^N)_2_]^+^ analogues, the most widespread and robust strategy for increasing the excited-state lifetime involves strategically placed sterically blocking substituents, usually alkyl groups, at the 2- and 9-positions or other locations of the phenanthroline.^21^

Heteroleptic copper(i) photosensitizers have more recently become prominent,^22–26^ with our group's efforts involving complexes supported by β-diketiminate (RNacNac) ligands.^27–30^ Ligand steric effects have also been effective in these complexes at improving excited-state lifetimes,^27,28^ with the more recently developed three-coordinate Cu(RNacNac)(CN-Ar^X2^) (CN-Ar^X2^ = aryl isocyanide) class of compounds also exhibiting notable photocatalytic reactivity.^28^ In our inaugural work on isocyanide-terminated complexes, we focused exclusively on steric modifications to the isocyanide, using a common cyclohexyl-substituted β-diketiminate (CyNacNac) in the series of complexes. In the most unhindered analogue, a short lifetime of 9.3 ns was observed, which increased almost 30-fold up to 276 ns in one of the compounds with a substituted *m*-terphenyl isocyanide. In the present work, we expand this sterically-driven approach to include steric modifications on the RNacNac ligand, and incorporate even bulkier *m-*terphenyl isocyanides, previously introduced in the context of low-coordinate metal complexes for small-molecule activation.^31–33^ These modifications influence the redox potentials, excited-state energies, and photoluminescence wavelengths, but more importantly, have pronounced impacts on the excited-state decay dynamics. We show that the well-known ligand steric parameters percent buried volume (%*V*_bur_)^34–36^ and solid angles^37^ correlate with the observed excited-state lifetimes. The steric profile of the RNacNac ligand, varied by using 2,6-dialkyl-substituted phenyl rings as the *N*-substituents (R), has a much bigger influence than the isocyanide on the overall steric profile and the excited-state lifetime. Thus, steric augmentation of the RNacNac ligand alone brings about a >2.5-fold increase in lifetime. Nevertheless, the substitution pattern of the *m*-terphenyl isocyanide can also have subtle and beneficial effects, such that one complex in this series exhibits an excited-state lifetime of 1.03 µs, an almost 4-fold increase over our previous outcomes with this class of compounds. This work demonstrates that pushing the envelope with respect to ligand design and steric profile can have beneficial effects on the photophysical properties in this emerging class of earth-abundant photosensitizers.

## Results and discussion

### Synthetic procedures

**Scheme 1:**
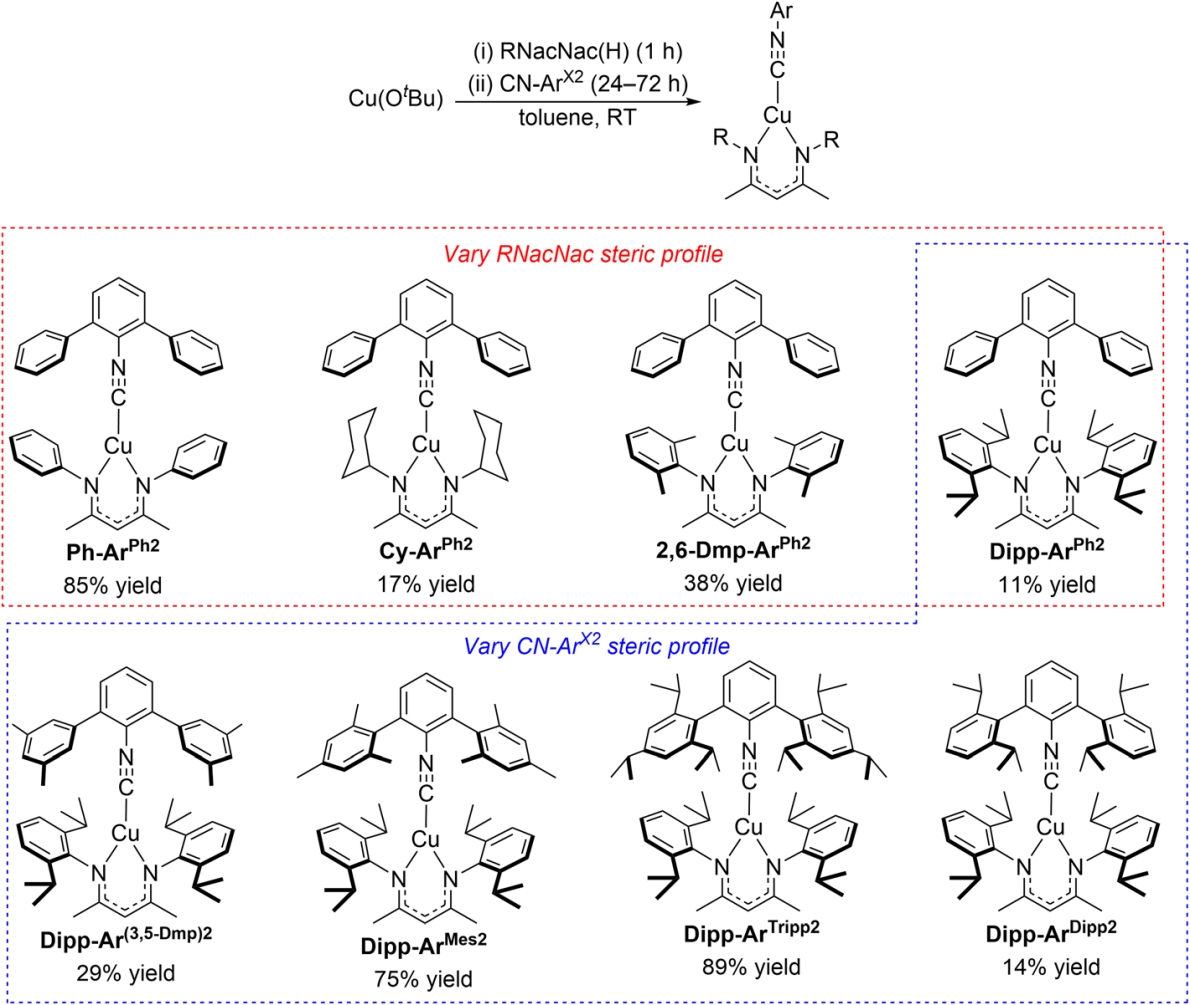
Synthesis and chemical structures of three-coordinate heteroleptic Cu(RNacNac)(CN-Ar^X2^) complexes.

The synthetic procedure for the eight heteroleptic copper(i) complexes presented here is outlined in Scheme 1. Being air-sensitive and moisture-sensitive, a nitrogen-filled glovebox was used to carry out all synthesis and purification procedures. In the first step, an equimolar mixture of CuO^*t*^Bu and the protonated RNacNac(H) proligand was stirred in toluene at room temperature for 1 h. Later, 0.7 equiv of the respective isocyanide was added, and the resulting mixture was stirred for 24–72 h at room temperature or 70 °C in a sealed tube. After workup, the compounds were purified by crystallization from toluene/pentane or pure pentane at −30 °C. The isolated yields of the yellow solid products are in the range of 11–89%, using the isocyanide ligand as the limiting reagent. The chemical structures of the eight new complexes are depicted in Scheme 1 as well, and they comprise four different RNacNac ligands and five different *m*-terphenyl isocyanides. The compounds are abbreviated with the naming scheme R-Ar^X2^, where R represents the *N* and *N*′ substituents of the RNacNac ligand (Ph = phenyl, Cy = cyclohexyl, 2,6-Dmp = 2,6-dimethylphenyl, Dipp = 2,6-diisopropylphenyl) and X indicates the flanking aryl rings of the *m*-terphenyl isocyanide (Ph, 3,5-Dmp = 3,5-dimethylphenyl, Mes = mesityl, Tripp = 2,4,6-triisopropylphenyl, and Dipp). In the top series of four compounds highlighted in the red box in Scheme 1, the RNacNac is varied while keeping the same unsubstituted *m*-terphenyl isocyanide (CN-Ar^Ph2^). Having identified DippNacNac as promoting the longest-lived excited states (see below), the five DippNacNac complexes highlighted with the blue box vary the substitution pattern on the *m*-terphenyl isocyanide. The complexes were spectroscopically characterized by ^1^H and ^13^C{^1^H} NMR (Fig. S1–S16) and FT-IR (Fig. S17–S29). NMR spectra are consistent with the proposed structures and suggestive of two-fold symmetry in each case. The FT-IR spectra show a clear C≡N stretching band in the range of 2102–2121 cm^−1^, with small shifts from the respective free isocyanide that can be positive or negative (Table S1).

### Structural characterization

**Fig. 1:**
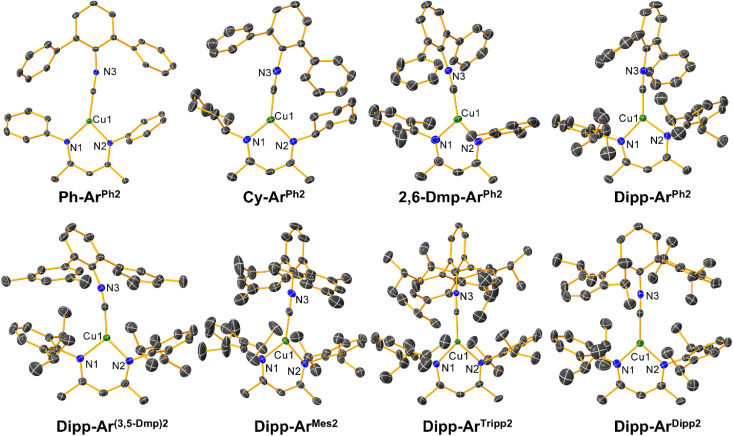
Molecular structures of the Cu(RNacNac)(CNAr^X2^) complexes, determined by single-crystal X-ray diffraction. Thermal ellipsoids are shown at the 50% probability level, and the hydrogen atoms have been omitted for clarity.

The molecular structures of all eight copper complexes were determined by single-crystal X-ray diffraction and are shown in Fig. 1. A summary of the crystallographic data is provided in Tables S2–S9, along with key bond distances and angles provided in Table S10 of the SI. All complexes exhibit planar Y-shaped geometries, with the two RNacNac Cu–N distances separated by no more than 0.02 Å. The Cu–C≡N angles span from 170.41(19)° to 178.94(18)°, indicating a small amount of π-backbonding from the Cu center to the isocyanide. The dihedral angles between the mean plane of the Cu-RNacNac chelate ring (Cu–N–C–C–C–N) and the mean plane of the central ring of the *m*-terphenyl isocyanide were determined, and the values indicate that the complexes are likely conformationally flexible. The dihedral angles are between 35.64–89.45°, with seemingly no systematic dependence on the ligand steric profile (Table S11).

Using the SambVca 2.1 program,^35^ the percent buried volumes (%*V*_bur_, Table 1) and topographical steric maps (Fig. S30) of each ligand in the eight complexes were obtained. %*V*_bur_ is most commonly used in the context of catalysis, and it represents the percentage of the sphere volume around the metal center (*r* = 3.5 Å) that is occupied by each ligand.^34,38,39^ The sum of the two %*V*_bur_ values, Σ%*V*_bur_, is also included in Table 1 to represent the total degree to which the metal is shielded by the two ligands in each complex. The buried volume analysis reveals that variations to the RNacNac ligand have a much larger impact on the total %*V*_bur_. Specifically, the smallest PhNacNac ligand in Ph-Ar^Ph2^ exhibits a %*V*_bur_ of 44.8%, which increases to 59.0–62.9% in the DippNacNac complexes. In contrast, the %*V*_bur_ values determined for the *m*-terphenyl isocyanide ligands, where the steric bulk is situated further from the metal center on account of the linear C≡N moiety, span a much smaller range of 19.0–21.8% with seemingly no systematic dependence on the substitution pattern of the *m*-terphenyl group. It also appears that the substituent on the RNacNac ligand can influence the aryl isocyanide and “push” some of its bulk away from the metal center. As an illustration of this subtle effect, the unsubstituted CN-Ar^Ph2^ ligand has a %*V*_bur_ of 21.8% in Ph-Ar^Ph2^, but with the bulkier RNacNac in Dipp-Ar^Ph2^ that value drops to 20.4%. Across the entire series, the Σ%*V*_bur_ values span 66.6–82.6%, with the %*V*_bur_ of the RNacNac ligand falling in the order PhNacNac < CyNacNac < DmpNacNac < DippNacNac.

**Table 1:** Summary of the buried volume (%*V*_bur_) analysis for the Cu(RNacNac)(CN-Ar^X2^) complexes^a^

	RNacNac %*V*_bur_	Isocyanide %*V*_bur_	Σ%*V*_bur_
Ph-Ar^Ph2^	44.8	21.8	66.6
Cy-Ar^Ph2^	50.3	21.0	71.3
2,6-Dmp-Ar^Ph2^	52.5	21.6	74.1
	53.0	20.9	73.9
	52.5	21.2	73.7
	52.6	20.9	73.5
Dipp-Ar^Ph2^	59.0	20.4	79.4
Dipp-Ar^(3,5-Dmp)2^	60.6	19.7	80.3
	60.8	20.5	81.3
Dipp-Ar^Mes2^	62.1	19.0	81.1
Dipp-Ar^Tripp2^	62.9	19.7	82.6
Dipp-Ar^Dipp2^	61.1	20.1	81.2

a In 2,6-Dmp-Ar^Ph2^ and Dipp-Ar^(3,5-Dmp)2^, which each have more than one crystallographically independent molecule in the asymmetric unit, separate %*V*_bur_ values are reported for each independent molecule.

### Cyclic voltammetry

**Fig. 2:**
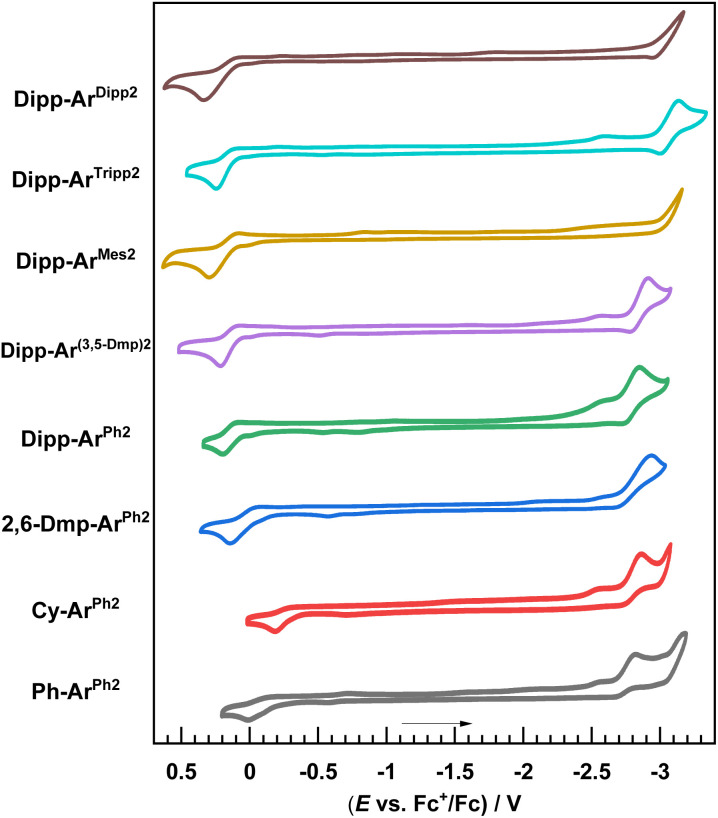
Overlaid cyclic voltammograms of the Cu(RNacNac)(CN-Ar^X2^) complexes. The cyclic voltammograms were recorded in THF with a 0.1 M NBu_4_PF_6_ electrolyte using a glassy carbon working electrode, platinum wire counter electrode, and silver wire pseudoreference electrode, with a scan rate 0.1 V s^−1^. Potentials were referenced against an internal standard of ferrocene.

Cyclic voltammetry was performed to investigate the effects of the ligand substituents on the redox potentials. CVs are shown in Fig. 2 with the summary of reduction potentials in Table 2. Upon sweeping to positive potential, a one-electron oxidation is observed, which can be formalized as a Cu^ii^/Cu^i^ couple but likely also invovles substantial redox activity of the RNacNac ligand.^29^ The potential associated with this oxidation wave, *E*^ox^, varies significantly across the series. With the most electron-rich CyNacNac in Cy-Ar^Ph2^, *E*^ox^ = −0.18 V *vs.* ferrocenium/ferrocene (reported as the peak potential *E*_p,c_), whereas in the remaining complexes with aryl-substituted RNacNac ligands this potential is positive. *E*^ox^ progressively shifts more positive as the RNacNac steric bulk is increased, for example, Ph-Ar^Ph2^ (0.01 V) < 2,6-Dmp-Ar^Ph2^ (0.13 V) < Dipp-Ar^Ph2^ (0.19 V). This positive shift with increasing steric bulk is analogous to that observed in other classes of copper(i) complexes;^27,40–42^ the complex acquires Cu(ii) character upon oxidation, which is Jahn–Teller active, and the bulky ligands restrict the Jahn–Teller distortion and lock the complex into a geometry that is unfavorable for Cu(ii), making oxidation more difficult. Interestingly, the steric profile of the isocyanide, which has no significant impact on Σ%*V*_bur_ (see above), does influence *E*^ox^. In the DippNacNac series, the potential shifts by 50–160 mV in the complexes with 2,6-dialkyl or 2,4,6-trialkyl substitution on the isocyanide (Dipp-Ar^Mes2^, Dipp-Ar^Tripp2^, and Dipp-Ar^Dipp2^), compared to Dipp-Ar^Ph2^, which lacks alkyl substituents. Reduction waves occur at very negative potentials (*E*^red^) near the edge of the solvent window. Clear reduction waves were not observed in Dipp-Ar^Mes2^ and Dipp-Ar^Dipp2^, and for the rest *E*^red^ = −2.82 to −3.14 V. Alkyl substituents on the *m*-terphenyl isocyanide appear to slightly destabilize the LUMO and cathodically shift *E*^red^, but in most cases this is a small effect. HOMO–LUMO gaps were estimated as the difference between *E*^ox^ and *E*^red^, smallest in Cy-Ar^Ph2^ (Δ*E*_H–L_ = 2.76 eV) and largest in Dipp-Ar^Tripp2^ (Δ*E*_H–L_ = 3.38 eV).

**Table 2:** Summary of reduction potentials^a^

	*E* ^ox^/V^b^	*E* ^red^/V^c^	Δ*E*_H–L_^d^/eV
Ph-Ar^Ph2^	0.01	−2.82	2.83
Cy-Ar^Ph2^	−0.18	−2.94	2.76
2,6-Dmp-Ar^Ph2^	0.13	−2.94	3.07
Dipp-Ar^Ph2^	0.19	−2.85	3.04
Dipp-Ar^(3,5-Dmp)2^	0.21	−2.92	3.13
Dipp-Ar^Mes2^	0.30	N.D.	—
Dipp-Ar^Tripp2^	0.24	−3.14	3.38
Dipp-Ar^Dipp2^	0.35	N.D.	—

a Potentials are reported in volts (V) *vs.* the ferrocene redox couple (Fc^+^/Fc). *E*^ox^ is associated with the Cu^i^(RNacNac^˙^)/Cu^i^(RNacNac^−^) couple, and *E*^red^ refers to the isocyanide-based reduction process.

b Irreversible wave. *E*_p,a_ is reported.

c Irreversible wave. *E*_p,c_ is reported.

d Estimated electrochemical HOMO–LUMO gap, determined as *E*^ox^–*E*^red^.

### Photophysical properties

**Fig. 3:**
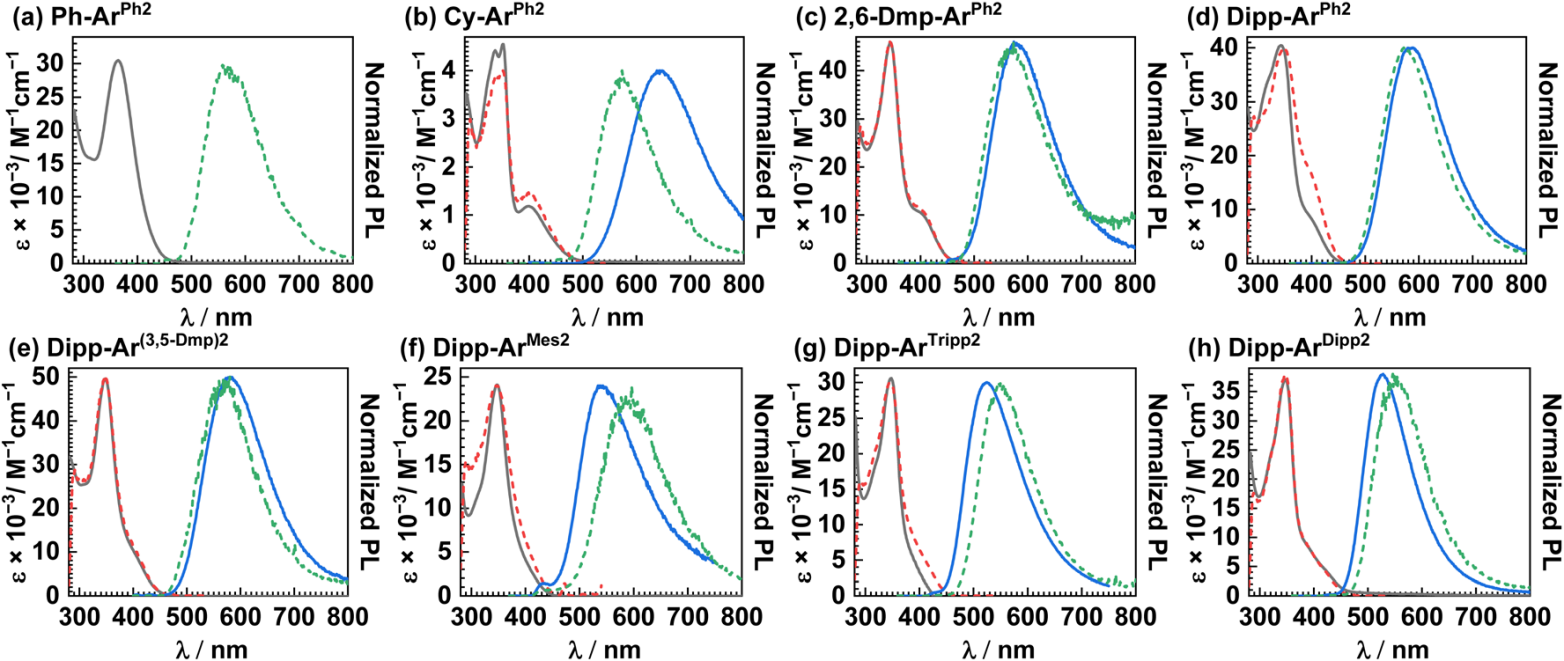
Overlaid UV-vis absorption (black solid line) and excitation (red dashed line) spectra, plotted with photoluminescence spectra at 298 K (blue solid) and 77 K (green dashed line). (a) Ph-Ar^Ph2^, (b) Cy-Ar^Ph2^, (c) 2,6-Dmp-Ar^Ph2^ (d) Dipp-Ar^Ph2^, (e) Dipp-Ar^(3,5-Dmp)2^, (f) Dipp-Ar^Mes2^, (g) Dipp-Ar^Tripp2^ and (h) Dipp-Ar^Dipp2^. All spectra were recorded in toluene under inert conditions.

UV-vis absorption and photoluminescence experiments were conducted on the eight copper(i) complexes in toluene. The UV-vis absorption spectra, excitation spectra, and photoluminescence spectra at 298 and 77 K are shown in Fig. 3, with data summarized in Table 3. Table 3 also includes data from four previously published, closely-related compounds,^28^ with the discussion in the text mainly focused on the analogues first reported here. The excitation spectra overlap well with the absorption spectra, indicating a single emitting species. The absorption maxima occur in the range of 343–363 nm, with a molar absorption coefficient (*ε*) of 4.6 × 10^3^ M^−1^ cm^−1^ in Cy-Ar^Ph2^ and values that increase by an order of magnitude, 3.0–4.9 × 10^4^ M^−1^ cm^−1^ in the remaining complexes, on account of the more extensive conjugation in aryl-substituted RNacNac ligands. These high-energy transitions are assigned to π → π* transitions involving one or both conjugated ligands. A clear longer-wavelength band near 400 nm is seen in most cases. In Cy-Ar^Ph2^ this peak appears as a distinct maximum, whereas in the remaining complexes it either appears as a shoulder or is completely obscured by the more intense π → π* band. This low-energy band is assigned to a HOMO→LUMO charge-transfer (^1^CT) transition. There is not a pronounced or systematic dependence of either absorption wavelength on the substitution pattern of the two ligands. Photoluminescence measurements were conducted at both 298 K and 77 K, and as shown in Fig. 3, the least hindered complex Ph-Ar^Ph2^ is only photoluminescent at the lower temperature. The remaining complexes have appreciable luminescence at 298 K. At both temperatures a single featureless band is observed. There are two key differences between Cy-Ar^Ph2^ and the newly reported aryl-substituted RNacNac complexes. First, Cy-Ar^Ph2^ exhibits room-temperature PL that is significantly red-shifted from the complexes with aryl-substituted RNacNac ligands, on account of the smaller HOMO–LUMO gap brought on by the electron-rich cyclohexyl rings. Second, the PL of Cy-Ar^Ph2^ at 77 K is substantially blue-shifted (72 nm, *ca.* 2000 cm^−1^) from that recorded at 298 K, a rigidochromic shift that is typical of phosphorescence from charge-transfer excited states.^43^ In contrast, the complexes with 2,6-DmpNacNac or DippNacNac exhibit PL spectra at 77 K that are either minimally blue-shifted (Fig. 3c–e) or noticeably red-shifted (Fig. 3f–h) compared to the data at 298 K. The origin of this effect is not entirely clear, but likely indicates that structural distortion of the *T*_1_ state in fluid solution is inhibited in the more sterically crowded analogues, and suggests that a thermally activated process may be involved in the PL at 298 K. Augmenting the steric profile of the isocyanide causes a blue-shift in the PL, with Dipp-Ar^Tripp^ having the shortest-wavelength PL (Table 3), consistent with this complex having the largest electrochemical HOMO–LUMO gap (Δ*E*_H–L_) in the series (Table 2). Nevertheless, on the basis of the PL maxima at 77 K, we estimate that the *T*_1_ energies in these compounds differ by no more than 0.15 eV across the series, indicating that substituent effects on the energetics of the luminescent transitions are minor, in the same manner that the UV-vis absorption wavelengths show small variations in this series of complexes.

**Table 3:** Summary of photophysical data

	UV-vis absorption, *λ*/nm (*ε* × 10^−3^/M^−1^ cm^−1^)	PL, *λ*/nm		*Φ*_PL_(%)	*τ*/ns	*k*_r_ a/s^−1^	*k*_nr_ a/s^−1^
		298 K	77 K				
**Newly reported complexes**							
Ph-Ar^Ph2^	363 (30)	—	566	—	—	—	—
Cy-Ar^Ph2^	337 (4.4), 351 (4.6), 400 (1.2)	644	572	0.5	216	2 × 10^4^	5 × 10^6^
2,6-Dmp-Ar^Ph2^	343 (46), 394 (sh) (11)	580	569	3	376	8 × 10^4^	3 × 10^6^
Dipp-Ar^Ph2^	343 (40), 394 (sh) (9.1)	586	570	7	664	1 × 10^5^	1 × 10^6^
Dipp-Ar^(3,5-Dmp)2^	348 (49), 399 (sh) (11)	579	570	7	733	1 × 10^5^	1 × 10^6^
Dipp-Ar^Mes2^	347 (24)	539	589	0.4	47	8 × 10^4^	2 × 10^7^
Dipp-Ar^Tripp2^	348 (31)	524	549	4	672	6 × 10^4^	1 × 10^6^
Dipp-Ar^Dipp2^	347 (37), 391 (sh) (8.2), 420 (sh) (4.5)	526	556	4	1030	4 × 10^4^	9 × 10^5^

**Previously reported complexes** b							
Cy-Dmp	319 (11), 335 (12), 351 (13), 390 (3.4)	548	500	0.08	9	9 × 10^4^	1 × 10^8^
Cy-Dipp	319 (sh) (23), 337 (28), 350 (30), 393 (6.4)	547	513	0.3	23	1 × 10^5^	5 × 10^7^
Cy-Ar^(3,5-Dmp)2^	323 (sh) (28), 337 (31), 351 (33), 391 (8.0)	635	560	9	276	3 × 10^5^	3 × 10^6^
Cy-Ar^(3,5-D^*^t^*^bup)2^	324 (13), 333 (13), 353 (13), 404 (3.7)	582	590	2	97	2 × 10^5^	1 × 10^7^

a *k*_r_ = *Φ*_PL_/*τ*; *k*_nr_ = (1−*Φ*_PL_)/*τ*.

b Chemical structures are shown in Fig. S31; data was previously reported in ref. 28.

Photoluminescence lifetimes, measured by time-correlated single photon counting (TCSPC), reveal that the ligand steric profile has a profound impact on the lifetime of the ^3^CT state. Time-resolved photoluminescence decay traces are provided in Fig. S32–S38, and UV-vis absorption spectra recorded before and after the measurements (Fig. S39–S45) indicate that the complexes are photostable and that the lifetime measurements are not influenced by photodecomposition. The effect of the RNacNac substituents is especially pronounced, as evident from the trends in the R-Ar^Ph2^ series. The least hindered complex, Ph-Ar^Ph2^, is not luminescent at room temperature, indicating a very short lifetime. As the %*V*_bur_ of the RNacNac increases (see Table 1), the excited-state lifetime also increases: Cy-Ar^Ph2^ (*τ* = 216 ns) < 2,6-Dmp-Ar^Ph2^ (*τ* = 376 ns) < Dipp-Ar^Ph2^ (*τ* = 664 ns). Having identified DippNacNac as the best choice for promoting long-lived ^3^CT states, we then investigated the Dipp-Ar^X2^ series to determine whether increased steric bulk on the isocyanide could exert additional beneficial effects. While the effects of the isocyanide substituents are less pronounced and not as systematic, there is some influence. The complex Dipp-Ar^Mes2^ stands out as an outlier, giving a markedly shorter lifetime of 47 ns, possibly indicating that methyl C–H stretching motions of the mesityl ring are prominent in nonradiative decay. Most of the remaining complexes with alkyl substituents on the *m*-terphenyl isocyanide have only minimally different lifetimes: *τ* = 664 ns in Dipp-Ar^Ph2^, 672 ns in Dipp-Ar^Tripp2^, and 733 ns in Dipp-Ar^(3,5-Dmp)2^. In Dipp-Ar^Dipp2^, where both ligands are decorated with 2,6-diisopropylphenyl substituents, we observed a pronounced beneficial effect of the isocyanide substituents, achieving a lifetime of 1.03 µs, eclipsing the microsecond scale. The photoluminescence quantum yields (*Φ*_PL_) are low to moderate, spanning 0.5–7%, with most complexes having broadly similar values of 3–7%.

## Discussion

Having previously demonstrated pronounced isocyanide steric effects on the observed excited-state lifetimes of Cu(CyNacNac)(CN-Ar^X2^) complexes,^28^ in this work we sought to investigate whether analogous modifications to the RNacNac ligand are likewise beneficial, and push the extremes to which we could augment the steric profile in this class of complexes. To achieve the former goal, we used the straightforward approach of replacing the cyclohexyl ring in CyNacNac with substituted phenyl rings, where modification with alkyl substituents at the 2- and 6-positions can greatly increase the steric profile, a feature that has widely been used in the contexts of small-molecule activation and catalysis.^44^ To achieve the latter goal, we combined steric modifications on the RNacNac with a class of bulky *m*-terphenyl isocyanide ligands developed and popularized by Figueroa's group.^31–33^ Importantly, our synthetic investigations (Scheme 1) revealed that all combinations of RNacNac and CN-Ar^X2^ ligands could be readily assembled, with no steric limitations of the general synthetic route. Isolated yields varied considerably, although we attribute the lower obtained yields to losses during crystallization, given the high solubility of some of the complexes.

**Fig. 4:**
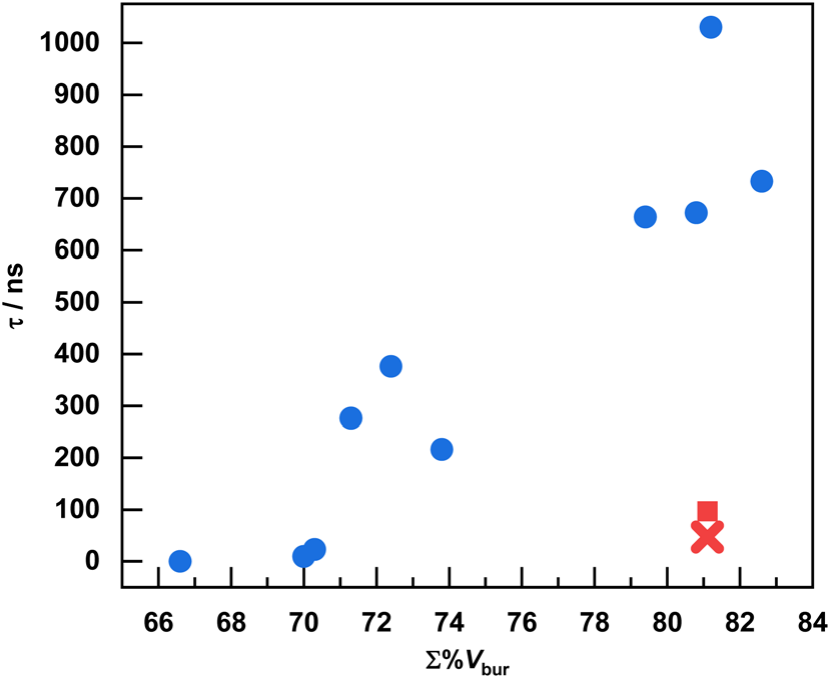
Excited-state lifetimes of Cu(RNacNac)(CN-Ar^X2^) complexes plotted *vs.* the sum of the percent buried volume of the two ligands (Σ%*V*_bur_). For the complex Ph-Ar^Ph2^, where there was no PL observed, the lifetime was taken to be 0. For Dmp-Ar^Ph2^ and Dipp-Ar^(3,5-dmp)2^, which have more than one crystallographically independent molecule, the average of the Σ%*V*_bur_ values is used in the plot. This plot also includes four Cu(CyNacNac)(CN-Ar^X2^) complexes previously reported by our group.^28^ The extreme outliers Dipp-Ar^Mes2^ (red “x”) and Cy-Ar^(3,5-D^*^t^*^bup)2^ (red square, previously reported) are denoted.

To more quantitatively evaluate the steric profiles of the chosen ligands, and to correlate steric bulk with the observed excited-state lifetimes, we used the common steric parameter %*V*_bur_. As indicated in Table 1, the majority of the ligand steric bulk arises from the RNacNac ligands, and the significant differences in total buried volume (Σ%*V*_bur_, the sum of both ligands) arise almost exclusively from the variations in the RNacNac ligand. Fig. 4 demonstrates the relationship between Σ%*V*_bur_ and the excited-state lifetime, determined by time-resolved PL. Fig. 4 also includes the results from four previously reported Cu(CyNacNac)(CN-Ar^X2^) complexes, which fit the general trend observed here. There are two obvious outliers in the plot – the newly reported Dipp-Ar^Mes2^, shown as a red “x” in Fig. 4, and previously reported Cy-Ar^(3,5-D^*^t^*^bup)2^ (3,5-D^*t*^bup = 3,5-di(*tert-*butyl)phenyl, shown as a red square). Both of these compounds have Σ%*V*_bur_ = 81.1% but with *τ* < 100 ns. The remaining complexes show a clear positive correlation between Σ%*V*_bur_ and *τ*. The correlation is not smooth and is better interpreted in terms of three bins of data. For complexes where Σ%*V*_bur_ is *ca.* 70% or less, we observe short lifetimes of <25 ns. In cases where Σ%*V*_bur_ is *ca.* 71–75%, intermediate lifetimes in the 216–376 ns range are noted, and when Σ%*V*_bur_ approaches or exceeds 80%, *τ* > 650 ns, excepting the outliers. The complex Dipp-Ar^Dipp2^ deviates from the trend in a positive way, giving it the longest lifetime of *τ* = 1.03 µs. The shorter lifetimes in the complexes with *para*-substitution on the *m*-terphenyl isocyanide (Dipp-Ar^Mes2^ and Dipp-Ar^Tripp2^) may originate from steric clash between the *para* substituents and the DippNacNac substituents, resulting in less structural rigidity. Similar effects have been seen in surface-binding studies on this ligand class, where the *para* substituents can clash with surface atoms result in curvature-selective binding.^45^

The above discussion focuses on %*V*_bur_, but there are other steric parameters that could also be considered. To test the robustness of the correlation between excited-state lifetime and ligand steric bulk, we also used the solid angle method, which gives a parameter abbreviated as *G*_M_(complex) that reports the percentage of the metal's coordination sphere that is shielded by the ligands.^37^ As shown in Table S13 and Fig. S48, a similarly strong correlation is observed when the excited state lifetime is plotted against *G*_M_(complex), with the same two compounds (Dipp-Ar^Mes2^ and Cy-Ar^(3,5-D^*^t^*^bup)2^) representing the most obvious outliers. We also considered correlations between *τ* and the other experimental measurables *E*^ox^ (Fig. S47) and the triplet excited-state energy *E*(*T*_1_) (Fig. S48), but no clear correlations were observed in either of those cases.

The combination of photoluminescence quantum yields (*Φ*_PL_) and lifetimes (*τ*) allow us to determine radiative (*k*_r_) and nonradiative (*k*_nr_) rate constants, also summarized in Table 3. The variations in lifetimes we observe are primarily due to differences in *k*_nr_. The *k*_r_ values for the aryl-substituted RNacNac complexes span a comparatively narrow range of 4 × 10^4^ s^−1^ to 1 × 10^5^ s^−1^, slightly higher than that of Cy-Ar^Ph2^ (*k*_r_ = 2 × 10^4^ s^−1^). In contrast, *k*_nr_ for the complex with the shortest measurable lifetime (Dipp-Ar^Mes2^, *τ* = 47 ns, *k*_nr_ = 2 × 10^7^ s^−1^) is over 20× larger than that of Dipp-Ar^Dipp2^ (*τ* = 1.03 µs, *k*_nr_ = 9 × 10^5^ s^−1^). Thus, Dipp-Ar^Dipp2^ has the longest excited-state lifetime in the series largely due to its small *k*_nr_ value. These observations are entirely consistent with the conventional views on the effects of sterically crowding substituents in copper(i) charge-transfer complexes, where the ligand steric bulk restricts the excited-state Jahn–Teller distortion, suppressing nonradiative decay and enabling a longer-lived excited state.

## Conclusions

This work shows that extreme steric tuning of heteroleptic Cu(RNacNac)(CN-Ar^X2^) complexes, a promising class of earth-abundant charge-transfer chromophores, can lead to pronounced increases in excited-state lifetime. Achieving long excited-state lifetimes in copper(i) complexes is a persistent challenge, mainly due to the charge-transfer excited state being Jahn–Teller active, and this work leverages prior insights which have shown that sterically blocking substituents can restrict the Jahn–Teller distortion and lengthen the lifetime of the charge-transfer state. A key insight from this work is that the total steric profile of the ligands, evaluated *via* percent buried volume (Σ%*V*_bur_) or solid angles, shows a general positive correlation with excited-state lifetime. The substituents on the RNacNac ligand are the strongest determinants of both the steric profile and *τ*. While the effects of the more remote *m*-terphenyl isocyanide substituents are more subtle, in one complex, Dipp-Ar^Dipp2^, where both the RNacNac and the isocyanide include bulky 2,6-diisopropylphenyl groups, the steric profiles of the two ligands beneficially combine to push the excited-state lifetime beyond 1 µs. Future efforts in this class of compound will include applications in photocatalysis enabled by the long charge-transfer lifetimes and comparatively strong reducing ability of these complexes, and combining the insights from this work with additional ligand modifications designed to decrease the HOMO–LUMO gap and extend the visible absorption to longer wavelengths.

## Author contributions

Ashish Kumar: formal analysis, investigation, validation, visualization, writing – original draft, writing – review & editing. Dooyoung Kim: formal analysis, investigation, writing – review & editing. Sean. P. Dunphy: investigation, resources. Erin N. Lewis: investigation, resources. Joshua S. Figueroa: funding acquisition, project administration, writing – review & editing. Thomas S. Teets: conceptualization, formal analysis, funding acquisition, project administration, visualization, writing – review & editing.

## Conflicts of interest

There are no conflicts to declare.

## Supplementary Material

SC-OLF-D5SC09786J-s001

SC-OLF-D5SC09786J-s002

## Data Availability

CCDC 2464277–2464284 contain the supplementary crystallographic data for this paper.^46*a*–*h*^ The data supporting this article have been included as part of the supplementary information (SI). Supplementary information: experimental details, NMR spectra, IR spectra, X-ray crystallography details, topographical steric maps, additional photophysical data, solid angle analysis, plots of excited-state lifetime *vs.* other experimental observables. See DOI: https://doi.org/10.1039/d5sc09786j.
