# Supplementary material for: Steric augmentation of three-coordinate Cu(i) β-diketiminate isocyanide chromophores to achieve microsecond excited-state lifetime
Source: Chem Sci. 2026 Feb 24;17(15):7659–66. doi: 10.1039/d5sc09786j (PMC12931634; doi:10.1039/d5sc09786j)
Supplement: SC-017-D5SC09786J-s001 [file SC-017-D5SC09786J-s001.pdf]

Supplementary Information (SI) for Chemical Science.
This journal is © The Royal Society of Chemistry 2026

Supplementary Information for

Steric Augmentation of Three-Coordinate Cu(I) β-Diketiminate Isocyanide Chromophores to Achieve Microsecond Excited-State Lifetime

Ashish Kumar,[a] Dooyoung Kim,[a] Sean P. Dunphy,[b] Erin N. Lewis,[b]
Joshua S. Figueroa,[b] and Thomas S. Teets*[a]

[a] *University of Houston, Department of Chemistry, Houston, Texas 77204, United States.* E-mail: tteets@uh.edu

[b] *University of California, San Diego, La Jolla, California 92093, United States.*

Contents	Pages
Experimental section	S2–S5
NMR spectra	S6–S13
IR spectra and summary of C≡N stretching frequencies	S14–S27
X-ray crystallographic summary tables	S28–S35
Structural metrics from X-ray crystallography	S36
Topographical steric maps	S37–S40
Chemical structures of previously reported compounds described in this work	S41
Time-resolved photoluminescence decay traces	S41–S47
Photostability studies conducted during TCSPC measurements	S48–S51
Sample concentrations used for lifetime measurements	S51
Solid angle analysis and correlation with excited-state lifetime	S52
Excited-state lifetimes plotted vs. E^{ox} and triplet-state $E_{0,0}$	S53
Supplementary Information References	S54

Experimental Section

Materials. All reactions were executed in a nitrogen-filled glovebox operating at <1 ppm of O_2 and <1 ppm of H_2O. Solvents for reactions, UV–vis absorption, and electrochemical measurements were dried by the method of Grubbs,[1] passing through dual alumina columns on a commercial solvent purification system and stored over 3 Å molecular sieves. All NMR solvents were stored over 3 Å molecular sieves. Tetrabutylammonium hexafluorophosphate (NBu_4PF_6) was recrystallized from hot ethanol twice, and ferrocene was sublimed at reduced pressure before use in electrochemical measurements. All starting materials and reagents were obtained from commercial sources unless otherwise stated. Copper(I) *tert*-butoxide (CuO^{t}Bu),[2] β-diketiminate ligands PhNacNac(H),[3] CyNacNac(H),[4] 2,6-DmpNacNac(H),[5] DippNacNac(H),[5] and isocyanide ligands CN-Ar^{Ph2},[6] CN-Ar$^{(3,5\text{-dmp})2}$,[7] CN-Ar^{Mes2},[8] CN-Ar^{Tripp2},[8] and CN-Ar^{Dipp2},[8] were prepared according to previously reported procedures.

Physical Methods. ^{1}H and ^{13}C{^{1}H} NMR spectra (shown in Fig. S1–S16) were recorded at room temperature using a JEOL ECA-400 or JEOL ECA-500 NMR spectrometer. UV–vis absorption spectra were obtained in toluene solutions in screw-capped quartz cuvettes using an Agilent Cary 8454 UV–vis spectrophotometer. Steady-state photoluminescence and excitation spectra were recorded using a Horiba FluoroMax-4 spectrofluorometer. Stock solutions for both UV–vis absorption and emission spectra were prepared in a nitrogen-filled glovebox using dry and deoxygenated toluene. Samples for low-temperature emission were added to a custom quartz EPR tube with high-vacuum valve and immersed in a finger Dewar filled with liquid nitrogen. Photoluminescence quantum yields were determined relative to a standard of quinine sulfate in 0.05 M aqueous sulfuric acid, which has a reported fluorescence quantum yield (Φ_F) of 0.52[9] or tetraphenylporphyrin (TPP) in toluene, which has a reported fluorescence quantum yield (Φ_F) of 0.11.[10] Photoluminescence lifetimes were measured on a Horiba DeltaFlex Lifetime system, using 330 or 360 nm pulsed LED excitation. The LED sources are rated at a power of 1–2 pJ/pulse and a maximum pulse rate of 100 kHz was used, resulting in an average power below 0.2 μW for the duration of the measurement. Samples for lifetime measurements were prepared at a concentration of ca. 1–7 × 10^{-5} M; specific values for each complex are listed in Table S12. Cyclic voltammetry (CV) measurements were performed with a CH Instruments 602E potentiostat interfaced with a nitrogen-filled glovebox via wire feedthroughs. Samples were dissolved in THF with 0.1 M NBu_4PF_6 as a supporting electrolyte. A 3 mm diameter glassy carbon working electrode, a platinum wire counter electrode, and a silver wire pseudo-reference electrode immersed in 0.1 M NBu_4PF_6 THF solution were used. Potentials were referenced to an internal standard of ferrocene. Infrared (IR) spectra were recorded on a Thermo Scientific Nicolet Avatar 6700 FTIR spectrometer with a diamond ATR.

X-ray Crystallography Details. Single crystals were grown by slow evaporation of a pentane solution. Crystals were mounted on a Bruker Apex II three-circle diffractometer using MoKα radiation (λ=0.71073 Å). The data were collected at 123(2) K and processed and refined within the APEXII software. Structures were solved by intrinsic phasing in SHELXT and refined by standard difference Fourier techniques in the program SHELXL.[11] Hydrogen atoms were placed in calculated positions using the standard riding model and refined isotropically; all non-hydrogen atoms were refined anisotropically. Crystallographic details are summarized in Tables S1–S8.

Calculation of percent buried volume (%V_{bur}). Before calculating the percent buried volume (%$\underline{V}$bur), XYZ files of the copper complexes were generated from their CIF files. %V_{bur} values and corresponding topographical steric maps were obtained from the web-interfaced SambVca 2.1 program (https://www.aocdweb.com/OMtools/sambvca2.1/index.html), developed by Falivene et al.[12] Default parameters were used (Bondi radii scaled by 1.17, a 3.5 Å sphere radius from the copper center, and 0.1 Å of the mesh spacing for the numerical integration). Hydrogen atoms were excluded from the calculation.

General procedure for preparation of Cu(I) complexes. In a glovebox, a mixture of CuO^{t}Bu and 1 equivalent of the respective protonated RNacNac(H) proligand in 2 mL of toluene was stirred at room temperature for 1 hour. In a

separate container ca. 0.6 equivalent of the respective isocyanide ligand was dissolved in 2 mL of toluene. The solution of the isocyanide ligand was added to the mixture of CuO^{t}Bu and RNacNac(H) solution via pipet. The resulting mixture was stirred for another 24–72 h at room temperature or at 60° C. The solution was filtered through a pad of glass fiber, and all volatiles were evaporated under vacuum to reduce the volume to ≤ 1 mL. Two different methods for isolating and purifying the product were used, depending on the solubility of the target compound. *Procedure 1*: Approximately 1 mL of pentane was added to the residue, and the mixture was kept in the freezer (−30 °C) overnight to precipitate the solid product. The resulting product was washed with pentane and dried under vacuum. *Procedure 2*: Approximately 1 mL of pentane was added to the residue, and the mixture was kept in the freezer (−30 °C) overnight to precipitate the solid. The resulting solid was washed with pentane and kept in the freezer (−30 °C). After a second round of extraction with pentane, the pure product precipitated from the pentane extracts.

Preparation of Cu(PhNacNac)(CN-Ar^{Ph2}) (Ph-Ar^{Ph2}). Prepared by the general procedure using CuO^{t}Bu (41 mg, 0.30 mmol), PhNacNac(H) (75 mg, 0.30 mmol), and *m*-terphenyl isocyanide (CN-Ar^{Ph2}, 51 mg, 0.20 mmol). The mixture was stirred at room temperature for 24 h and the product was purified via procedure 1. Yield: 97 mg (85%). ^{1}H NMR (400 MHz, C_6D_6, ppm): δ = 7.15–7.05 (m, 13H, Ar*H*, overlapped with residual C_6D_5H solvent peak), 6.93–6.79 (m, 10H, Ar*H*), 4.92 (s, 1H, PhN–C(CH_3)=C*H*–C(CH_3)=NPh), 1.93 (s, 6H, C*H*$_3$). ^{13}C{^{1}H} NMR (126 MHz, C_6D_6, ppm): δ = 162.3, 155.6, 139.8 (br), 137.4 (br), 129.5, 129.3, 128.9, 128.6, 128.5, 128.4, 124.7, 122.4, 97.0, 23.2. The broad isocyanide (*C*≡N) ^{13}C resonance was not located. IR (Neat, cm^{-1}): ṽ = 2117 (s). UV–vis (toluene, nm (M^{-1}cm^{-1})): λ_{max} (ε) = 363 (30000).

Preparation of Cu(CyNacNac)(CN-Ar^{Ph2}) (Cy-Ar^{Ph2}). Prepared by the general using CuO^{t}Bu (41 mg, 0.30 mmol), CyNacNac(H) (79 mg, 0.30 mmol), and *m*-terphenyl isocyanide (CN-Ar^{Ph2}, 51 mg, 0.20 mmol). The mixture was stirred at room temperature for 48 h and the product was purified via procedure 2. Yield: 20 mg (17%). ^{1}H NMR (400 MHz, C_6D_6, ppm): δ = 7.46–7.43 (m, 4H, Ar*H*), 7.24 – 7.19 (m, 4H, Ar*H),* 7.14–7.10 (m, 2H, Ar*H*), 7.03–7.00 (m, 2H, Ar*H*), 6.97–6.91 (m, 1H, Ar*H*), 4.54 (s, 1H, CyN–C(CH_3)=C*H*–C(CH_3)=NCy), 3.45 (td, *J* = 10.6, 5.1 Hz, 2H, C*H*), 2.02 (s, 6H, C*H*$_3$), 1.78 – 1.71 (m, 4H, C*H*$_2$), 1.63 (d, *J* = 13.1 Hz, 4H, C*H*$_2$), 1.54–1.41 (m, 6H, C*H*$_2$), 1.29–1.21 (m, 6H, C*H*$_2$). Some of the aliphatic peaks overlapped with the pentane peaks as the product was obtained by precipitation from pentane. ^{13}C{^{1}H} NMR (126 MHz, C_6D_6, ppm): δ = 160.5, 139.3, 138.0, 130.0, 129.3, 128.9, 128.6, 128.4, 124.7 (br), 94.9, 60.3, 38.0, 26.5, 22.5. IR (Neat, cm^{-1}): ṽ = 2102 (s) (C≡N). UV–vis (toluene, nm (M^{-1}cm^{-1})): λ_{max} (ε) = 337 (4400), 351 (4600), 400 (1200).

Preparation of Cu(2,6-DmpNacNac)(CN-Ar^{Ph2}) (2,6-Dmp-Ar^{Ph2}). Prepared by the general procedure using CuO^{t}Bu (41 mg, 0.30 mmol), 2,6-DmpNacNac(H) (92 mg, 0.30 mmol), and *m*-terphenyl isocyanide (51 mg, 0.20 mmol). The mixture was stirred at room temperature for 24 h and the product was purified via procedure 1. Yield: 47 mg (38%). ^{1}H NMR (400 MHz, C_6D_6, ppm): δ = 7.24–7.16 (m, 6H, Ar*H*), 7.04 (d, *J* = 7.4 Hz, 4H, Ar*H*), 7.01–6.96 (m, 4H, Ar*H*), 6.89 (t, *J* = 7.4 Hz, 2H, Ar*H*), 6.77–6.69 (m, 3H, Ar*H*), 4.92 (s, 1H, 2,6-DmpN–C(CH_3)=C*H*–C(CH_3)=N-2,6-Dmp), 2.20 (s, 12H, ArC*H*$_3$), 1.68 (s, 6 H, DmpN–C(C*H*$_3$)=CH–C(C*H*$_3$)=NDmp). ^{13}C{^{1}H} NMR (126 MHz, C_6D_6, ppm): δ = 162.4, 152.9, 139.2, 137.6, 130.2, 129.4, 129.1, 128.7, 128.4, 128.2, 123.7 (br), 122.7, 94.2, 22.3, 19.3. One missing aromatic peak presumably overlapped with the C_6D_5H solvent peak. IR (Neat, cm^{-1}): ṽ = 2117 (s) (C≡N). UV–vis (toluene, nm (M^{-1}cm^{-1})): λ_{max} (ε) = 343 (46000), 394(sh) (11000).

Preparation of Cu(DippNacNac)(CN-Ar^{Ph2}) (Dipp-Ar^{Ph2}). Prepared by the general procedure using CuO*t*Bu (41 mg, 0.30 mmol), DippNacNac(H) (126 mg, 0.30 mmol), and *m*-terphenyl isocyanide (51 mg, 0.20 mmol). The mixture was stirred at room temperature for 24 h and the product was purified via procedure 2. Yield: 16 mg (11%). ^{1}H NMR (400 MHz, C_6D_6, ppm): δ = 7.15–7.11 (m, 10H, Ar*H*, overlapped with C_6D_5H residual solvent peak), 7.10–7.05 (m, 2H, Ar*H*), 7.00–6.95 (m, 4H, Ar*H*), 6.70 (s, 3H, Ar*H*), 4.98 (s, 1H, DippN–C(CH_3)=C*H*–C(CH_3)=NDipp), 3.41 (sept, *J* = 6.9 Hz, 4H, –C*H*(CH_3)$_2$), 1.73 (s, 6 H, DippN–C(C*H*$_3$)=CH–C(C*H*$_3$)=NDipp), 1.24 (d, *J* = 7.0 Hz, 12H, –CH(C*H*$_3$)$_2$), 1.09 (d, *J* = 6.8 Hz,

12H, –CH(CH_3)$_2$). $^{13}C\{^1H\}$ NMR (126 MHz, C_6D_6, ppm): δ = 163.5, 150.0, 142.8, 141.3, 140.6, 139.6, 137.8, 130.2, 128.93, 128.89, 125.9, 124.0, 123.6, 123.2, 94.6, 28.1, 24.8, 23.45, 23.39. IR (Neat, cm^{-1}): ṽ = 2113 (s) (C≡N). UV–vis (toluene, nm ($M^{-1}cm^{-1}$)): λ_{max} (ε) = 343 (40000), 394(sh) (9100).

Preparation of Cu(DippNacNac)(CN-Ar$^{(3,5\text{-}Dmp)2}$) (Dipp-Ar$^{(3,5\text{-}Dmp)2}$). Prepared by the general procedure using CuO*t*Bu (41 mg, 0.30 mmol), DippNacNac(H) (79 mg, 0.30 mmol), and 2,6-bis(3,5-dimethylphenyl)phenyl isocyanide (51 mg, 0.20 mmol). The mixture was stirred at 60 °C for 48 h, and the product was purified via procedure 1. Yield: 46 mg (29%). ^{1}H NMR (400 MHz, C_6D_6, ppm): δ = 7.11–7.04 (m, 6H, Ar*H*), 6.82 (s, 2H, Ar*H*), 6.80–6.77 (m, 2H, Ar*H*), 6.74–6.71 (m, 5H, Ar*H*), 4.93 (s, 1H, DippN–C(CH_3)=C*H*–C(CH_3)=NDipp), 3.39 (sept, *J* = 6.9 Hz, 1H, –C*H*(CH_3)$_2$), 2.21 (s, 12H, Ar–CH_3), 1.73 (s, 6H, DippN–C(CH_3)=CH–C(CH_3)=NDipp), 1.25 (d, *J* = 6.9 Hz, 12H, –CH(CH_3)$_2$), 1.06 (d, *J* = 6.8 Hz, 12H, –CH(CH_3)$_2$). $^{13}C\{^1H\}$ NMR (126 MHz, C_6D_6, ppm): δ = 163.2, 149.7, 140.4, 140.3, 138.2, 137.9, 130.4, 130.1, 128.4, 126.8, 123.7, 123.6, 123.1, 94.5, 28.0, 24.6, 23.5, 23.3, 21.5. The broad isocyanide (*C*≡N) ^{13}C resonance was not located. IR (Neat, cm^{-1}): ṽ = 2113 (s) (C≡N). UV–vis (toluene, nm ($M^{-1}cm^{-1}$)): λ_{max} (ε) = 348 (49000), 399(sh) (11000).

Preparation of Cu(DippNacNac)(CN-Ar^{Mes2}) (Dipp-Ar^{Mes2}). Prepared by the general procedure using CuO*t*Bu (34 mg, 0.25 mmol), DippNacNac(H) (104 mg, 0.30 mmol), and 2,6-dimesitylphenyl isocyanide (50 mg, 0.15 mmol). The mixture was stirred at room temperature for 72 h and the product was purified by procedure 1. Yield: 92 mg (75%). ^{1}H NMR (400 MHz, C_6D_6, ppm): δ = 7.13 (s, 6H, Ar*H*), 6.82–6.76 (m, 5H, Ar*H*), 6.58 (d, *J* = 7.6 Hz, 2H, Ar*H*), 4.91 (s, 1H, DippN–C(CH_3)=C*H*–C(CH_3)=NDipp), 3.28 (sept, *J* = 7.1 Hz, 4H, –C*H*(CH_3)$_2$), 2.30 (s, 6H, Ar–CH_3), 1.77 (s, 12H, Ar–CH_3), 1.76 (s, 6H, DippN–C(CH_3)=CH–C(CH_3)=NDipp), 1.25 (d, *J* = 7.0 Hz, 12H, –CH(CH_3)$_2$), 1.03 (d, *J* = 6.8 Hz, 12H, –CH(CH_3)$_2$). $^{13}C\{^1H\}$ NMR (126 MHz, C_6D_6, ppm): δ = 162.7, 149.9, 140.0, 139.1, 137.1, 135.4, 134.2, 129.9, 129.2, 128.9, 128.4 126.3 (br), 123.5, 123.1, 93.9, 28.1, 24.1, 23.3, 23.0, 21.4, 19.9. IR (Neat, cm^{-1}): ṽ = 2117 (s) (C≡N). UV–vis (toluene, nm ($M^{-1}cm^{-1}$)): λ_{max} (ε) = 347 (24000).

Preparation of Cu(DippNacNac)(CN-Ar^{Tripp2}) (Dipp-Ar^{Tripp2}). Prepared by the general procedure using CuO*t*Bu (22 mg, 0.16 mmol), DippNacNac(H) (67 mg, 0.16 mmol), and 2,6-bis(2,4,6-triisopropylphenyl)phenyl isocyanide (50 mg, 0.098 mmol). The mixture was stirred at room temperature for 72 h, and the product was isolated and purified by procedure 1. Yield: 86 mg (89%). ^{1}H NMR (400 MHz, C_6D_6, ppm): δ = 7.13–7.07 (m, 10H, Ar*H*), 6.63–6.59 (m, 1 H, Ar*H*), 6.55–6.53 (m, 2H, Ar*H*), 4.93 (s, 1H, DippN–C(CH_3)=C*H*–C(CH_3)=N), 3.24 (sept, *J* = 7.0 Hz, 4H, –C*H*(CH_3)$_2$, 3.02 (sept, *J* = 6.9 Hz, 2H, –C*H*(CH_3)$_2$), 2.50 (sept, *J* = 7.0 Hz, 4H, –C*H*(CH_3)$_2$), 1.65 (s, 6H, DippN–C(CH_3)=CH–C(CH_3)=NDipp), 1.43 (d, *J* = 6.9 Hz, 12H, –CH(CH_3)$_2$), 1.23 (d, *J* = 6.9 Hz, 12H, –CH(CH_3)$_2$), 1.08 (d, *J* = 6.9 Hz, 12H, –CH(CH_3)$_2$), 1.03 (d, *J* = 6.8 Hz, 12H,–CH(CH_3)$_2$), 0.95 (d, *J* = 6.9 Hz, 12H, ,–CH(CH_3)$_2$). $^{13}C\{^1H\}$ NMR (126 MHz, C_6D_6, ppm): δ = 163.5, 151.0, 148.5, 146.3, 140.1, 139.6, 133.2, 131.6, 128.4, 123.8, 123.5, 121.8, 94.2, 34.4, 31.5, 28.4, 24.8, 24.7, 24.2, 24.1, 23.7, 23.0. One missing aromatic peak presumably overlapped with the C_6D_5H solvent peak, and the broad isocyanide (*C*≡N) ^{13}C resonance was not located. IR (Neat, cm^{-1}): ṽ = 2102 (s) (C≡N). UV–vis (toluene, nm ($M^{-1}cm^{-1}$)): λ_{max} (ε) = 348 (31000).

Preparation of Cu(DippNacNac)(CN-Ar^{Dipp2}) (Dipp-Ar^{Dipp2}). Prepared by the general procedure using CuO*t*Bu (41 mg, 0.2 mmol), DippNacNac(H) (79 mg, 0.2 mmol), and 2,6-bis(2,6-diisopropylphenyl)phenyl isocyanide (50 mg, 0.12 mmol). The mixture was stirred at room temperature for 72 h and purified by procedure 1. Yield: 15 mg (14%). ^{1}H NMR (400 MHz, C_6D_6, ppm): δ = 7.28 (t, *J* = 7.8 Hz, 2H, Ar*H*), 7.14–7.01 (m, 10H, Ar*H*), 6.72 (dd, *J* = 8.4, 6.6 Hz, 1H, Ar*H*), 6.65–6.61 (m, 2H, Ar*H*), 4.91 (s, 1H, DippN–C(CH_3)=C*H*–C(CH_3)=NDipp), 3.20 (sept, *J* = 7.0 Hz,, 4 H, –C*H*(CH_3)$_2$), 2.43 (sept, *J* = 6.8 Hz, 4H, –C*H*(CH_3)$_2$), 1.65 (s, 6H, DippN–C(CH_3)=CH–C(CH_3)=NDipp), 1.23 (d, *J* = 6.9 Hz, 12H, –CH(CH_3)$_2$), 1.08 (d, *J* = 6.8 Hz, 12 H,–CH(CH_3)$_2$), 0.99 (d, *J* = 6.9 Hz, 12 H, –CH(CH_3)$_2$), 0.91 (d, J = 7.0 Hz, 12 H, –CH(CH_3)$_2$). $^{13}C\{^1H\}$ NMR (126 MHz, C_6D_6, ppm): δ = 163.4, 151.1, 146.1, 139.8, 139.6, 135.0, 131.1, 129.6, 129.3, 128.4, 123.9, 123.8, 123.6, 93.72, 31.01, 28.11, 24.55, 24.19, 23.84, 22.80, 21.13. The broad isocyanide (*C*≡N) ^{13}C

resonance was not located. IR (Neat, cm^{-1}): $\tilde{\nu}$ = 2105 (s) (C≡N). UV–vis (toluene, nm ($M^{-1}cm^{-1}$)): λ_{max} (ε) = 347 (37000), 391(sh) (8200), 420(sh) (4500).

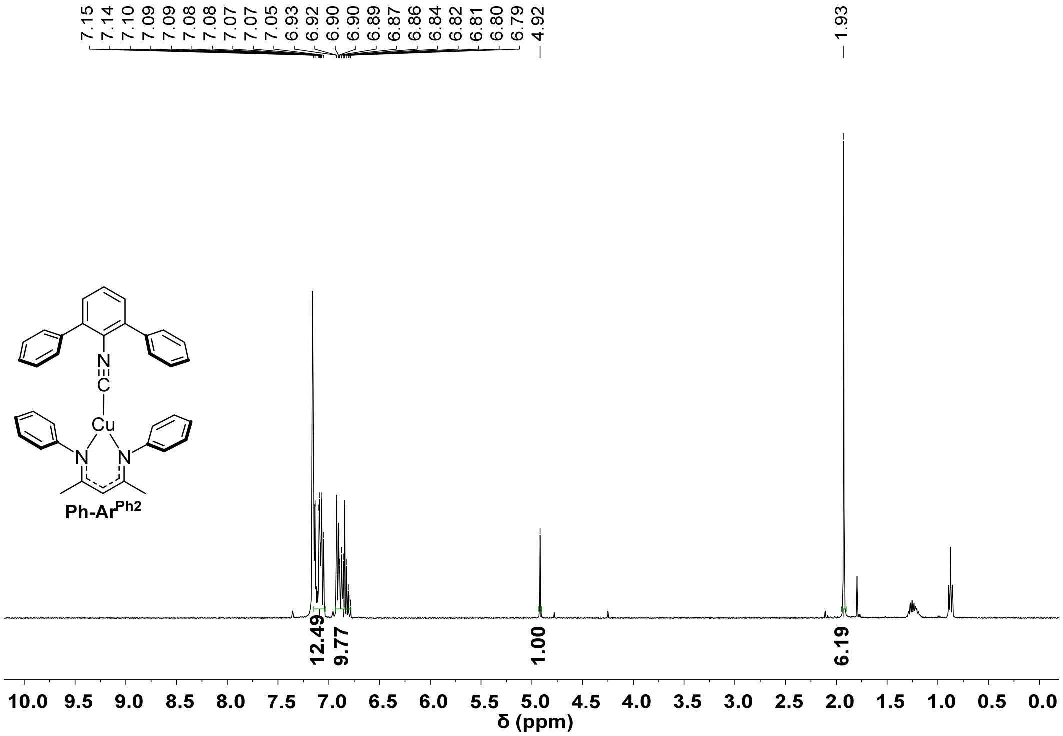

Fig. S1. ^{1}H NMR spectrum of **Ph-Ar^{Ph2}**, recorded at 400 MHz in C_6D_6.

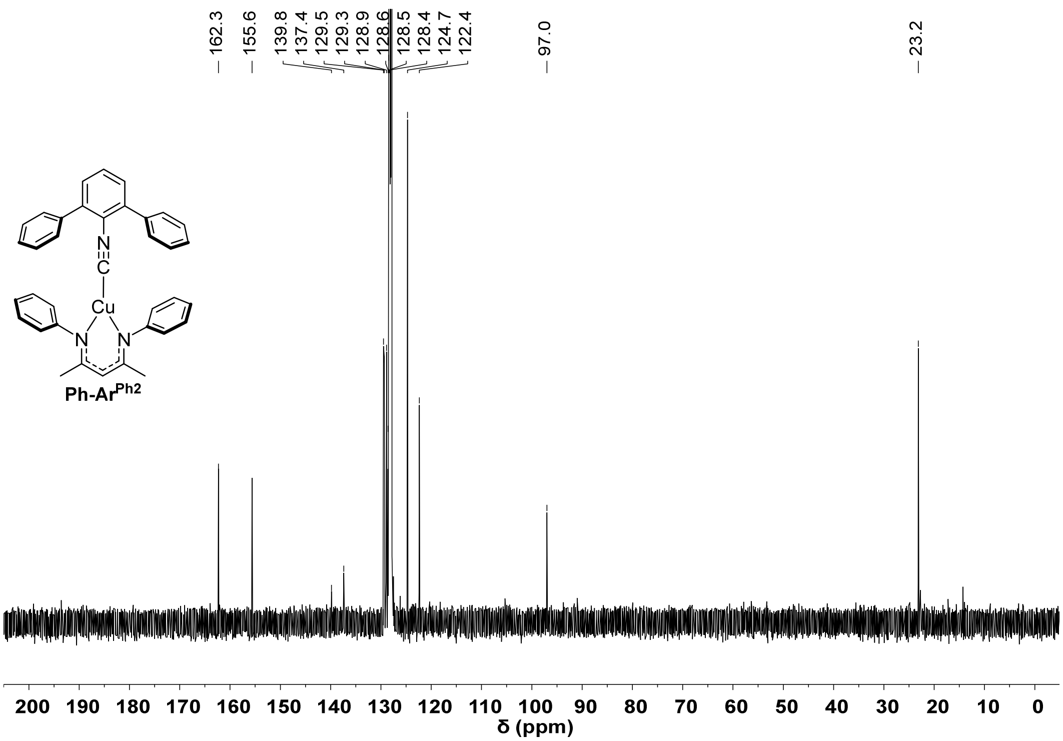

Fig. S2. ^{13}C{^{1}H} NMR spectrum of **Ph-Ar^{Ph2}**, recorded at 126 MHz in C_6D_6.

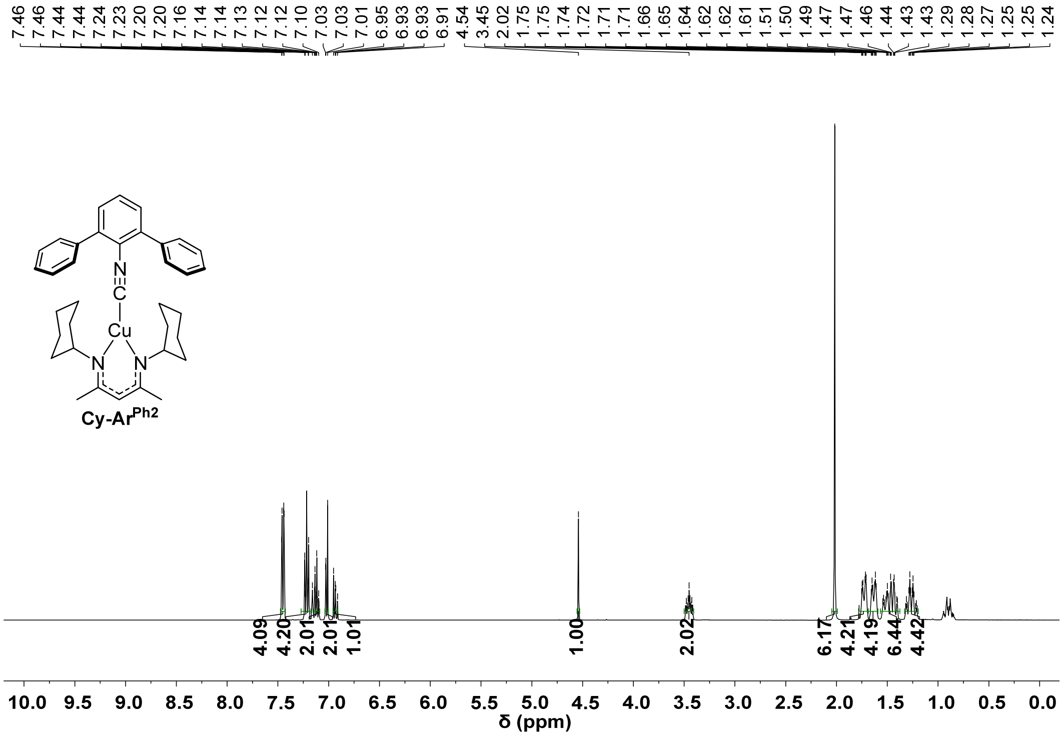

Fig. S3. ^{1}H NMR spectrum of **Cy-Ar^{Ph2}**, recorded at 400 MHz in C_6D_6.

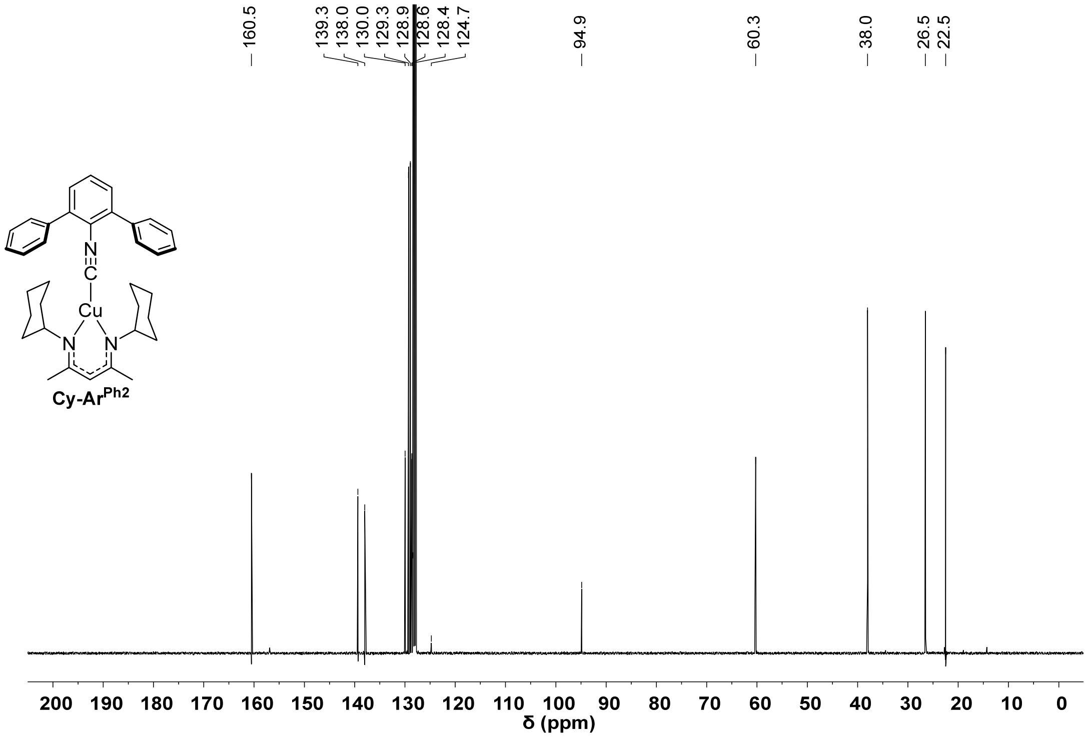

Fig. S4. ^{13}C{^{1}H} NMR spectrum of **Cy-Ar^{Ph2}**, recorded at 126 MHz in C_6D_6.

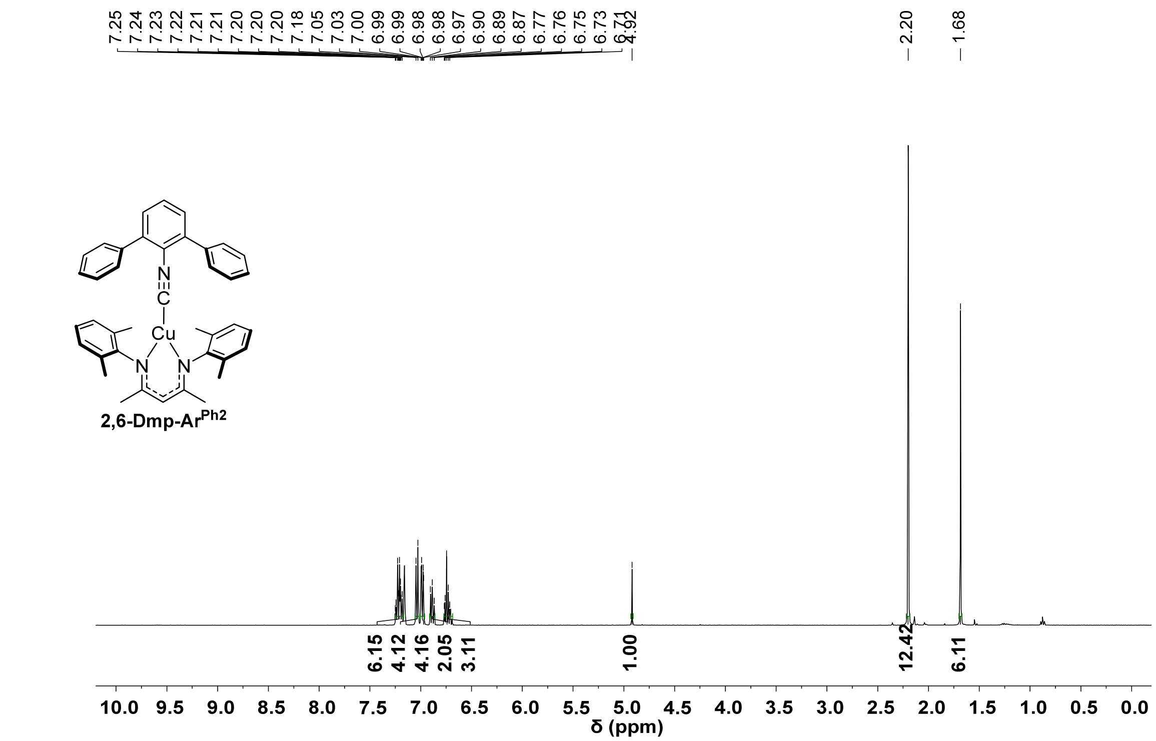

Fig. S5. ^{1}H NMR spectrum of **2,6-Dmp-Ar$^{\mathbf{Ph2}}$**, recorded at 400 MHz in C_6D_6.

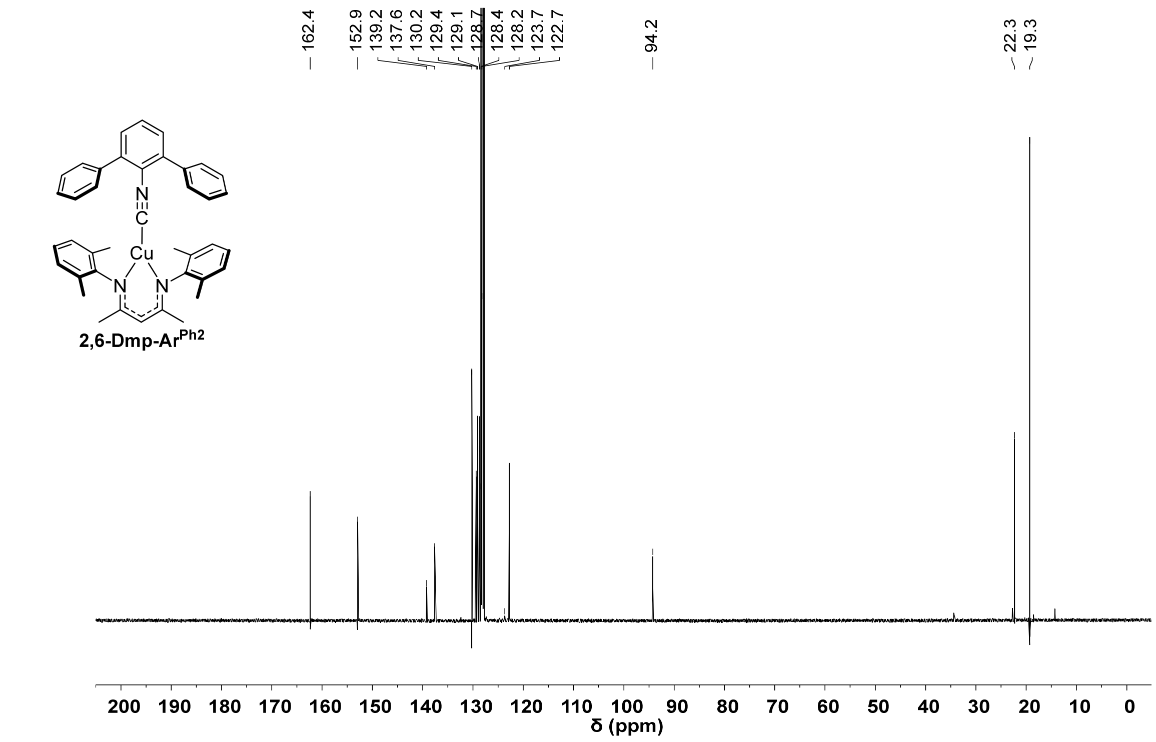

Fig. S6. $^{13}C\{^{1}H\}$ NMR spectrum of **2,6-Dmp-Ar$^{\mathbf{Ph2}}$**, recorded at 126 MHz in C_6D_6.

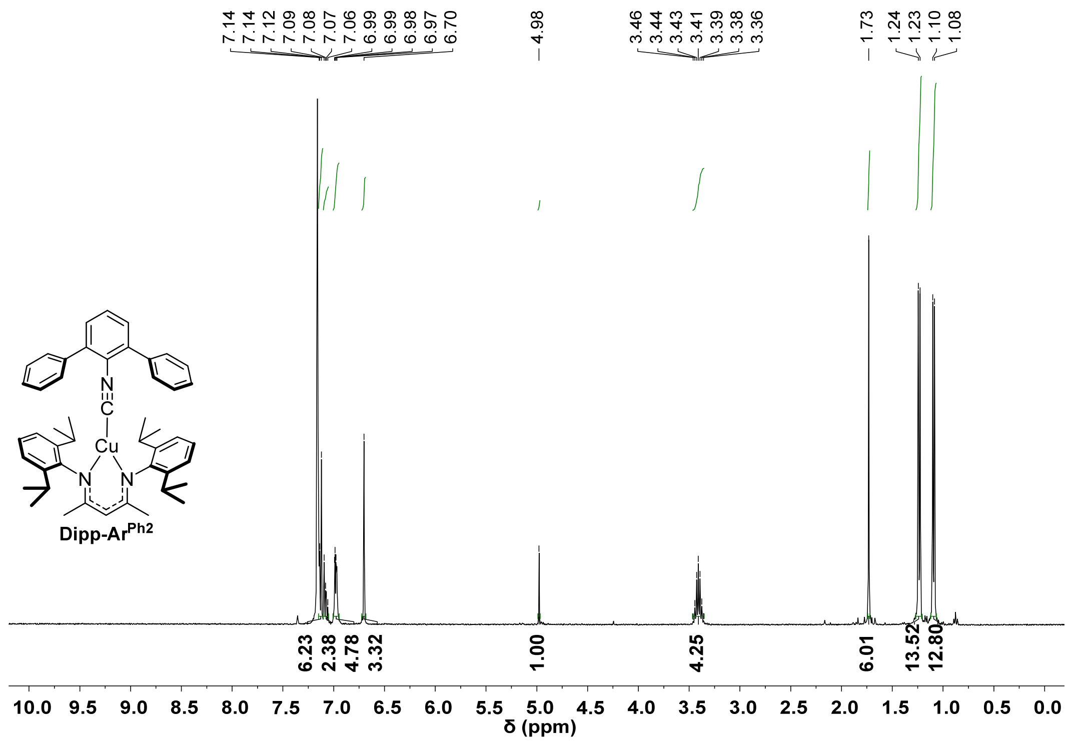

Fig. S7. ^{1}H NMR spectrum of **Dipp-Ar^{Ph2}**, recorded at 400 MHz in C_6D_6.

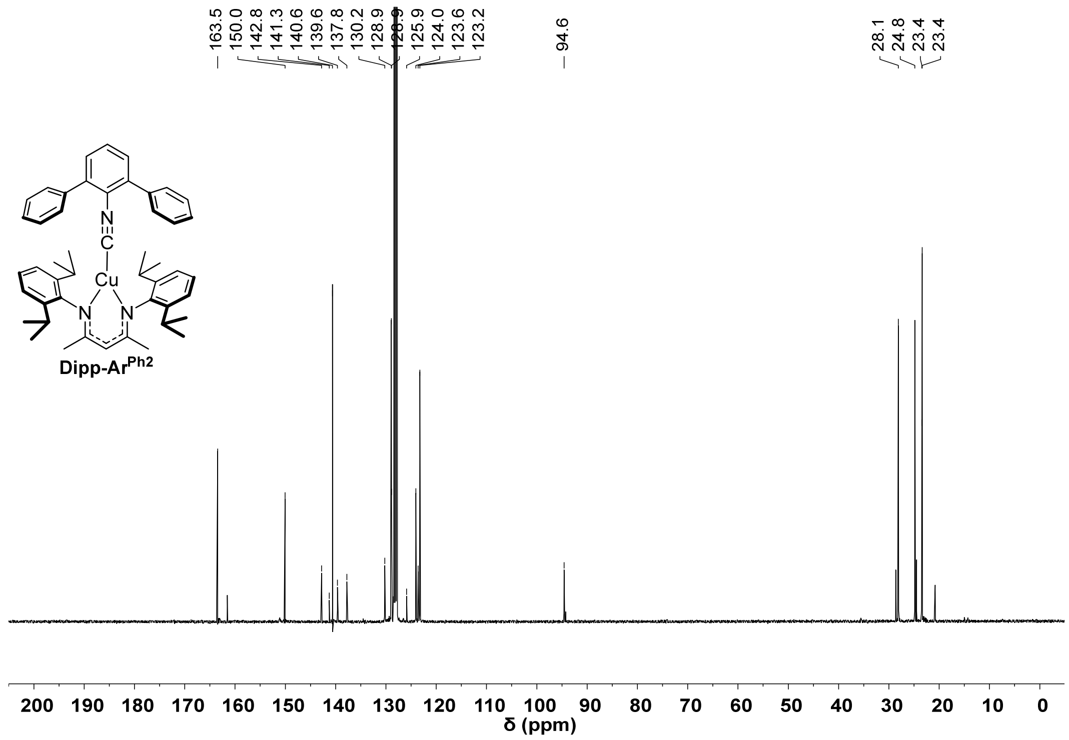

Fig. S8. $^{13}C\{^{1}H\}$ NMR spectrum of **Dipp-Ar^{Ph2}**, recorded at 126 MHz in C_6D_6.

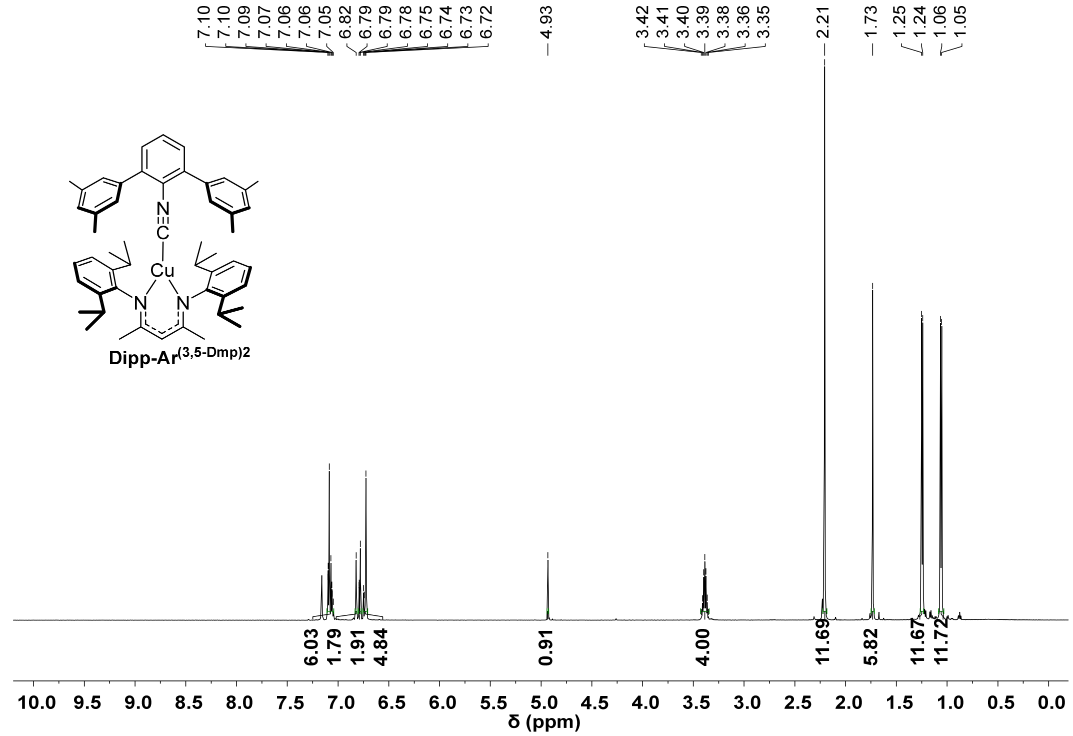

Fig. S9. ^{1}H NMR spectrum of **Dipp-Ar$^{(3,5\text{-Dmp})2}$**, recorded at 400 MHz in C_6D_6.

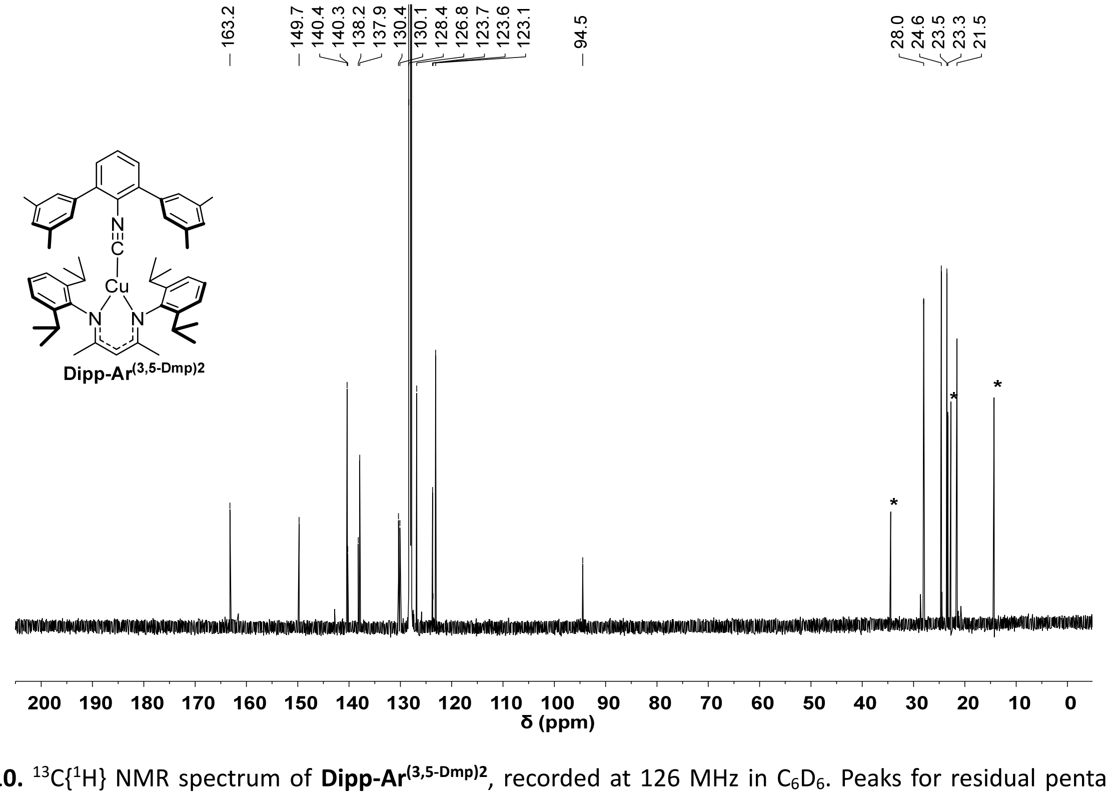

Fig. S10. $^{13}C\{^{1}H\}$ NMR spectrum of **Dipp-Ar$^{(3,5\text{-Dmp})2}$**, recorded at 126 MHz in C_6D_6. Peaks for residual pentane are marked with asterisks (*).

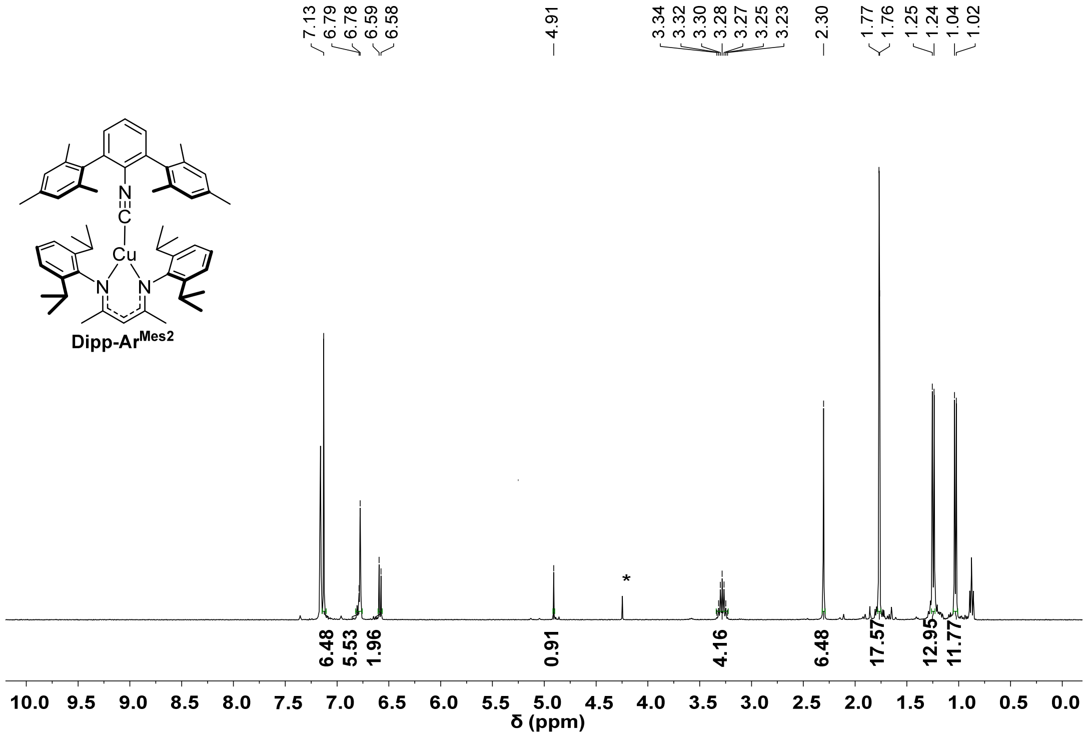

Fig. S11. ^{1}H NMR spectrum of **Dipp-Ar^{Mes2}**, recorded at 400 MHz in C_6D_6. A peak attributed to CH_2Cl_2 contaminant from the NMR solvent is marked with an asterisk (*).

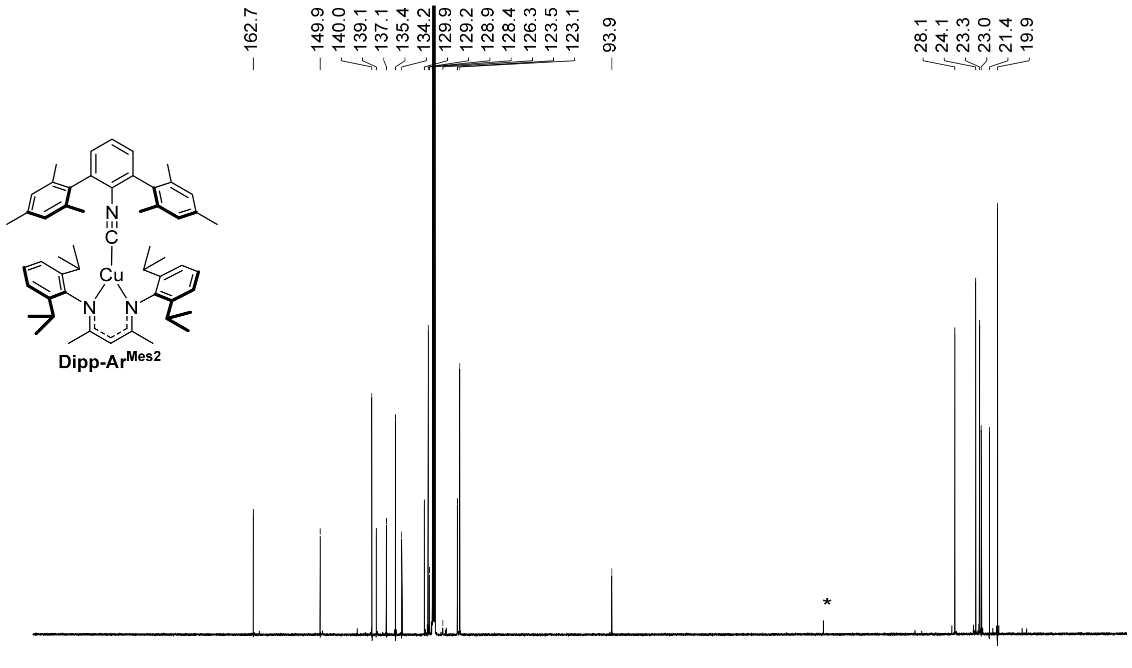

Fig. S12. $^{13}C\{^{1}H\}$ NMR spectrum of **Dipp-Ar^{Mes2}**, recorded at 126 MHz in C_6D_6. A peak attributed to CH_2Cl_2 contaminant from the NMR solvent is marked with an asterisk (*).

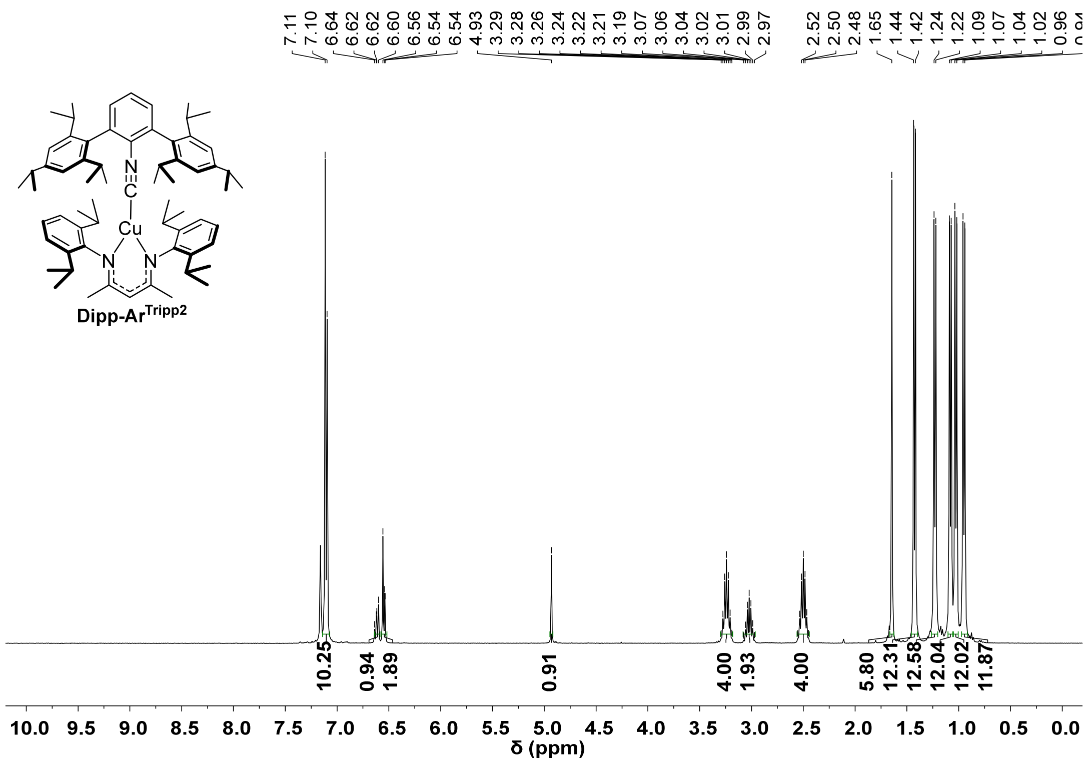

Fig. S13. ^{1}H NMR spectrum of **Dipp-Ar$^{\mathbf{Tripp2}}$**, recorded at 400 MHz in C_6D_6.

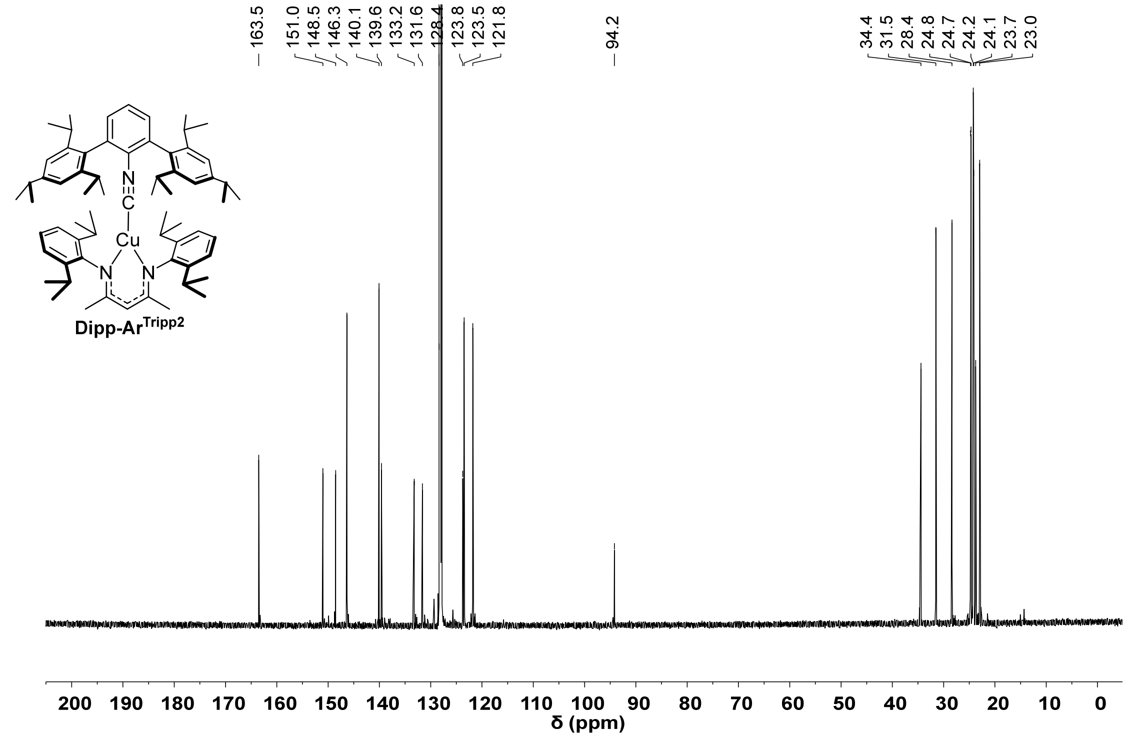

Fig. S14. $^{13}C\{^1H\}$ NMR spectrum of **Dipp-Ar$^{\mathbf{Tripp2}}$**, recorded at 126 MHz in C_6D_6.

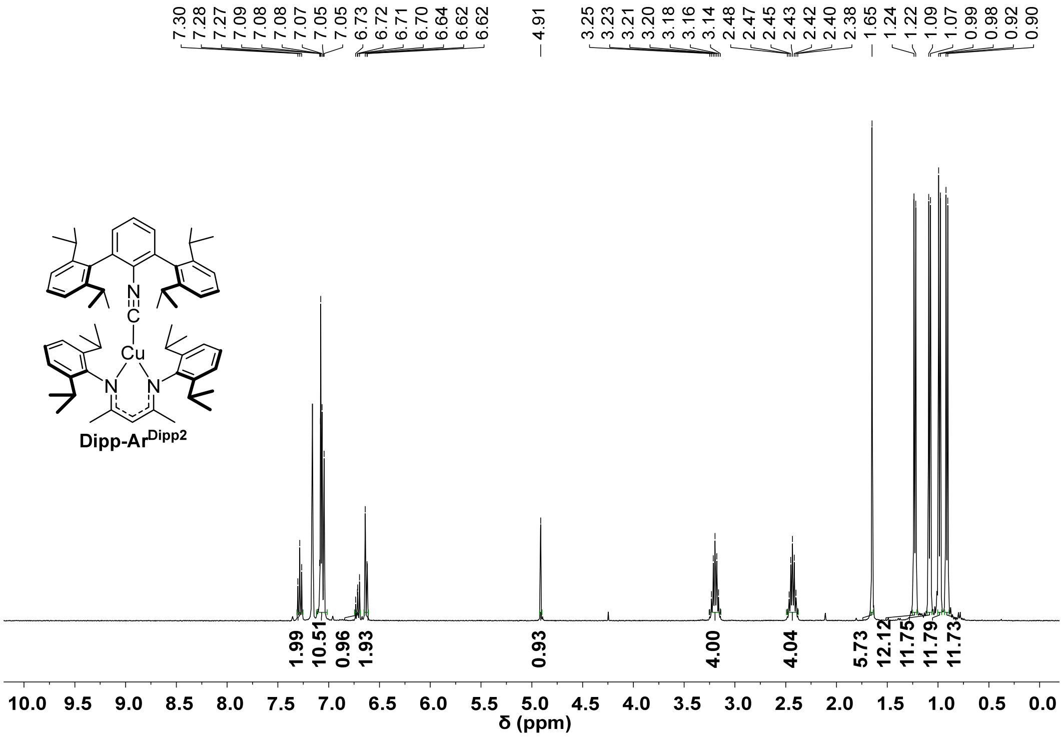

Fig. S15. ^{1}H NMR spectrum of **Dipp-Ar$^{\mathbf{Dipp2}}$**, recorded at 400 MHz in C_6D_6.

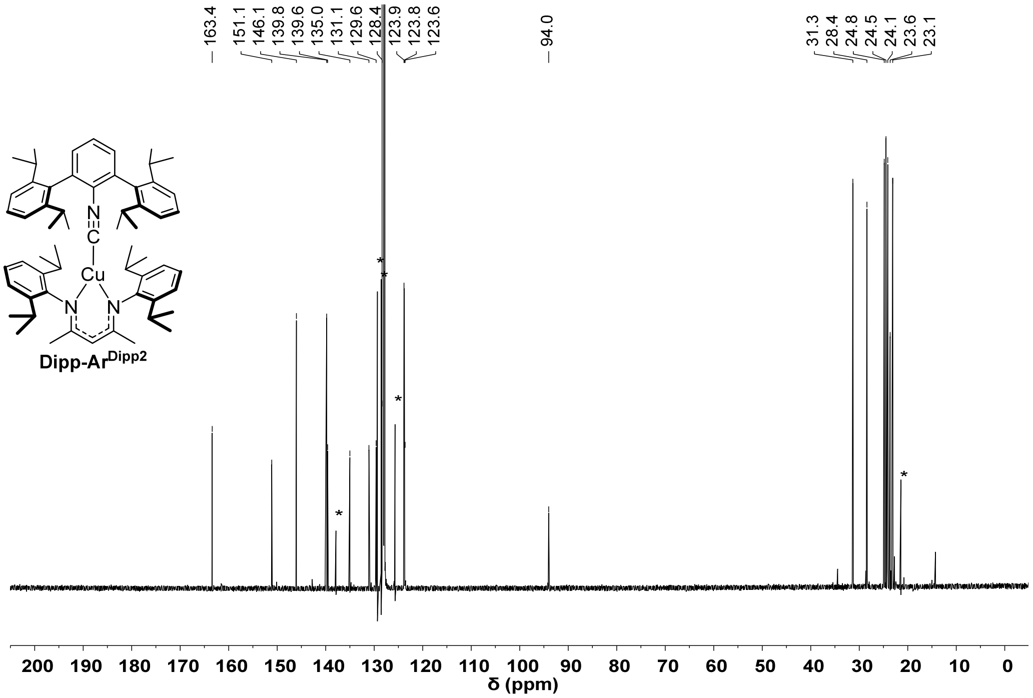

Fig. S16. ^{13}C{^{1}H} NMR spectrum of **Dipp-Ar$^{\mathbf{Dipp2}}$**, recorded at 126 MHz in C_6D_6. Peaks for residual toluene are marked with asterisks (*).

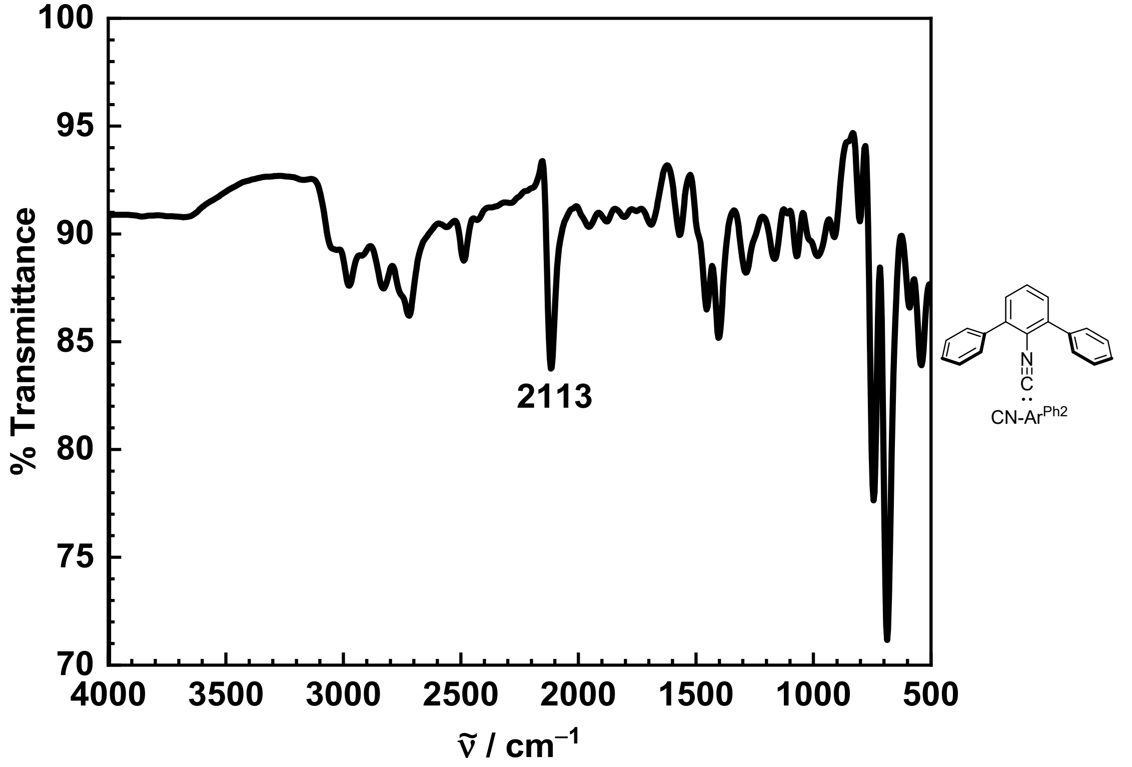

Fig. S17. FTIR spectrum of CN-Ar^{Ph2}, recorded on a neat sample. The C≡N stretching frequency ($\tilde{\nu}_{CN}$), in wavenumbers, is labeled.

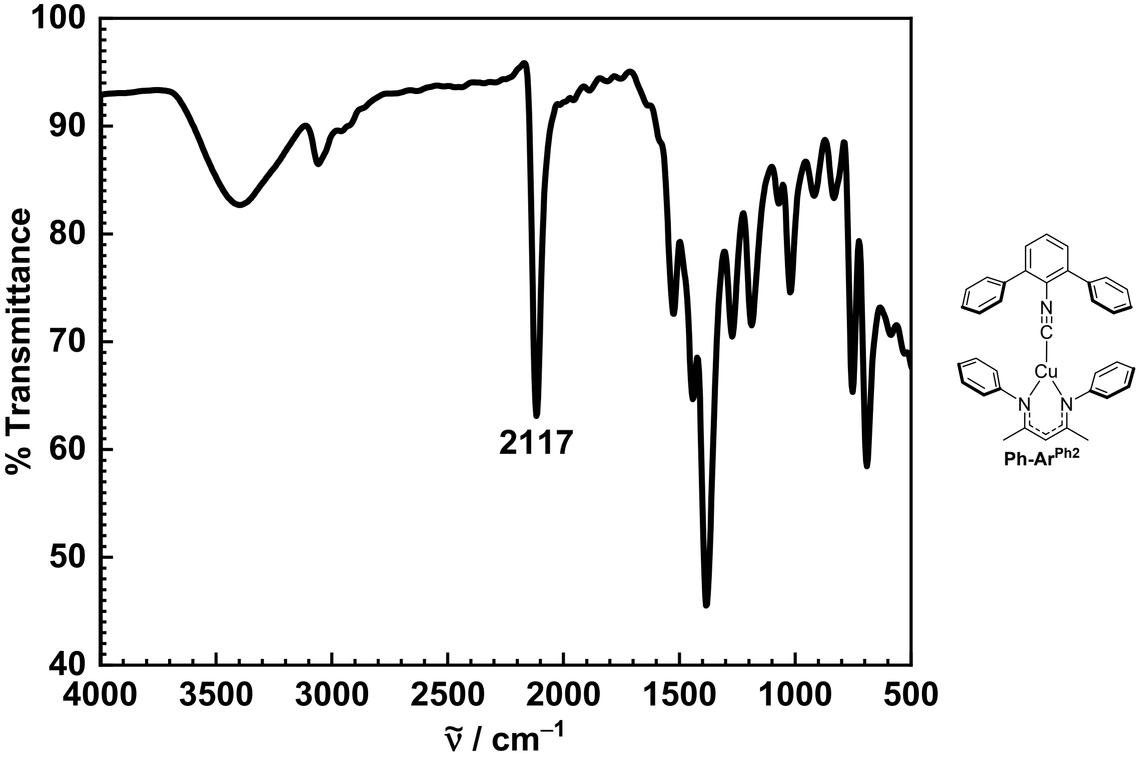

Fig. S18. FTIR spectrum of **Ph-Ar^{Ph2}**, recorded on a neat sample. The C≡N stretching frequency ($\tilde{\nu}_{CN}$), in wavenumbers, is labeled.

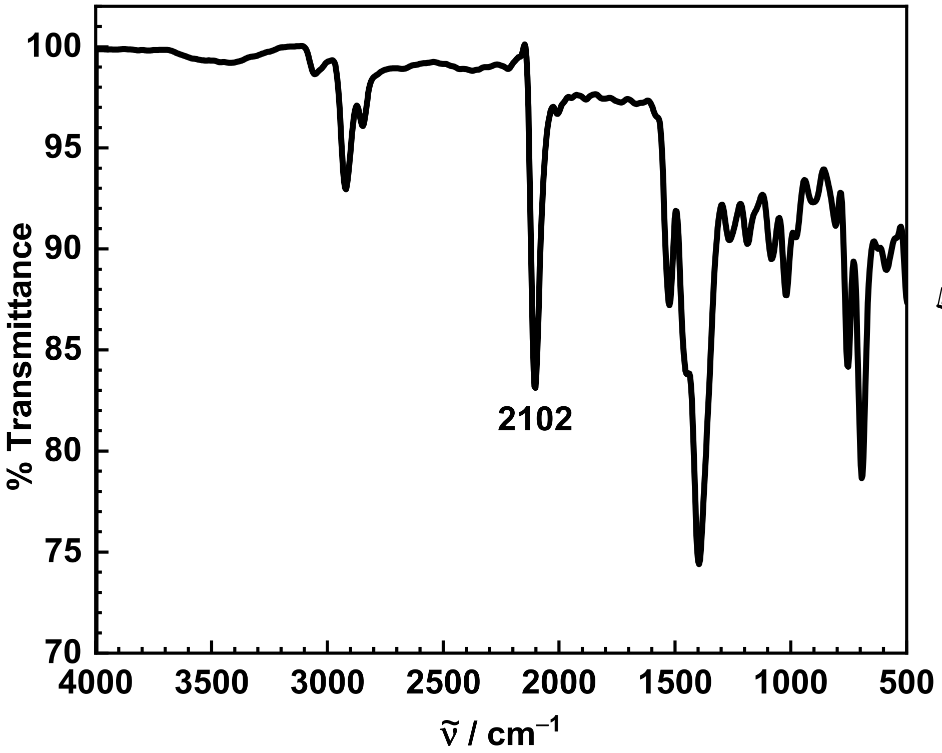

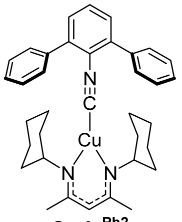

Cy-Ar$^{\mathbf{Ph2}}$

Fig. S19. FTIR spectrum of **Cy-Ar**$^{\mathbf{Ph2}}$, recorded on a neat sample. The C≡N stretching frequency ($\tilde{\nu}_{CN}$), in wavenumbers, is labeled.

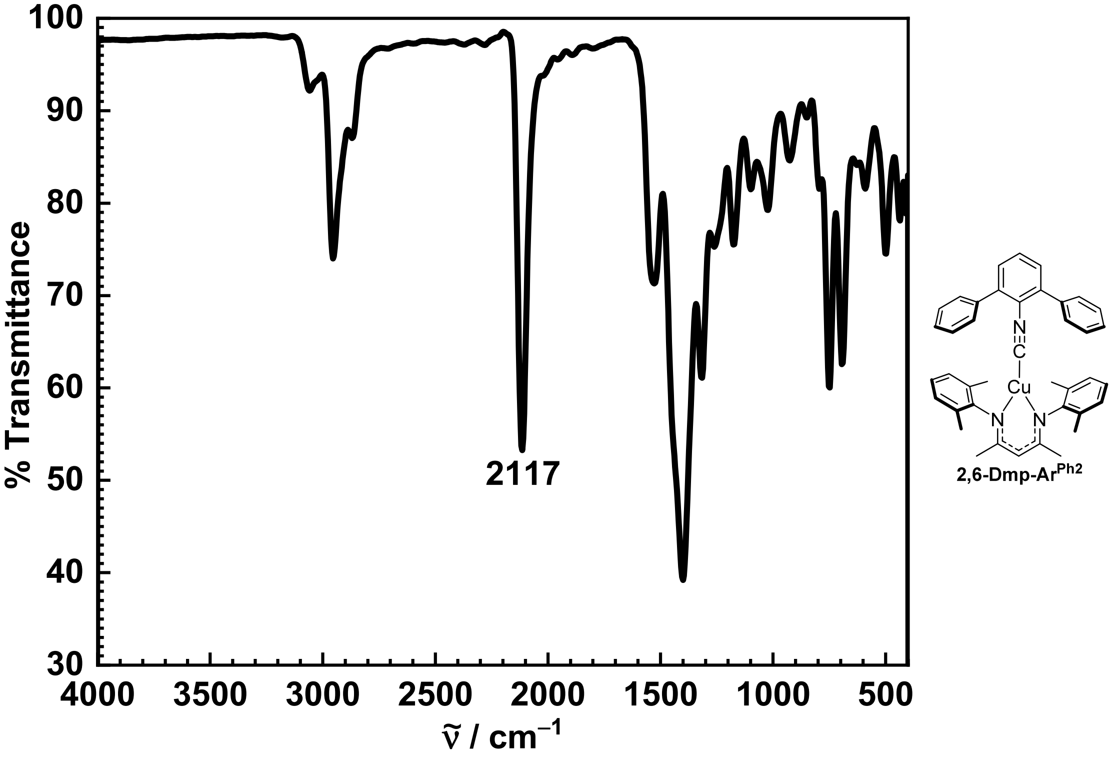

Fig. S20. FTIR spectrum of **2,6-Dmp-Ar^{Ph2}**, recorded on a neat sample. The C≡N stretching frequency ($\tilde{\nu}_{CN}$), in wavenumbers, is labeled.

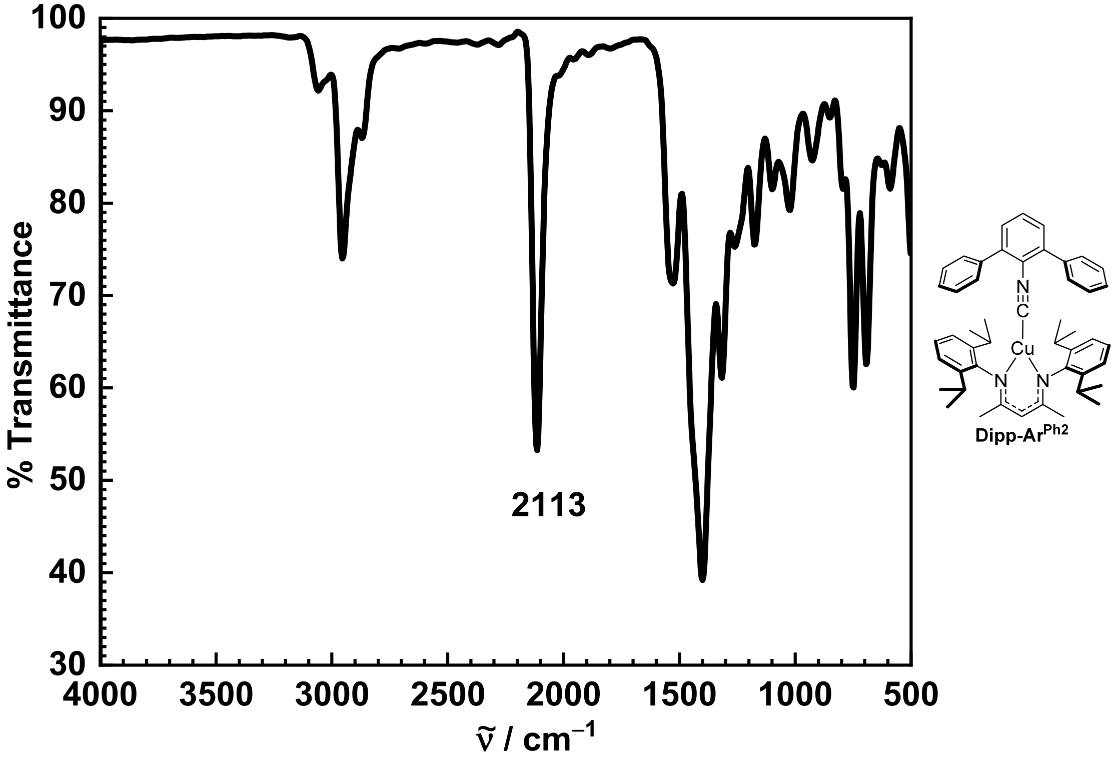

Fig. S21. FTIR spectrum of **Dipp-Ar^{Ph2}**, recorded on a neat sample. The C≡N stretching frequency ($\tilde{\nu}_{CN}$), in wavenumbers, is labeled.

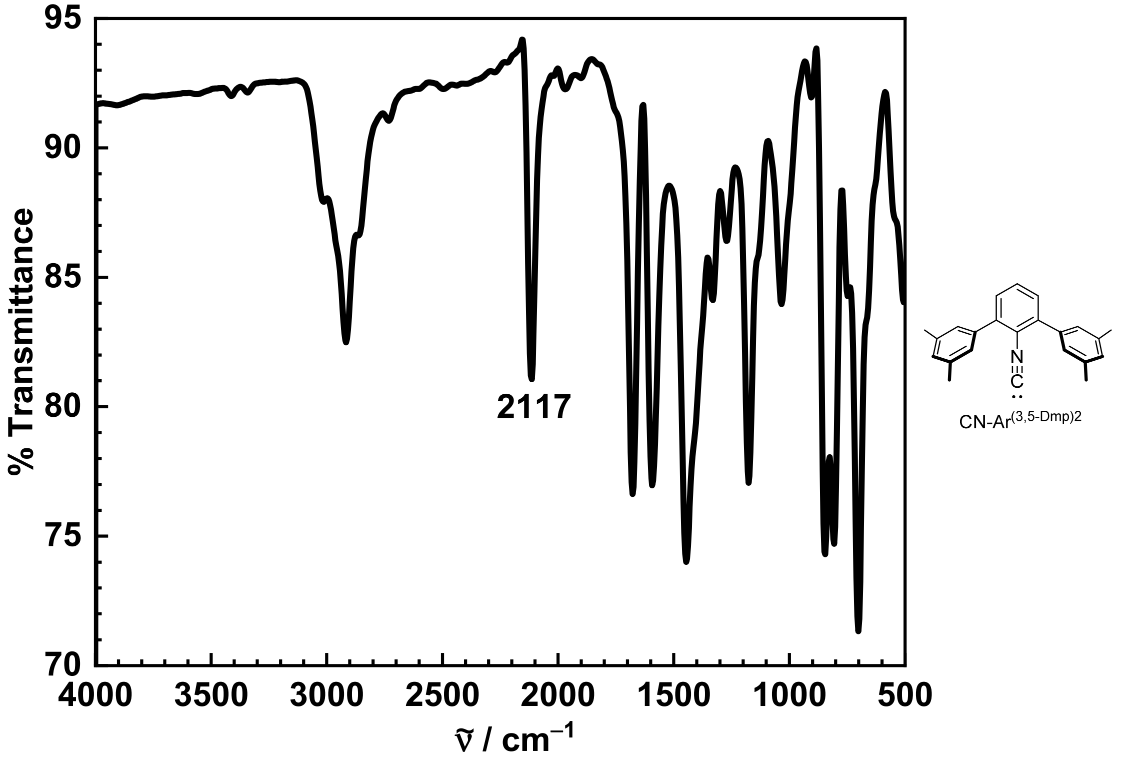

Fig. S22. FTIR spectrum of CN-Ar$^{(3,5\text{-Dmp})2}$, recorded on a neat sample. The C≡N stretching frequency ($\tilde{\nu}_{CN}$), in wavenumbers, is labeled.

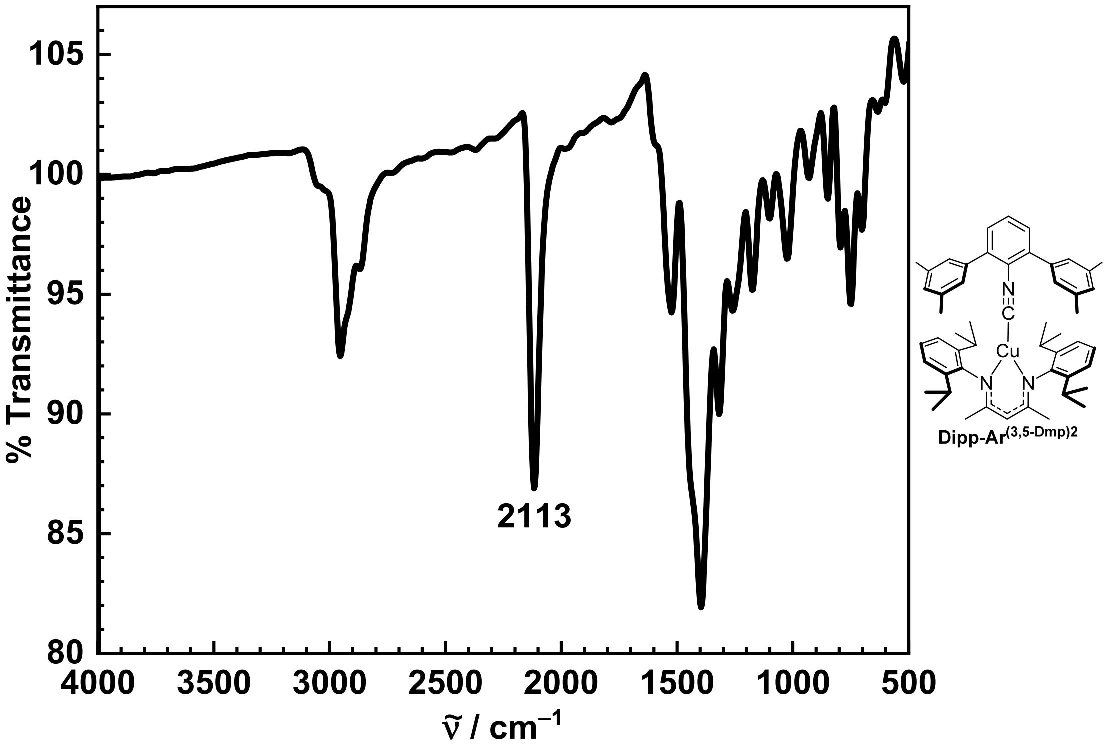

Fig. S23. FTIR spectrum of **Dipp-Ar$^{(3,5\text{-Dmp})2}$**, recorded on a neat sample. The C≡N stretching frequency ($\tilde{\nu}_{CN}$), in wavenumbers, is labeled.

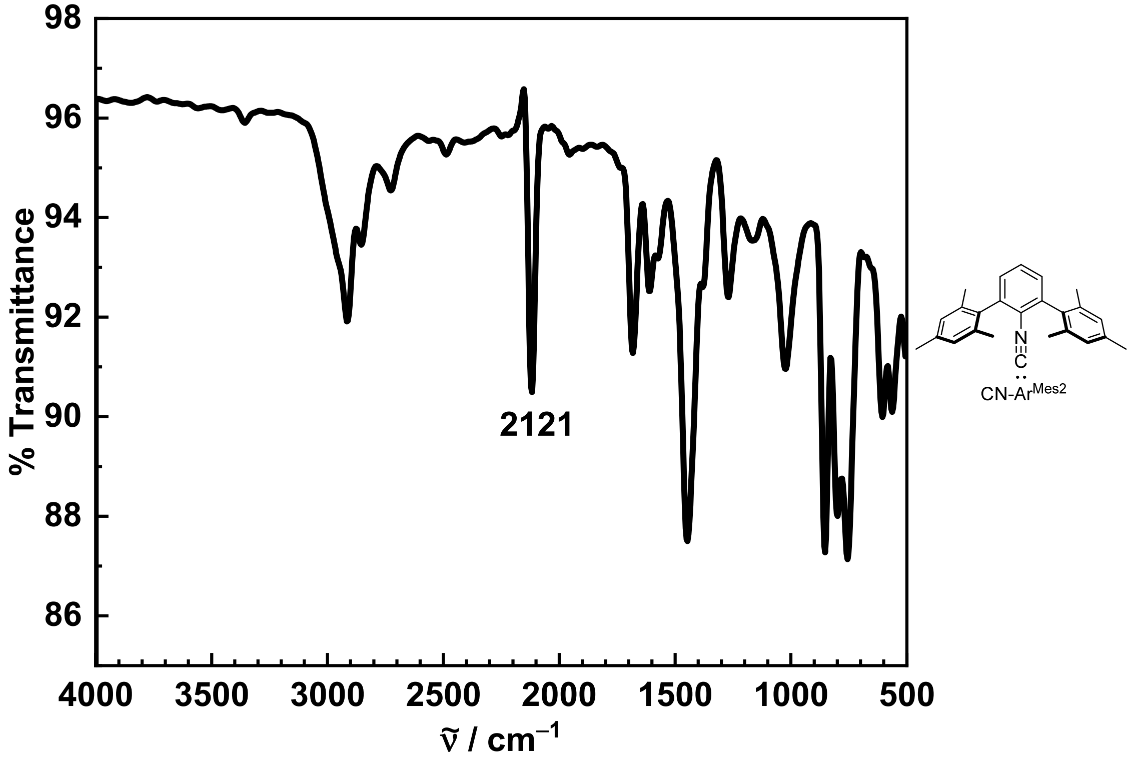

Fig. S24. FTIR spectrum of CN-Ar^{Mes2}, recorded on a neat sample. The C≡N stretching frequency ($\tilde{\nu}_{CN}$), in wavenumbers, is labeled.

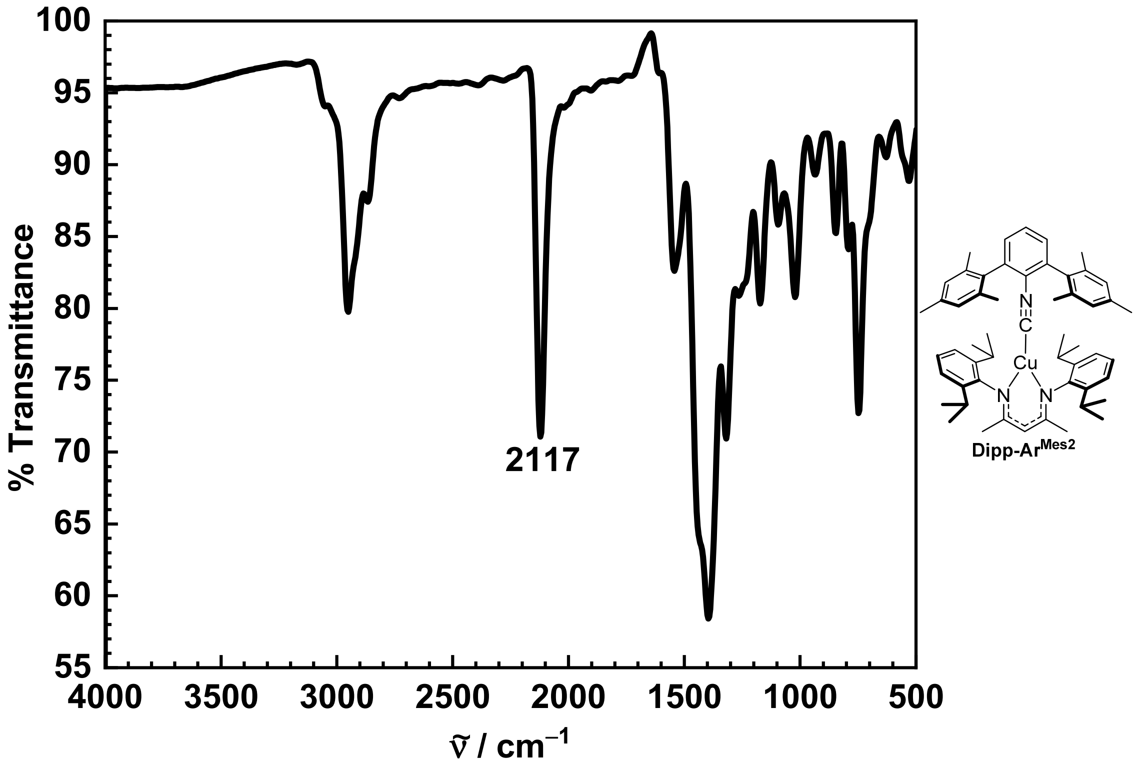

Fig. S25. FTIR spectrum of **Dipp-Ar^{Mes2}**, recorded on a neat sample. The C≡N stretching frequency ($\tilde{\nu}_{CN}$), in wavenumbers, is labeled.

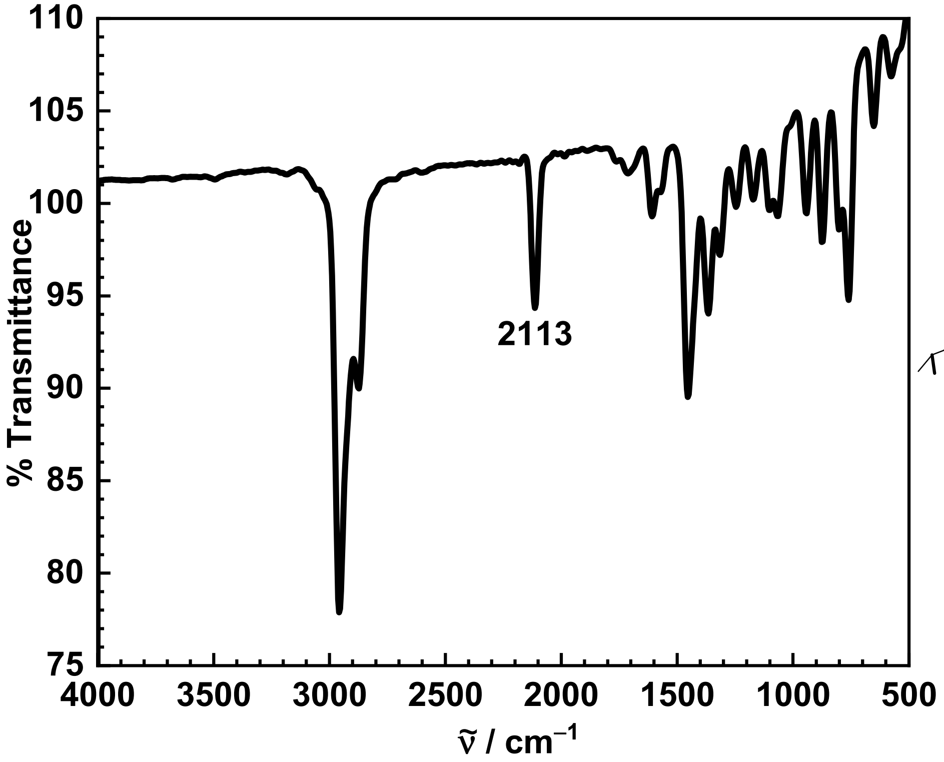

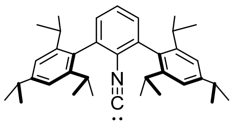

Fig. S26. FTIR spectrum of CN-Ar^{Tripp2}, recorded on a neat sample. The C≡N stretching frequency ($\tilde{\nu}_{CN}$), in wavenumbers, is labeled.

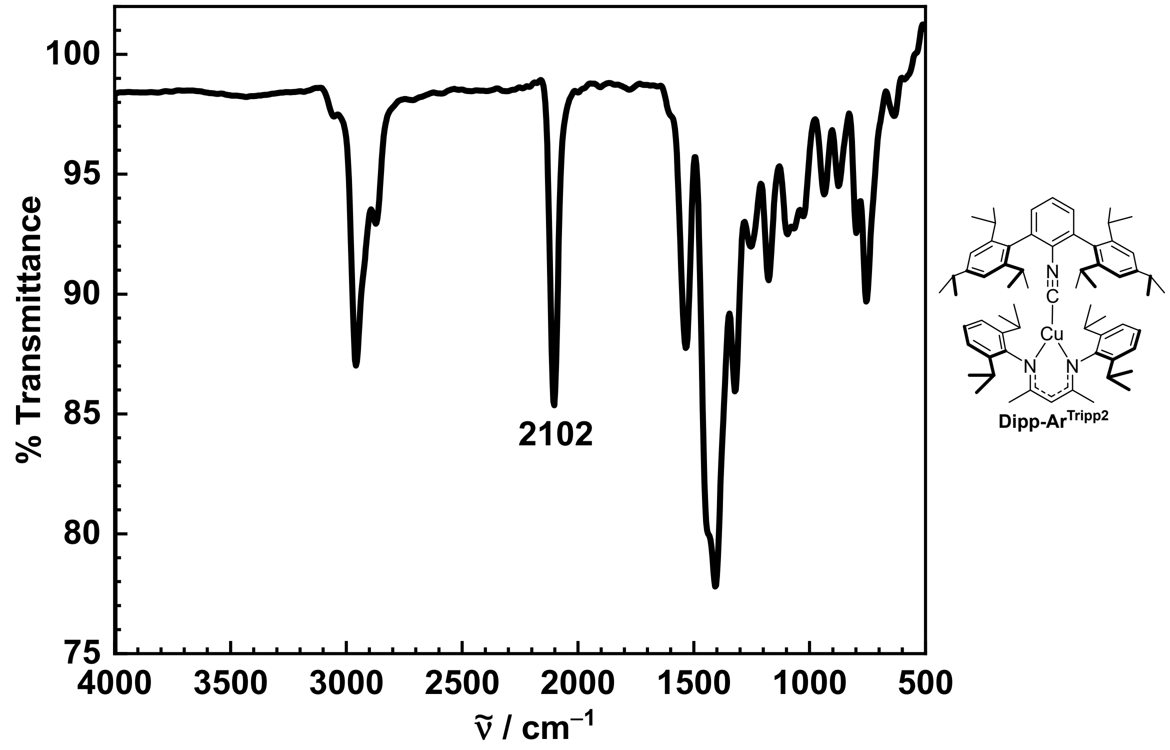

Fig. S27. FTIR spectrum of **Dipp-Ar^{Tripp2}**, recorded on a neat sample. The C≡N stretching frequency ($\tilde{\nu}_{CN}$), in wavenumbers, is labeled.

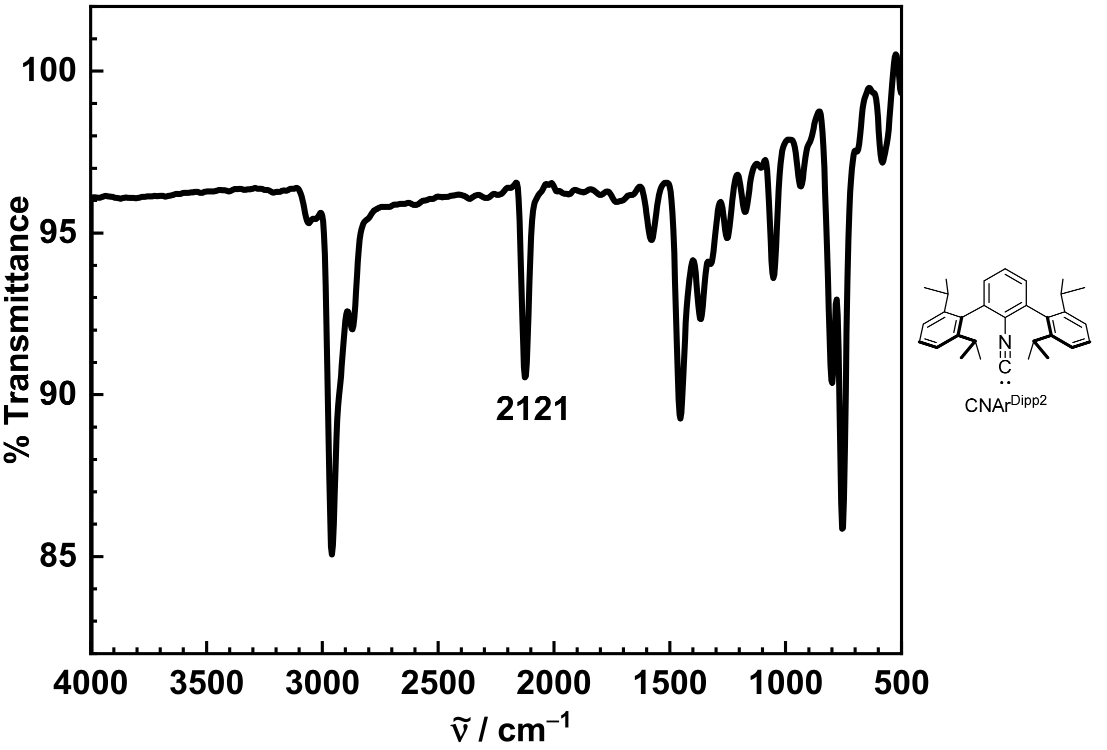

Fig. S28. FTIR spectrum of CN-Ar^{Dipp2}, recorded on a neat sample. The C≡N stretching frequency ($\tilde{\nu}_{CN}$), in wavenumbers, is labeled.

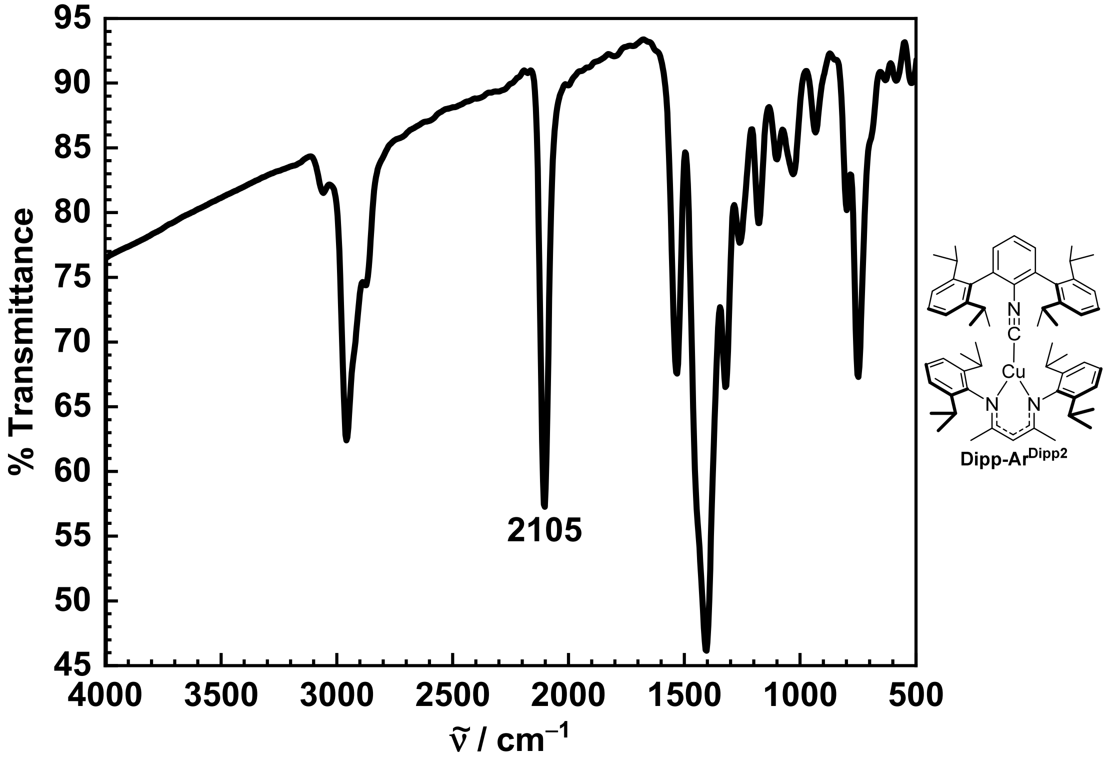

Fig. S29. FTIR spectrum of **Dipp-Ar^{Dipp2}**, recorded on a neat sample. The C≡N stretching frequency ($\tilde{\nu}_{CN}$), in wavenumbers, is labeled.

Table S1. Summary of C≡N stretching frequencies from FTIR spectra.

Compound	$\tilde{\nu}_{CN}$ / cm^{-1}
CN-Ar^{Ph2}	2113
**Ph-Ar^{Ph2}**	2117
**Cy-Ar^{Ph2}**	2102
**2,6-Dmp-Ar^{Ph2}**	2121
**Dipp-Ar^{Ph2}**	2113
CN-Ar$^{(3,5\text{-}Dmp)2}$	2117
**Dipp-Ar$^{(3,5\text{-}Dmp)2}$**	2113
CN-Ar^{Mes2}	2121
**Dipp-Ar^{Mes2}**	2117
CN-Ar^{Tripp2}	2113
**Dipp-Ar^{Tripp2}**	2102
CN-Ar^{Dipp}	2121
**Dipp-Ar^{Dipp2}**	2105

Table S2. Crystallographic summary for **Ph-Ar^{Ph2}**.

CCDC	2464277
**Crystal data**	
Chemical formula	$C_{36}H_{30}CuN_3$
M_r	568.17
Crystal system, space group	Monoclinic, $P2_1/c$
Temperature (K)	100
α, β, γ (Å)	21.7604(4), 11.5604(2), 11.3630(2)
β (°)	102.324(1)
V (Å^3)	2792.59(9)
Z	4
Radiation type	Cu Kα
μ (mm^{-1})	1.32
Crystal size (mm)	0.15 × 0.03 × 0.01
**Data collection**	
T_{min}, T_{max}	0.665, 0.753
No. of measured, independent and observed [$I > 2\sigma(I)$] reflections	53189, 5110, 4565
R_{int}	0.041
$(\sin \theta/\lambda)_{max}$ (Å^{-1})	0.603
Refinement	
$R[F^2 > 2\sigma(F^2)]$, $wR(F^2)$, S	0.032, 0.085, 1.06
No. of reflections	5110
No. of parameters	363
$\Delta\rho_{max}$, $\Delta\rho_{min}$ (e Å^{-3})	0.32, −0.59

Table S3. Crystallographic summary for **Cy-Ar^{Ph2}**.

**CCDC**	2464278
Crystal data	
Chemical formula	$C_{36}H_{42}CuN_3$
M_r	580.26
Crystal system, space group	Monoclinic, $P2_1/c$
Temperature (K)	150
a, *b*, *c* (Å)	10.745(10), 23.32(2), 13.062(12)
β (°)	105.103(10)
V (Å^3)	3160(5)
Z	4
Radiation type	Mo Kα
m (mm^{-1})	0.72
Crystal size (mm)	0.36 × 0.35 × 0.04
**Data collection**	
T_{min}, T_{max}	0.571, 0.746
No. of measured, independent and observed [$I > 2\sigma(I)$] reflections	21271, 6957, 3480
R_{int}	0.118
$(\sin \theta/\lambda)_{max}$ (Å^{-1})	0.641
**Refinement**	
$R[F^2 > 2\sigma(F^2)]$, $wR(F^2)$, S	0.064, 0.127, 0.99
No. of reflections	6957
No. of parameters	363
$\Delta\rho_{max}$, $\Delta\rho_{min}$ (e Å^{-3})	0.40, −0.70

Table S4. Crystallographic summary for **2,6-Dmp-Ar^{Ph2}**.

CCDC	2464279
**Crystal data**	
Chemical formula	$C_{40}H_{38}CuN_3$
M_r	624.027
Crystal system, space group	Monoclinic, $P2_1$
Temperature (K)	150
a, *b*, *c* (Å)	20.8298(8), 11.4789(4), 29.6726(14)
β (°)	110.488(1)
V (Å^3)	6646.0(5)
Z	8
Radiation type	Mo *K*α
m (mm^{-1})	0.69
Crystal size (mm)	0.29 × 0.16 × 0.11
**Data collection**	
T_{min}, T_{max}	
No. of measured, independent and observed [$I > 2\sigma(I)$] reflections	104701, 29188, 23344
R_{int}	0.037
$(\sin \theta/\lambda)_{max}$ (Å^{-1})	0.641
Refinement	
$R[F^2 > 2\sigma(F^2)]$, $wR(F^2)$, S	0.037, 0.092, 1.02
No. of reflections	29188
No. of parameters	1656
No. of restraints	313
$\Delta\rho_{max}$, $\Delta\rho_{min}$ (e Å^{-3})	0.46, −0.25
Absolute structure	Refined as an inversion twin.
Absolute structure parameter	0.069(9)

Table S5. Crystallographic summary for **Dipp-Ar^{Ph2}**.

CCDC	2464280
**Crystal data**	
Chemical formula	$C_{48}H_{54}CuN_3$
M_r	736.48
Crystal system, space group	Triclinic, $P\bar{1}$
Temperature (K)	150
a, *b*, *c* (Å)	12.0288(9), 12.5853(9), 16.2705(12)
α, β, γ (°)	74.800(3), 72.478(3), 63.532(3)
V (Å^3)	2078.9(3)
Z	2
Radiation type	Cu *K*α
m (mm^{-1})	1.00
Crystal size (mm)	0.34 × 0.17 × 0.09
**Data collection**	
T_{min}, T_{max}	0.672, 0.753
No. of measured, independent and observed [$I > 2\sigma(I)$] reflections	22181, 7114, 6857
R_{int}	0.027
(sin θ/λ)$_{max}$ (Å^{-1})	0.595
**Refinement**	
$R[F^2 > 2\sigma(F^2)]$, $wR(F^2)$, S	0.032, 0.085, 1.06
No. of reflections	7114
No. of parameters	571
No. of restraints	591
$\Delta\rho_{max}$, $\Delta\rho_{min}$ (e Å^{-3})	0.25, −0.33

Table S6. Crystallographic summary for **Dipp-Ar$^{(3,5\text{-}Dmp)2}$**.

CCDC	2464281
**Crystal data**	
Chemical formula	$C_{52}H_{62}CuN_3$
M_r	792.58
Crystal system, space group	Monoclinic, $P2_1$
Temperature (K)	150
a, *b*, *c* (Å)	11.9121(7), 23.6724(13), 16.9003(9)
β (°)	105.257(2)
V (Å^3)	4597.7(4)
Z	4
Radiation type	Cu *K*α
m (mm^{-1})	0.93
Crystal size (mm)	0.39 × 0.34 × 0.21
**Data collection**	
T_{min}, T_{max}	0.656, 0.753
No. of measured, independent and observed [$I > 2\sigma(I)$] reflections	21983, 13158, 13007
R_{int}	0.019
$(\sin \theta/\lambda)_{max}$ (Å^{-1})	0.595
**Refinement**	
$R[F^2 > 2\sigma(F^2)]$, $wR(F^2)$, S	0.027, 0.067, 1.07
No. of reflections	13158
No. of parameters	1021
No. of restraints	1
$\Delta\rho_{max}$, $\Delta\rho_{min}$ (e Å^{-3})	0.20, −0.32
Absolute structure	Flack x determined using 4866 quotients [(I+)-(I-)]/[(I+)+(I-)] (Parsons, Flack and Wagner, Acta Cryst. B69 (2013) 249-259).
Absolute structure parameter	0.014 (6)

Table S7. Crystallographic summary for **Dipp-Ar^{Mes2}**.

CCDC	2464282
**Crystal data**	
Chemical formula	$C_{54}H_{66}CuN_3$
M_r	820.63
Crystal system, space group	Triclinic, $P\bar{1}$
Temperature (K)	123
a, *b*, *c* (Å)	11.455(2), 11.955(2), 21.253(4)
α, β, γ (°)	79.082(2), 89.214(2), 67.827(2)
V (Å^3)	2641.2(8)
Z	2
Radiation type	Mo *K*α
m (mm^{-1})	0.45
Crystal size (mm)	0.36 × 0.25 × 0.06
**Data collection**	
T_{min}, T_{max}	0.696, 0.746
No. of measured, independent and observed [$I > 2\sigma(I)$] reflections	36365, 11646, 9569
R_{int}	0.036
$(\sin \theta/\lambda)_{max}$ (Å^{-1})	0.641
**Refinement**	
$R[F^2 > 2\sigma(F^2)]$, $wR(F^2)$, S	0.040, 0.116, 1.06
No. of reflections	11646
No. of parameters	559
No. of restraints	182
$\Delta\rho_{max}$, $\Delta\rho_{min}$ (e Å^{-3})	0.33, −0.34

Table S8. Crystallographic summary for **Dipp-Ar^{Tripp2}**.

CCDC	2464283
**Crystal data**	
Chemical formula	$C_{66}H_{90}CuN_3$
M_r	988.94
Crystal system, space group	Monoclinic, $P2_1/n$
Temperature (K)	123
a, *b*, *c* (Å)	17.3057(15), 18.6955(16), 18.0781(15)
β (°)	91.517(1)
V (Å^3)	5846.9(9)
Z	4
Radiation type	Mo *K*α
m (mm^{-1})	0.41
Crystal size (mm)	0.35 × 0.28 × 0.15
**Data collection**	
T_{min}, T_{max}	0.675, 0.746
No. of measured, independent and observed [$I \geq 2\sigma(I)$] reflections	33299, 11905, 9887
R_{int}	0.025
$(\sin \theta/\lambda)_{max}$ (Å^{-1})	0.625
**Refinement**	
$R[F^2 > 2\sigma(F^2)]$, $wR(F^2)$, S	0.052, 0.154, 1.03
No. of reflections	11905
No. of parameters	671
No. of restraints	162
$\Delta\rho_{max}$, $\Delta\rho_{min}$ (e Å^{-3})	1.16, −0.50

Table S9. Crystallographic summary for **Dipp-Ar$^{\mathbf{Dipp2}}$**.

CCDC	2464284
**Crystal data**	
Chemical formula	$C_{67}H_{86}CuN_3$
M_r	996.92
Crystal system, space group	Orthorhombic, $P2_12_12_1$
Temperature (K)	150
a, *b*, *c* (Å)	14.263(3), 18.014(4), 22.688(4)
V (Å^3)	5829(2)
Z	4
Radiation type	Mo $K\alpha$
m (mm^{-1})	0.42
Crystal size (mm)	0.33 × 0.28 × 0.20
**Data collection**	
T_{min}, T_{max}	0.718, 0.746
No. of measured, independent and observed [$I > 2\sigma(I)$] reflections	35935, 13228, 11845
R_{int}	0.024
$(\sin \theta/\lambda)_{max}$ (Å^{-1})	0.649
**Refinement**	
$R[F^2 > 2\sigma(F^2)]$, $wR(F^2)$, S	0.034, 0.089, 1.03
No. of reflections	13228
No. of parameters	681
No. of restraints	165
$\Delta\rho_{max}$, $\Delta\rho_{min}$ (e Å^{-3})	0.31, −0.21
Absolute structure	Flack x determined using 4809 quotients [(I+)-(I-)]/[(I+)+(I-)] (Parsons, Flack and Wagner, Acta Cryst. B69 (2013) 249-259).
Absolute structure parameter	0.006 (3)

Table S10. Selected structural metrics of Cu(I) complexes determined by single-crystal X-ray diffraction. C1 and N3 are the carbon and nitrogen atoms of the isocyanide ligand and N1 and N2 are the nitrogen atoms of the RNacNac ligand. The structure of **2,6-Dmp-Ar^{Ph2}** has four crystallographically independent molecules and the structure of **Dipp-Ar$^{(3,5\text{-}Dmp)2}$** has two; all independent sets of metrics are provided.

	C1–N3 / Å	Cu–C1 / Å	Cu–N1 / Å	Cu–N2 / Å	N1–Cu–N2 / °	Cu–C1–N1 / °
**Ph-Ar^{Ph2}**	1.162(3)	1.8210(19)	1.9500(16)	1.9491(15)	98.54(6)	178.10(17)
**Cy-Ar^{Ph2}**	1.165(5)	1.819(5)	1.943(3)	1.948(3)	100.19(14)	174.0(4)
**2,6-Dmp-Ar^{Ph2}**	1.165(2)	1.814(4)	1.943(4)	1.925(3)	96.85(15)	172.7(4)
	1.158(5)	1.815(4)	1.959(3)	1.923(3)	97.76(14)	171.2(4)
	1.156(5)	1.825(4)	1.954(3)	1.926(3)	97.79(14)	171.9(4)
	1.161(5)	1.818(4)	1.962(3)	1.934(3)	96.89(14)	174.2(4)
**Dipp-Ar^{Ph2}**	1.162(2)	1.8116(15)	1.9367(12)	1.9414(12)	97.43(5)	176.81(12)
**Dipp-Ar$^{(3,5\text{-}Dmp)2}$**	1.154(3)	1.820(2)	1.9533(17)	1.9308(17)	98.19(7)	170.41(19)
	1.160(3)	1.826(2)	1.9510(17)	1.9589(17)	97.34(7)	177.25(18)
**Dipp-Ar^{Mes2}**	1.162(2)	1.8160(18)	1.9408(14)	1.9340(15)	96.77(6)	178.25 (16)
**Dipp-Ar^{Tripp2}**	1.164(3)	1.845(2)	1.9585(18)	1.9657(18)	94.65(8)	178.94(18)
**Dipp-Ar^{Dipp2}**	1.186(3)	1.841(2)	1.970(2)	1.970(2)	96.38(9)	175.9(2)

Table S11. Dihedral angles between the Cu-RNacNac mean plane and the central aryl ring of the substituted *m*-terphenyl isocyanide ligand, determined from single-crystal X-ray structures. The structure of **2,6-Dmp-Ar^{Ph2}** has four crystallographically independent molecules and the structure of **Dipp-Ar$^{(3,5\text{-}Dmp)2}$** has two; all independent sets of metrics are provided. The software Mercury 2020.1 was used to calculate the mean planes and the angles between them.

Complex	Dihedral Angle / °
**Ph-Ar^{Ph2}**	52.68
**Cy-Ar^{Ph2}**	56.25
**2,6-Dmp-Ar^{Ph2}**	88.12
	89.45
	87.72
	87.53
**Dipp-Ar^{Ph2}**	77.20
**Dipp-Ar$^{(3,5\text{-}Dmp)2}$**	70.94
	75.11
**Dipp-Ar^{Mes2}**	88.06
**Dipp-Ar^{Tripp2}**	87.29
**Dipp-Ar^{Dipp2}**	35.64

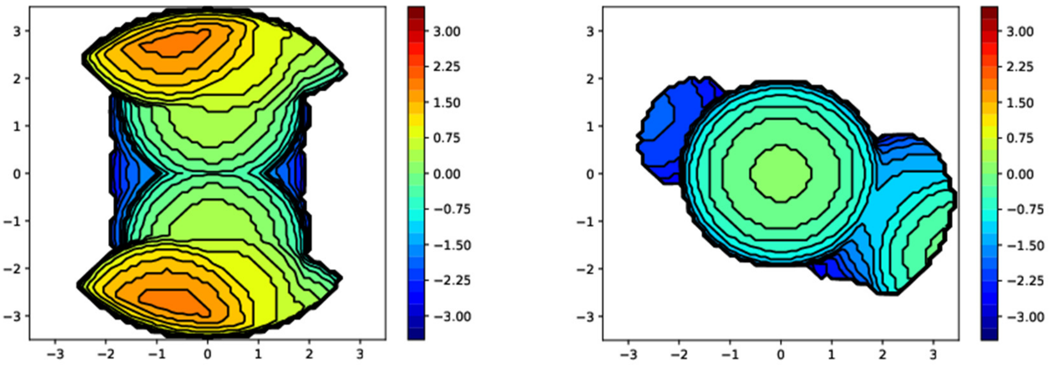

Ph-Ar^{Ph2}

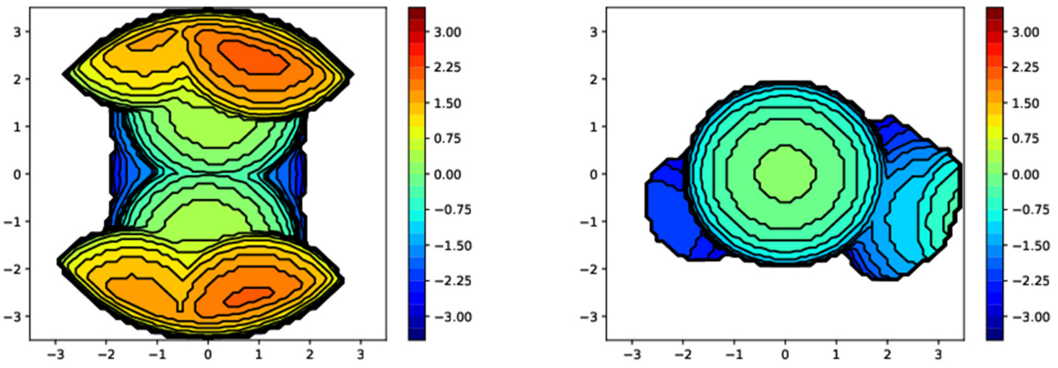

Cy-Ar^{Ph2}

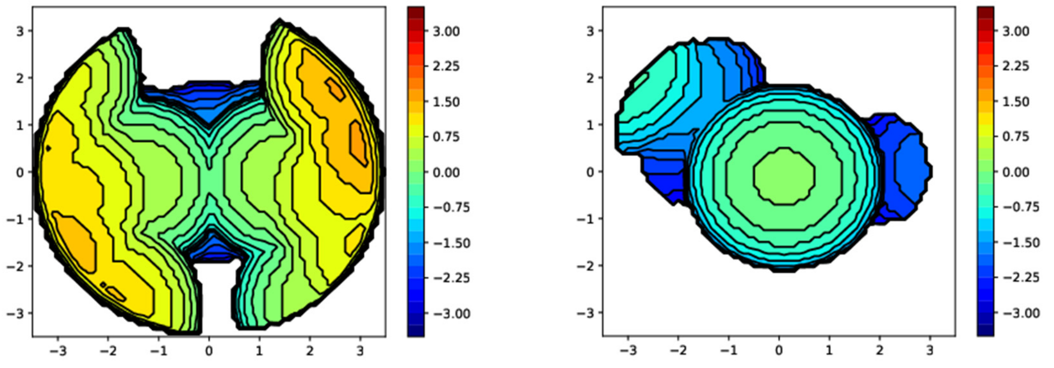

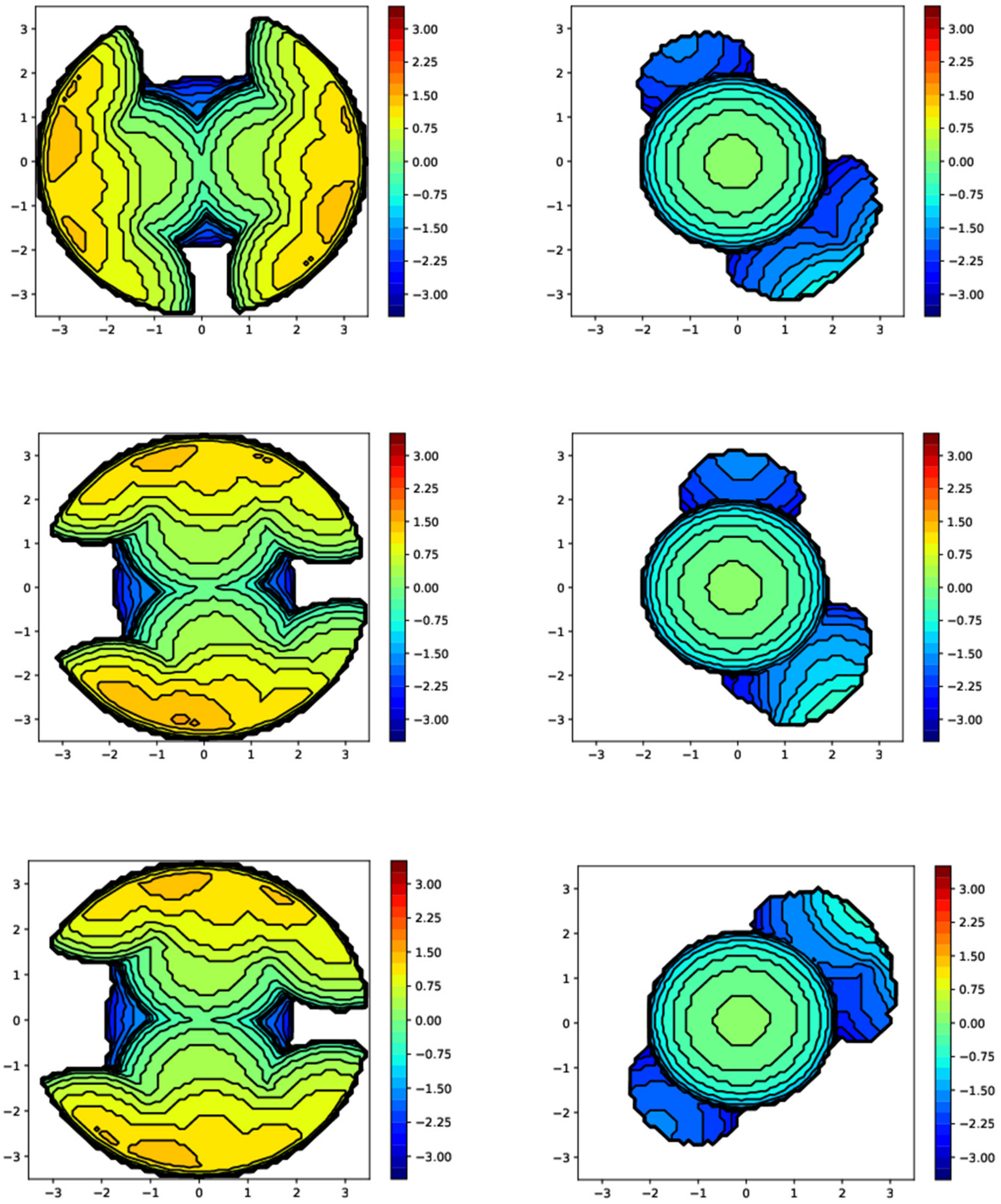

2,6-Dmp-Ar^{Ph2}

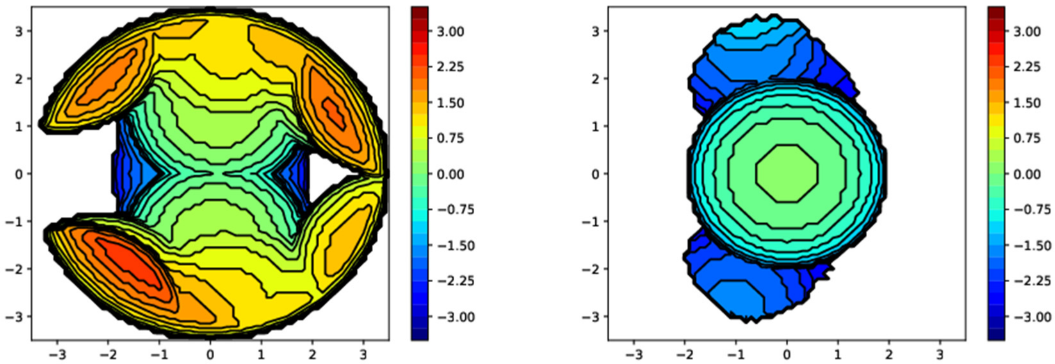

Dipp-Ar^{Ph2}

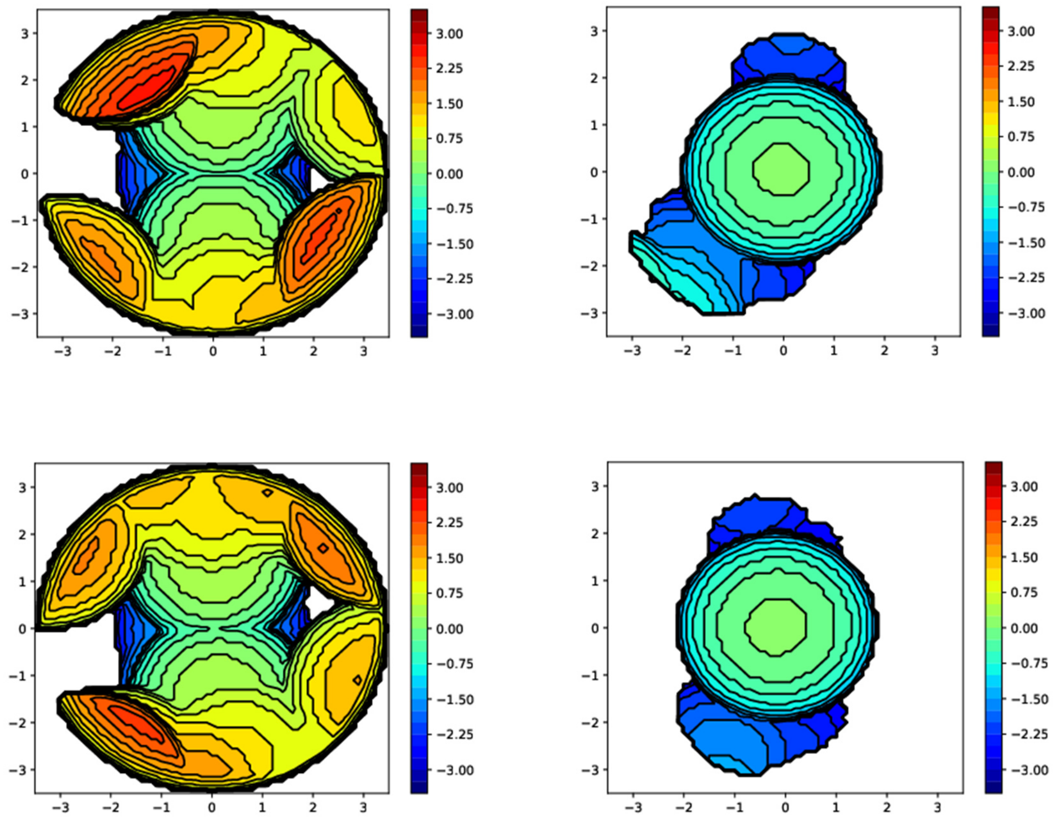

Dipp-Ar$^{(3,5\text{-}Dmp)2}$

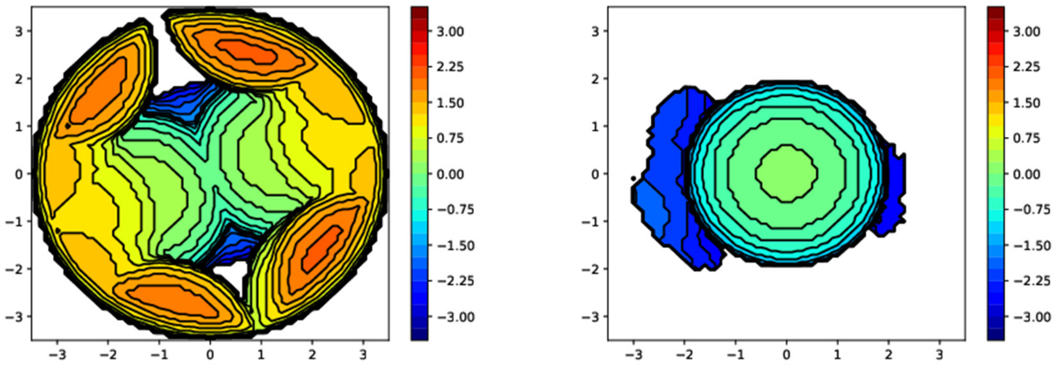

Dipp-Ar^{Mes2}

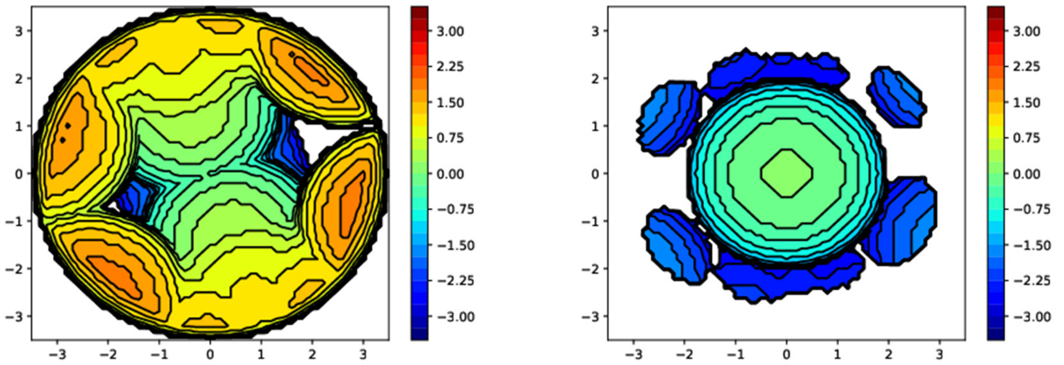

Dipp-Ar^{Tripp2}

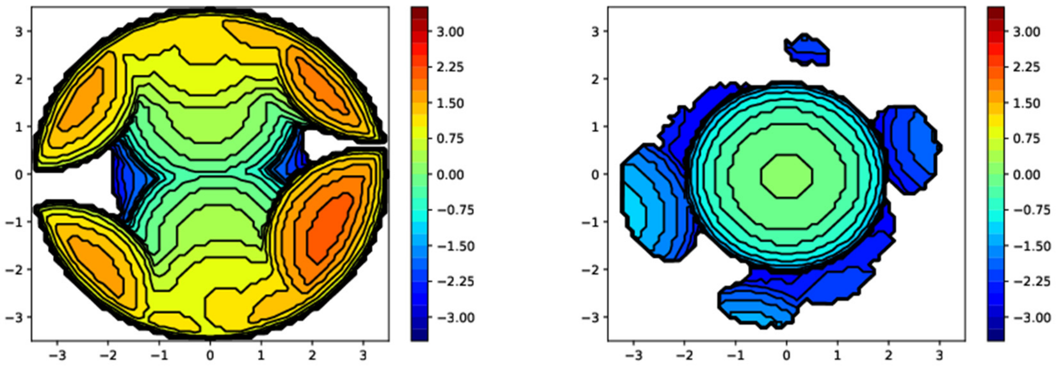

Dipp-Ar^{Dipp2}

Fig. S30. Topographical steric maps of the Cu(RNacNac)(CN-Ar^{X2}) complexes. For the complexes with more than one crystallographically independent molecule, individual plots for each molecule are shown. (Left: RNacNac, Right: isocyanide ligand)

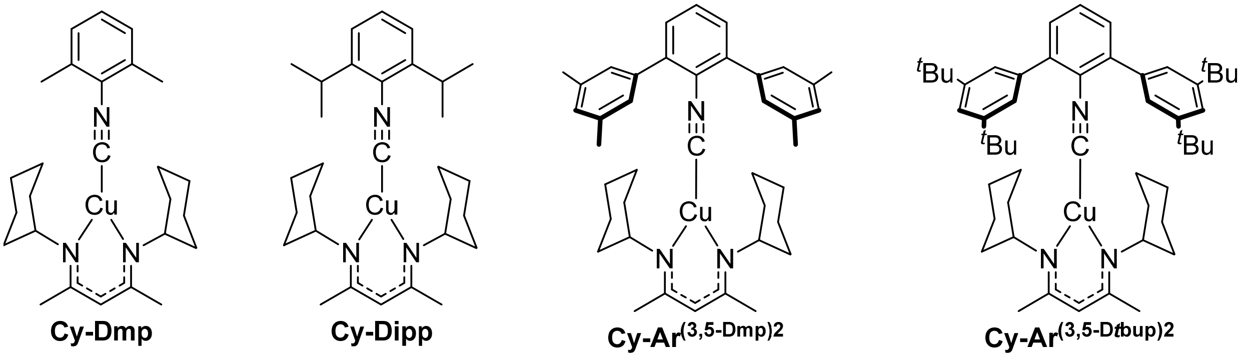

Fig. S31. Chemical structures of previously reported complexes[7] summarized in Table 3 and described in the main text.

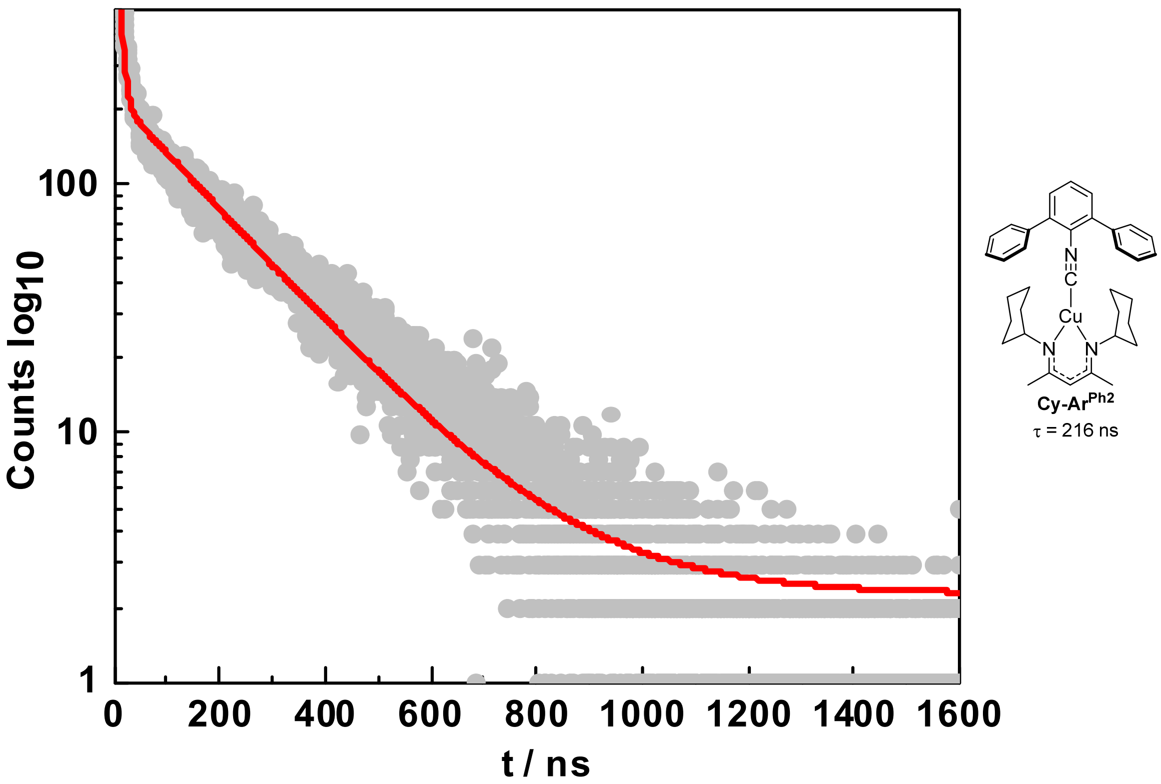

Fig. S32. Time-resolved photoluminescence decay of **Cy-Ar^{Ph2}** obtained by time-correlated single photon counting (TCSPC). The decay trace was recorded in toluene with 330 nm excitation wavelength and was best fit by a biexponential equation.

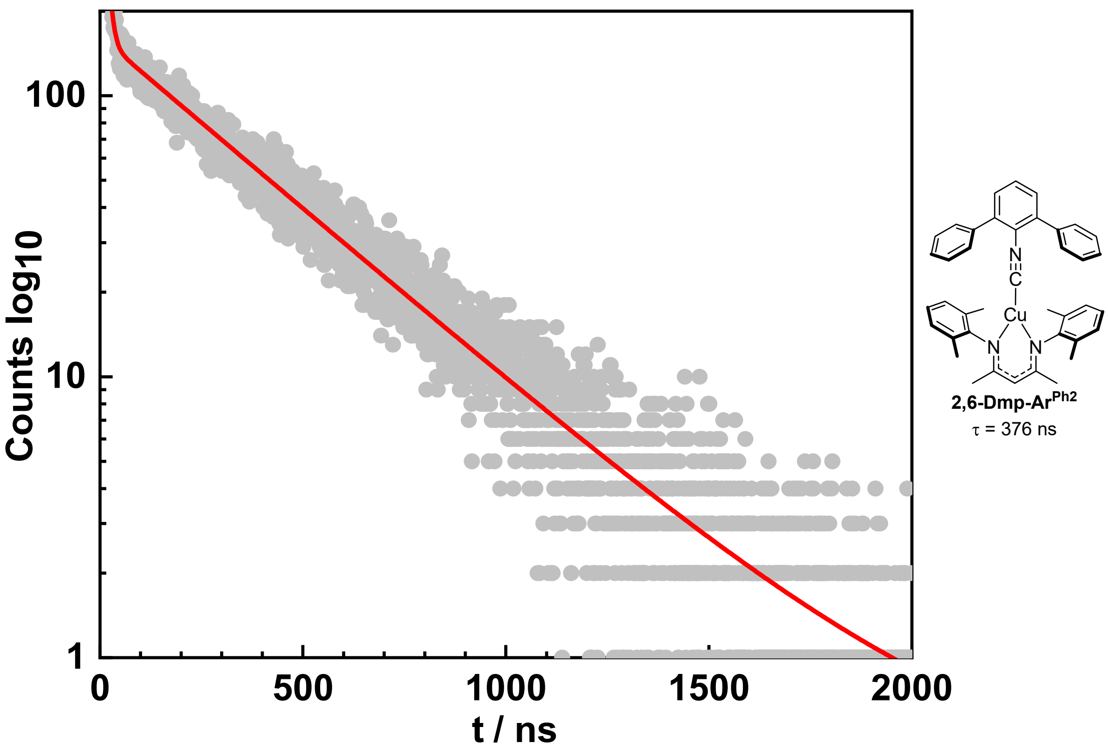

Fig. S33. Time-resolved photoluminescence decay of **2,6-Dmp-Ar^{Ph2}** obtained by time-correlated single photon counting (TCSPC). The decay trace was recorded in toluene with 330 nm excitation wavelength and was best fit by a biexponential equation.

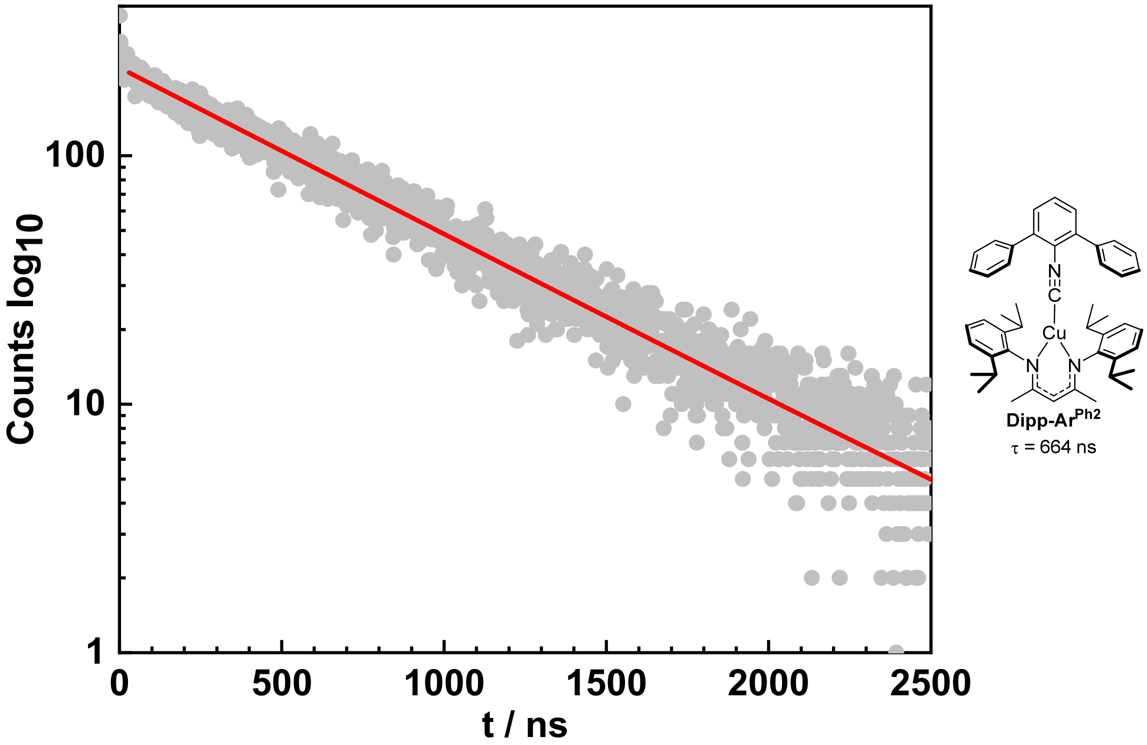

Fig. S34. Time-resolved photoluminescence decay of **Dipp-Ar^{Ph2}** obtained by time-correlated single photon counting (TCSPC). The decay trace was recorded in toluene with 330 nm excitation wavelength and was best fit by a monoexponential equation.

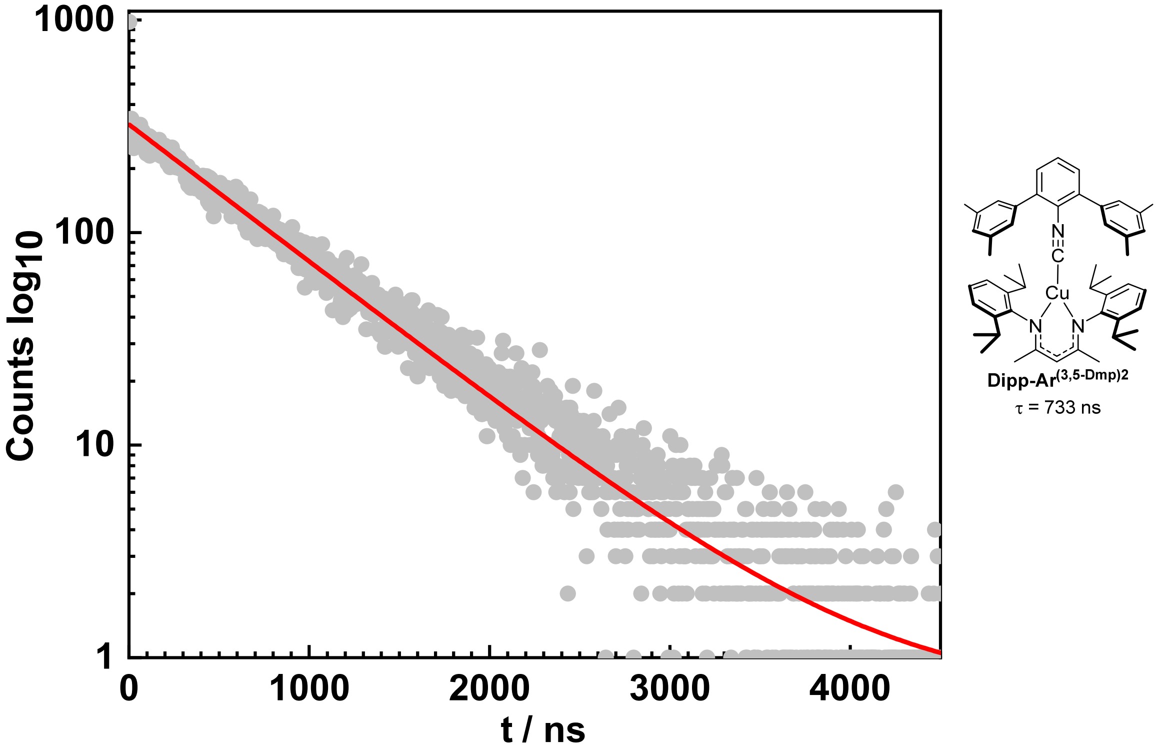

Fig. S35. Time-resolved photoluminescence decay of **Dipp-Ar(3,5-Dmp)2** obtained by time-correlated single photon counting (TCSPC). The decay trace was recorded in toluene with 359 nm excitation wavelength and was best fit by a monoexponential equation.

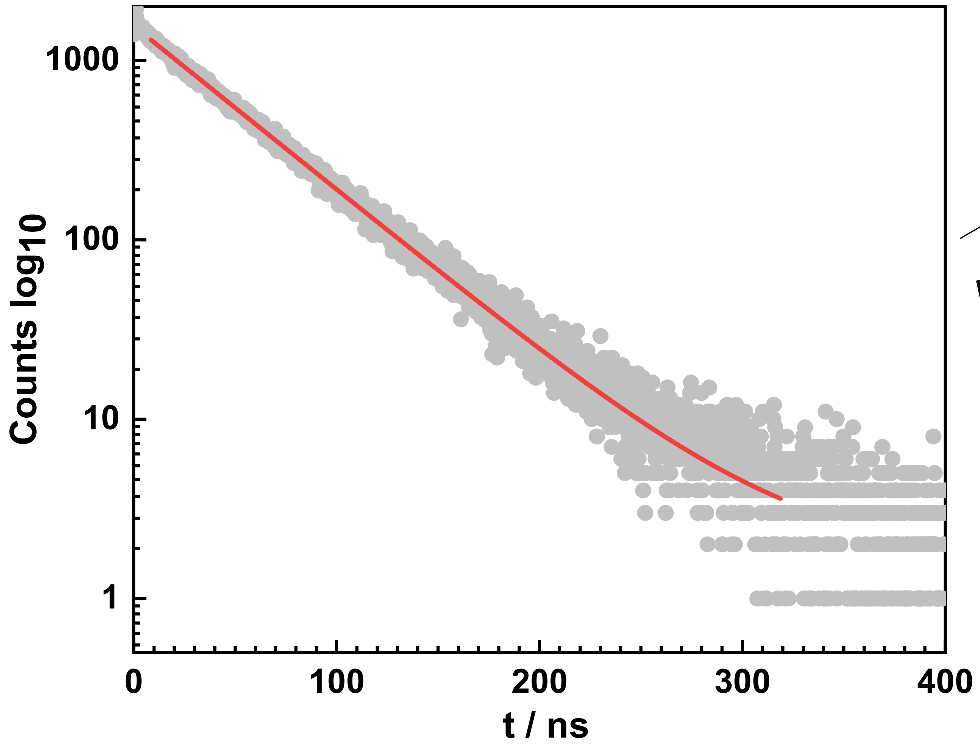

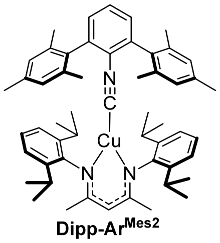

Fig. S36. Time-resolved photoluminescence decay of **Dipp-Ar^{Mes2}** obtained by time-correlated single photon counting (TCSPC). The decay trace was recorded in toluene with 330 nm excitation wavelength and was best fit by a biexponential equation.

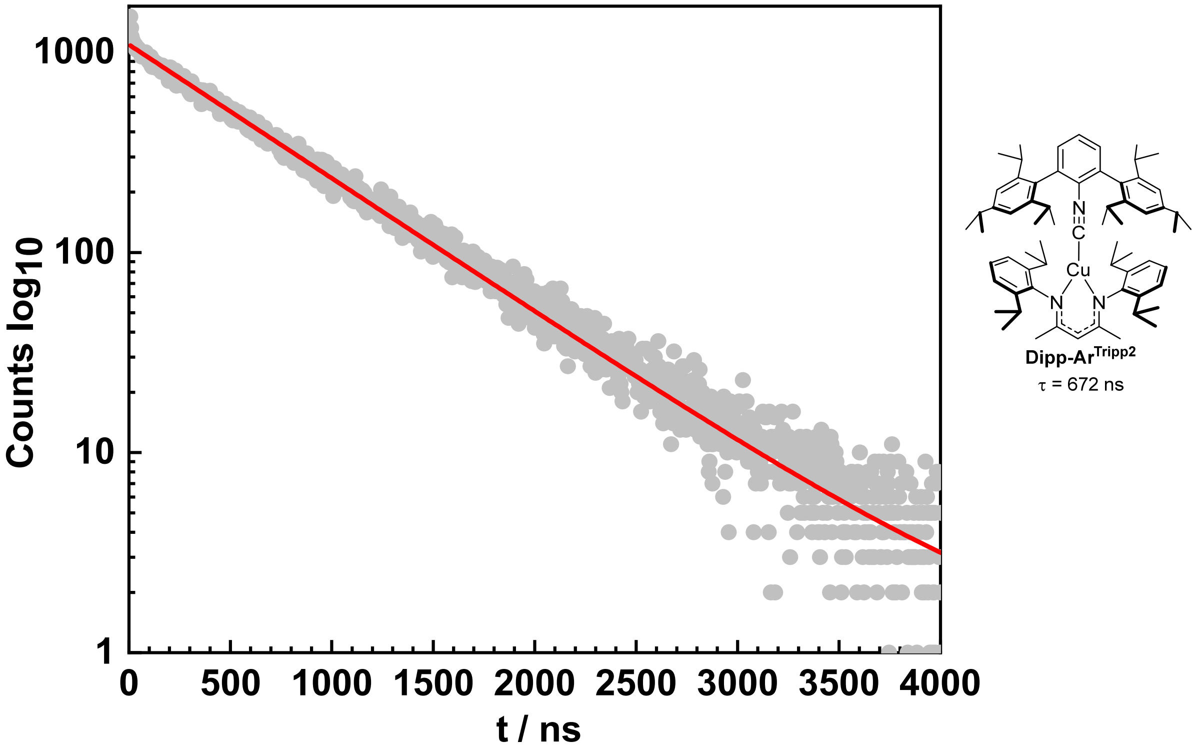

Fig. S37. Time-resolved photoluminescence decay of **Dipp-Ar^{Tripp2}** obtained by time-correlated single photon counting (TCSPC). The decay trace was recorded in toluene with 330 nm excitation wavelength and was best fit by a monoexponential equation.

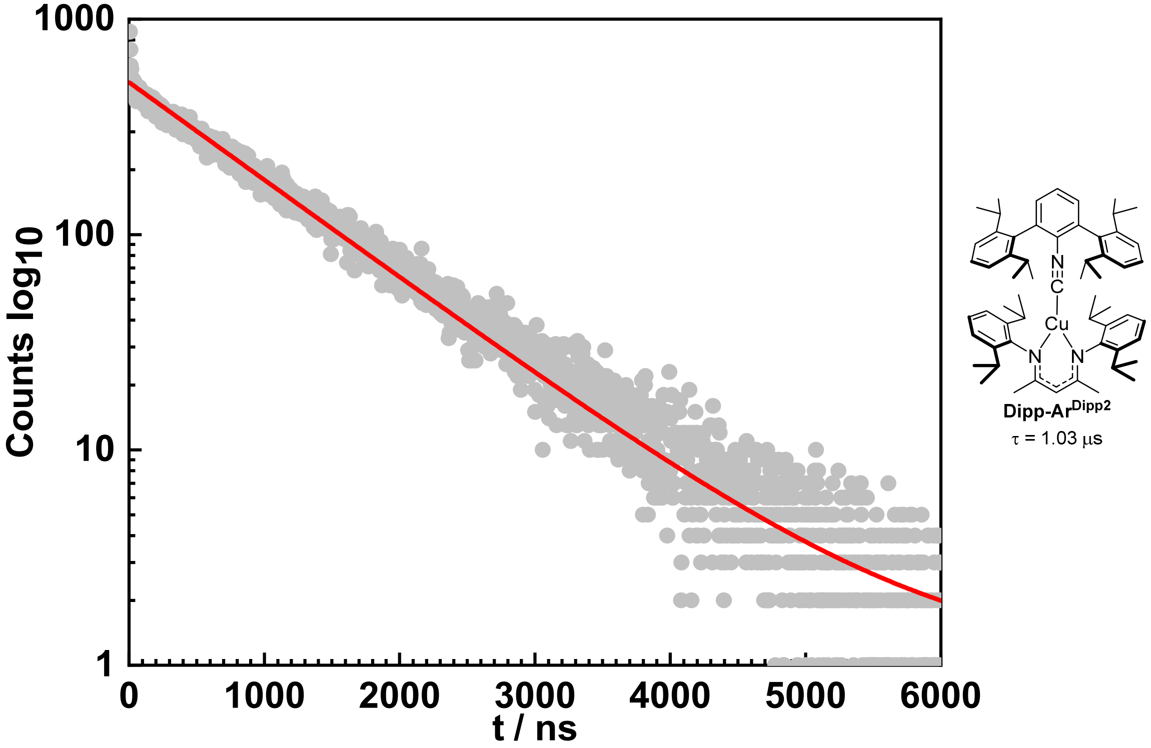

Fig. S38. Time-resolved photoluminescence decay of **Dipp-Ar^{Dipp2}** obtained by time-correlated single photon counting (TCSPC). The decay trace was recorded in toluene with 330 nm excitation wavelength and was best fit by a monoexponential equation.

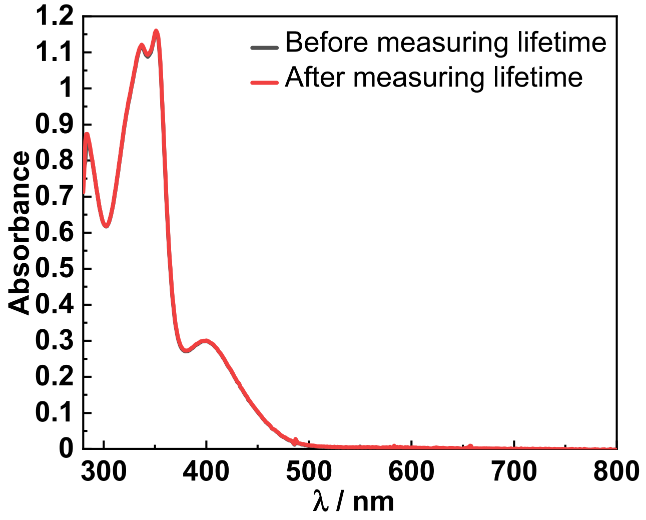

Fig. S39. UV-vis spectra before (black solid line) and after (red solid line) measuring the lifetime of **Cy-Ar^{Ph2}** using TCSPC.

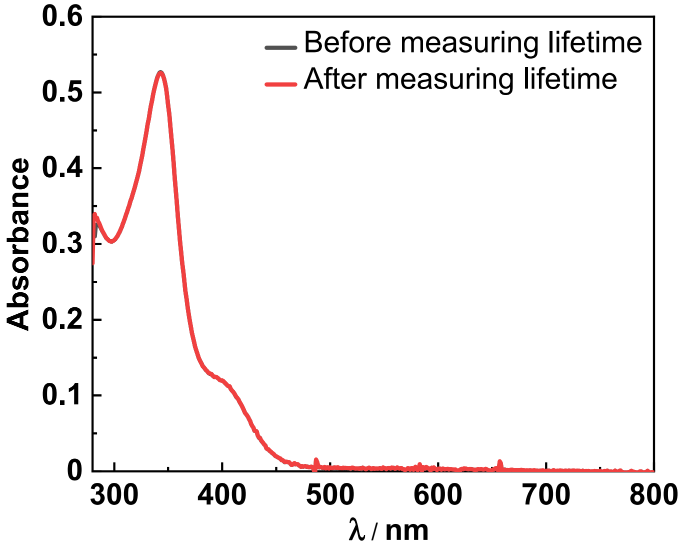

Fig. S40. UV-vis spectra before (black solid line) and after (red solid line) measuring the lifetime of **2,6-Dmp-Ar^{Ph2}**using TCSPC.

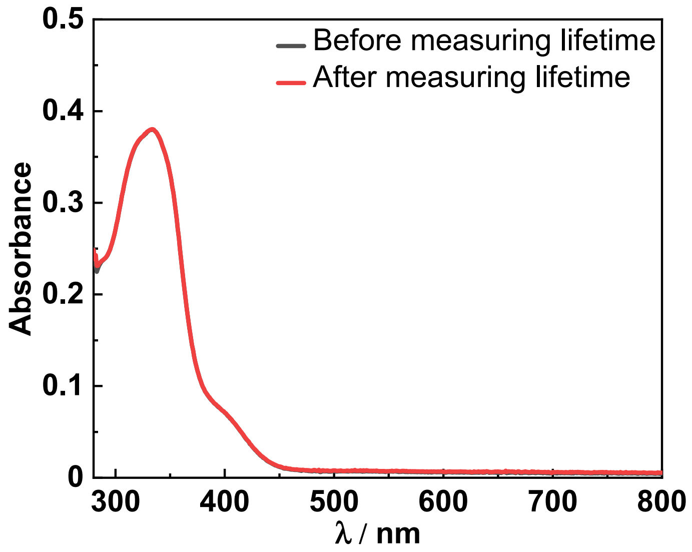

Fig. S41. UV-vis spectra before (black solid line) and after (red solid line) measuring the lifetime of **Dipp-Ar^{Ph2}** using TCSPC .

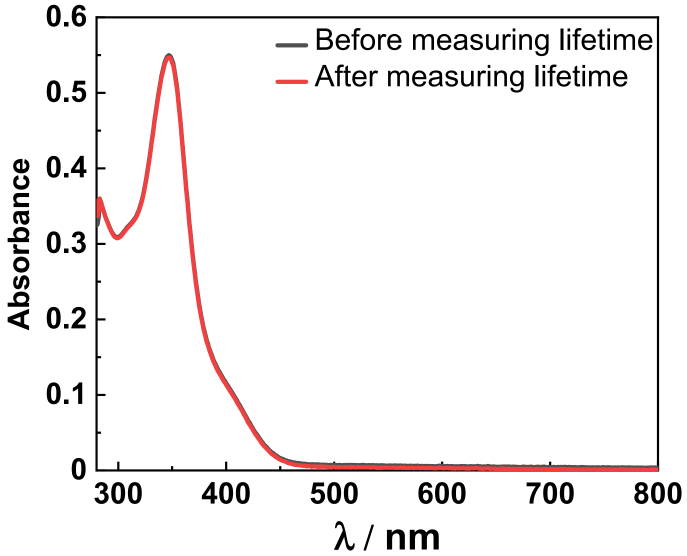

Fig. S42. UV-vis spectra before (black solid line) and after (red solid line) measuring the lifetime of **Dipp-Ar$^{(3,5\text{-}Dmp)2}$** using TCSPC.

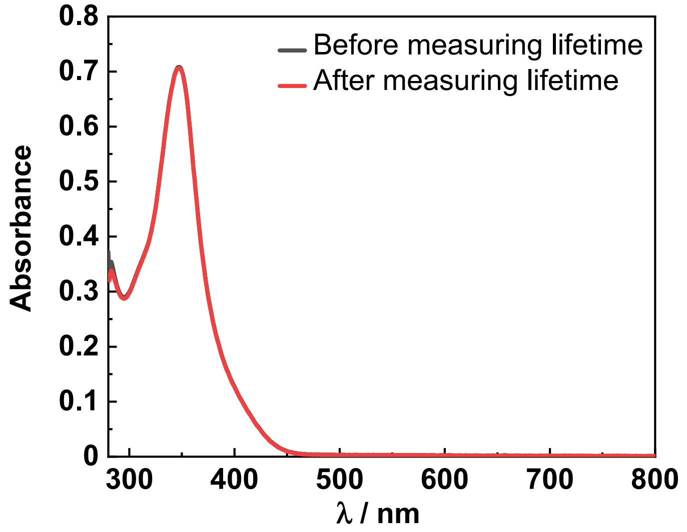

Fig. S43. UV-vis spectra before (black solid line) and after (red solid line) measuring the lifetime of **Dipp**-Ar^{Mes2} using TCSPC.

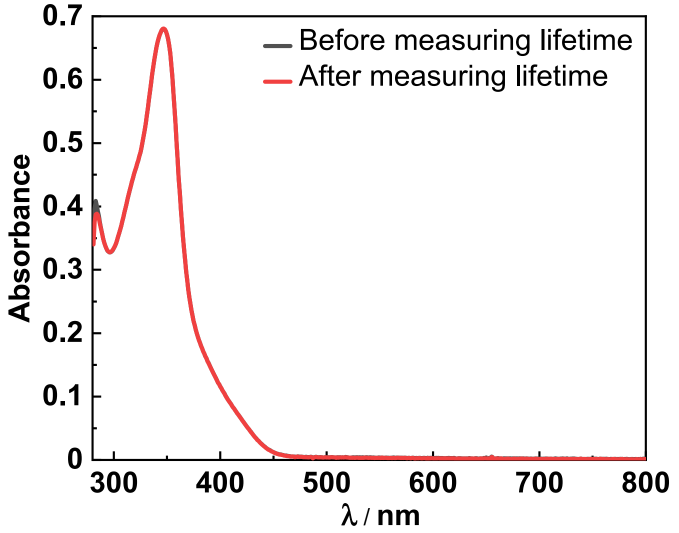

Fig. S44. UV-vis spectra before (black solid line) and after (red solid line) measuring the lifetime of **Dipp-Ar^{Tripp2}** using TCSPC.

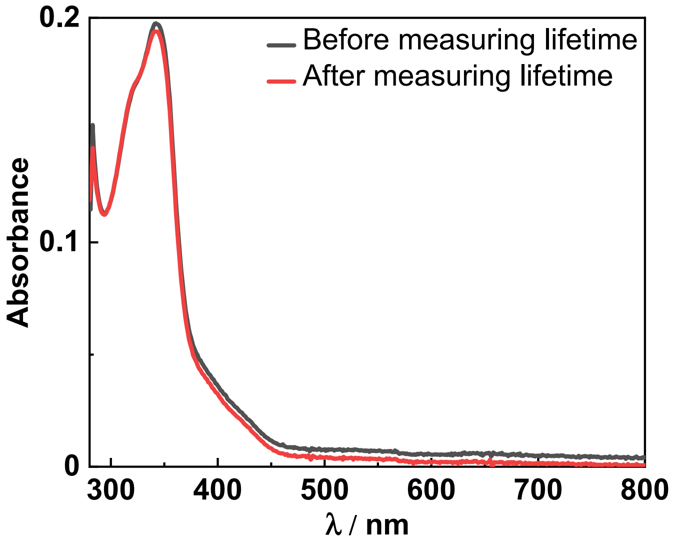

Fig. S45. UV-vis spectra before (black solid line) and after (red solid line) measuring the lifetime of **Dipp-Ar^{Dipp2}** using TCSPC.

Table S12. Sample concentrations used for lifetime measurements of Cu(RNacNac)(CN-Ar^{X2}) complexes.

	Concentration / M
**Cy-Ar^{Ph2}**	4.7×10^{-5}
**2,6-Dmp-Ar^{Ph2}**	1.7×10^{-5}
**Dipp-Ar^{Ph2}**	4.0×10^{-5}
**Dipp-Ar$^{(3,5\text{-}Dmp)2}$**	1.3×10^{-5}
**Dipp-Ar^{Mes2}**	1.1×10^{-5}
**Dipp-Ar^{Tripp2}**	7.2×10^{-5}
**Dipp-Ar^{Dipp2}**	4.8×10^{-5}

Table S13. Summary of the steric parameters determined from solid angle analysis.[a]

	NacNac G_M(L) (%)	Isocyanide G_M(L) (%)	G_M(complex) (%)
**Ph-Ar^{Ph2}**	42.71	27.13	69.62
**Cy-Ar^{Ph2}**	50.35	26.96	75.59
**2,6-Dmp-Ar^{Ph2}**	50.07	27.45	77.52
	49.46	27.21	76.42
	49.50	27.34	76.84
	49.44	27.53	76.96
**Dipp-Ar^{Ph2}**	58.16	26.92	84.30
**Dipp-Ar$^{(3,5\text{-Dmp})2}$**	59.25	28.94	85.81
	58.67	29.98	85.87
**Dipp-Ar^{Mes2}**	60.68	27.84	86.11
**Dipp-Ar^{Tripp2}**	59.64	32.47	89.10
**Dipp-Ar^{Dipp2}**	57.93	29.07	85.65

[a] In **2,6-Dmp-Ar^{Ph2}** and **Dipp-Ar$^{(3,5\text{-Dmp})2}$**, which each have more than one crystallographically independent molecule in the asymmetric unit, separate values are reported for each independent molecule.

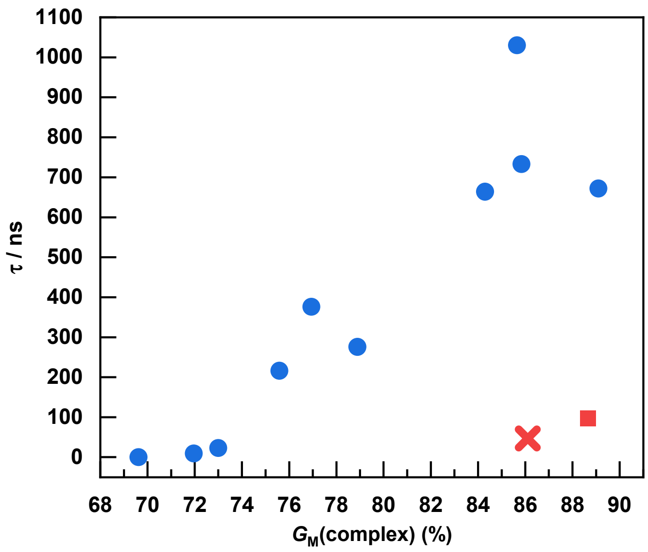

Fig. S46. Excited-state lifetimes of Cu(RNacNac)(CN-Ar^{X2}) complexes plotted vs. Solid Angle parameter G_M(complex) (%).[13] For the complex **Ph-Ar^{Ph2}**, where there was no PL observed, the lifetime was taken to be 0. For **Dmp-Ar^{Ph2}** and **Dipp-Ar$^{(3,5\text{-dmp})2}$**, which have more than one crystallographically independent molecule, the average of the Σ%V_{bur} values is used in the plot. This plot also includes four Cu(CyNacNac)(CN-Ar^{X2}) complexes previously reported by our group.[7] The extreme outliers **Dipp-Ar^{Mes2}** (red "x") and **Cy-Ar$^{(3,5\text{-D}t\text{bup})2}$** (red square, previously reported) are denoted.

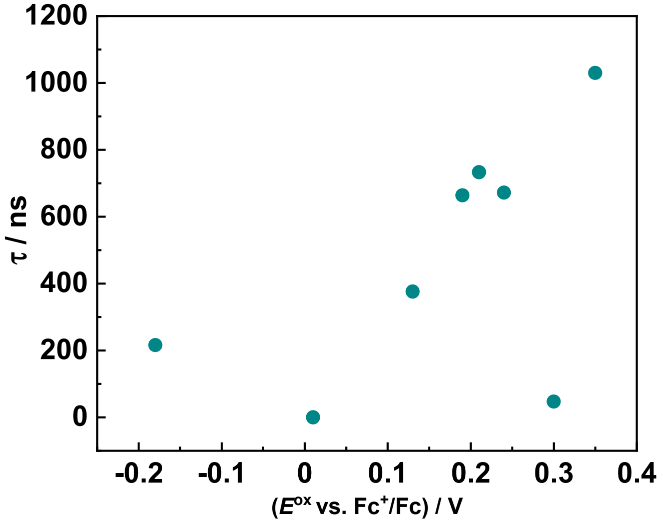

Fig. S47. Excited-state lifetimes plotted vs. the E^{ox} of Cu(RNacNac)(CN-Ar^{X2}) complexes. For the complex **Ph-Ar^{Ph2}**, where there was no PL observed, the lifetime was taken to be 0.

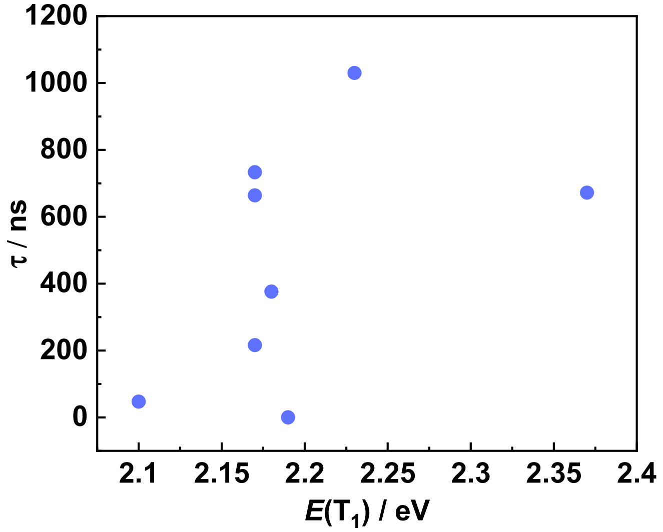

Fig. S48. Excited-state lifetimes plotted vs. the $E_{0,0}$ (determined from the $\lambda_{0,0}$ peak in the 77 K PL spectrum) of Cu(RNacNac)(CN-Ar^{X2}) complexes. For the complex **Ph-Ar^{Ph2}**, where there was no PL observed, the lifetime was taken to be 0.

Supplementary information references

1 A. B. Pangborn, M. A. Giardello, R. H. Grubbs, R. K. Rosen and F. J. Timmers, *Organometallics*, 1996, **15**, 1518–1520.
2 T. Tsuda, T. Hashimoto and T. Saegusa, *J. Am. Chem. Soc.*, 1972, **94**, 658–659.
3 L.-M. Tang, Y.-Q. Duan, X.-F. Li and Y.-S. Li, *J. Organomet. Chem.*, 2006, **691**, 2023–2030.
4 I. El-Zoghbi, S. Latreche and F. Schaper, *Organometallics*, 2010, **29**, 1551–1559.
5 M. Stender, R. J. Wright, B. E. Eichler, J. Prust, M. M. Olmstead, H. W. Roesky and P. P. Power, *J. Chem. Soc. Dalton Trans.*, 2001, 3465–3469.
6 M. Tanabiki, K. Tsuchiya, Y. Kumanomido, K. Matsubara, Y. Motoyama and H. Nagashima, *Organometallics*, 2004, **23**, 3976–3981.
7 D. Kim and T. S. Teets, *J. Am. Chem. Soc.*, 2024, **146**, 16848–16855.
8 Inorganic Syntheses, Volume 37 | Wiley, https://www.wiley.com/en-us/Inorganic+Syntheses%2C+Volume+37-p-9781119477730, (accessed May 13, 2025).
9 K. Suzuki, A. Kobayashi, S. Kaneko, K. Takehira, T. Yoshihara, H. Ishida, Y. Shiina, S. Oishi and S. Tobita, *Phys. Chem. Chem. Phys.*, 2009, **11**, 9850–9860.
10 P. G. Seybold and M. Gouterman, *J. Mol. Spectrosc.*, 1969, **31**, 1–13.
11 G. M. Sheldrick, *Acta Crystallogr. A*, 2008, **64**, 112–122.
12 L. Falivene, Z. Cao, A. Petta, L. Serra, A. Poater, R. Oliva, V. Scarano and L. Cavallo, *Nat. Chem.*, 2019, **11**, 872–879.
13 I. A. Guzei and M. Wendt, *Dalton Trans.*, 2006, 3991–3999.
